# Possible northern persistence of Siebold’s beech, *Fagus crenata,* at its northernmost distribution limit on an island in Japan Sea: Okushiri Island, Hokkaido

**DOI:** 10.3389/fpls.2022.990927

**Published:** 2022-12-15

**Authors:** Keiko Kitamura, Kanji Namikawa, Yoshiaki Tsuda, Makoto Kobayashi, Tetsuya Matsui

**Affiliations:** ^1^Hokkaido Research Centre, Forestry and Forest Products Research Institute, Sapporo, Japan; ^2^Biological Laboratory, Hokkaido University of Education, Sapporo, Japan; ^3^Sugadaira Montane Research Center, University of Tsukuba, Ueda, Japan; ^4^Department of Education and Culture, Echigo-Matsunoyama Museum of Natural Science, Tokamachi, Japan; ^5^Center of Biodiversity and Climate Change, Forestry and Forest Products Research Institute, Tsukuba, Japan; ^6^Faculty of Life and Environmental Sciences, University of Tsukuba, Tsukuba, Japan

**Keywords:** the northernmost geographic range, chloroplast DNA, nuclear SSR, refugia, structure

## Abstract

Siebold’s beech, *Fagus crenata*, is widely distributed across the Japanese Archipelago and islands in Japan Sea. Similar to the northern limit of the geographical distribution of *F. crenata* on the mainland of Hokkaido, the northern limit of the distribution of *F. crenata* on islands in the Japan Sea is observed on Okushiri Island (ca 42°*N*). To understand the genetic relationships of *F. crenata* on Okushiri Island, we examined chloroplast (cp) DNA haplotypes and 11 nuclear microsatellite (SSR) loci among 1,838 individuals from 44 populations from Okushiri Island, mainland Hokkaido, and the northern part of the Tohoku region on Honshu Island. We identified 2 cpDNA haplotypes, which represent not only populations on the Japan Sea coast but also those on the Pacific coast and this suggested the Okushiri Island populations might not be formed by single colonization. Genetic diversity of the Okushiri Island populations of nuclear SSR was not lower than the mainland and the STRUCTURE analysis revealed the Okushiri Island individuals were admixed between Hokkaido and Tohoku clusters. Approximate Bayesian computation inferred that divergence between Tohoku and Hokkaido, and admixture between two populations which generated Okushiri populations occurred before the last glacial maximum (LGM), that is, 7,890 (95% hyper probability density (HPD): 3,420 – 9,910) and 3,870 (95% HPD: 431– 8,540) generations ago, respectively. These inferences were well supported by a geological history which suggested an isolation of Okushiri Island from Hokkaido started prior to the Middle Pleistocene. We discuss the possible persistence of *F. crenata* during the last glacial maximum on northern islands in the Japan Sea such as Okushiri Island.

## Introduction

Plant populations on islands differ from mainland populations in terms of ecological and demographic processes such as colonization events, population persistence, and expansion because of their geographical isolation. From a genetic perspective, island populations are assumed to have less diversity than their mainland counterparts due to evolutionary processes such as founder effects, limited gene dispersal, and small population size (Barrett, 1996; Frankham, 1996; Frankham, 1997; Takayama et al., 2013; Stuessy et al., 2014; Takayama et al., 2015; Stuessy, 2020). For example, the initial colonizing event inevitably involves a genetic bottleneck which determines the loss of gene diversity in founder populations (Barrett, 1996). Therefore, newly founded populations carry less gene diversity than source populations; however, repeated gene flow from source populations can compensate for a lack of gene diversity and contribute to long-term population persistence and expansion (Alsos et al., 2015). Theoretically, long-distance gene flow is less frequent than short-distance gene flow, so that spatial isolation and small population size may lead to an erosion of genetic variation and increased interpopulation differentiation (Young et al., 1996). In general, geographical isolation is a major contributor to shallow gene diversity (DeJoode and Wendel, 1992; Chiang et al., 2006; Takayama et al., 2013; Alsos et al., 2015).

On the contrary, several studies of terrestrial plant species revealed that island populations have substantial genetic diversity in spite of their isolated geographic locations which might restrict frequent gene flow (Chiang et al., 2006; Rosas Escobar et al., 2011; Désamoré et al., 2012; García-Verdugo et al., 2013; García-Verdugo et al., 2015). This is thought to be due to the fact that the genetic diversity of island populations is not only related to founder effects but also by different geographic and biological conditions and contexts (Stuessy et al., 2014), such as adaptation and maladaptation to the new environment (St Clair and Howe, 2007; Kuparinen et al., 2010) and chronic changes of gene immigration (Elleouet and Aitken, 2019; Kitamura and Nakanishi, 2021).

Another interesting issue regarding island populations is the colonization history during the species range expansion (Taberlet et al., 1998; Magri et al., 2006; Alsos et al., 2015). Compared to mainland populations, the routes and frequency of dispersal to islands are restricted due to geographic isolation, and long-distance gene dispersal represents the initial stage in the colonization process.

*Fagus crenata* Blume (2n = 24) is monoecious and allogamous, with barochorous and synzoochorous by rodents and birds (Miguchi, 1996). The geographic range of *F. crenata* extends across the Japanese Archipelago, from southern Hokkaido to Kyushu (Peters, 1997), with sharp geographic clines in genetic diversity (Tomaru et al., 1997; Hiraoka and Tomaru, 2009) and morphology (Hagiwara, 1977; Ishii et al., 2018) observed. Our previous study of genetic structure at the northernmost distribution range on mainland Hokkaido discovered a decline in gene diversity toward the northern edge of populations (Kitamura et al., 2015). In addition to the mainland distribution, natural populations of *F. crenata* are observed on off-shore islands in the Japan Sea, with Okushiri Island (ca. 42°N) representing the northernmost distribution of *F. crenata* among such islands (Tatewaki, 1948). The island is reported to have been separated from the mainland in the Middle Pleistocene and never became reattached to the mainland thereafter (Ohshima, 1980). The northward expansion of *F. crenata* after the Last Glacial Maximum (LGM) started about 6,000 years BP on the mainland (Kito and Takimoto, 1999); however, the origins of the northernmost island population of *F. crenata* is still unknown. Long-distance vs. short-distance gene dispersals are driving forces to develop genetic diversity within populations. As tracers of gene flow, neutral molecular markers provide useful information, although care needs to be taken in separating historical connectivity from current gene flow. A combination of maternally inherited chloroplast (cp) genome for angiosperms and bi-parental inherited nuclear genome provides relevant information such as the colonization history because comparisons of uniparentally (cp) vs. biparentally (nuclear) inherited loci can reveal differences in dispersal between seeds and/or pollen (Petit and Excoffier, 2009). Moreover, recent advances in genetic-based population demographic inference make it possible to investigate a colonization history of species from different ancestral lineages, considering time scales (Tsuda et al., 2015).

The aims of this study are: 1) to evaluate the genetic structure of *F. crenata* on Okushiri Island by 44 populations from northern distribution using nuclear and cpDNA variation, 2) to infer the species’ population demography on the island, and 3) to discuss the colonization and northern expansion dynamics of *F. crenata*, considering a possibility of northern persistence of the species during the LGM.

## Materials and methods

### Study sites

**Figure 1:**
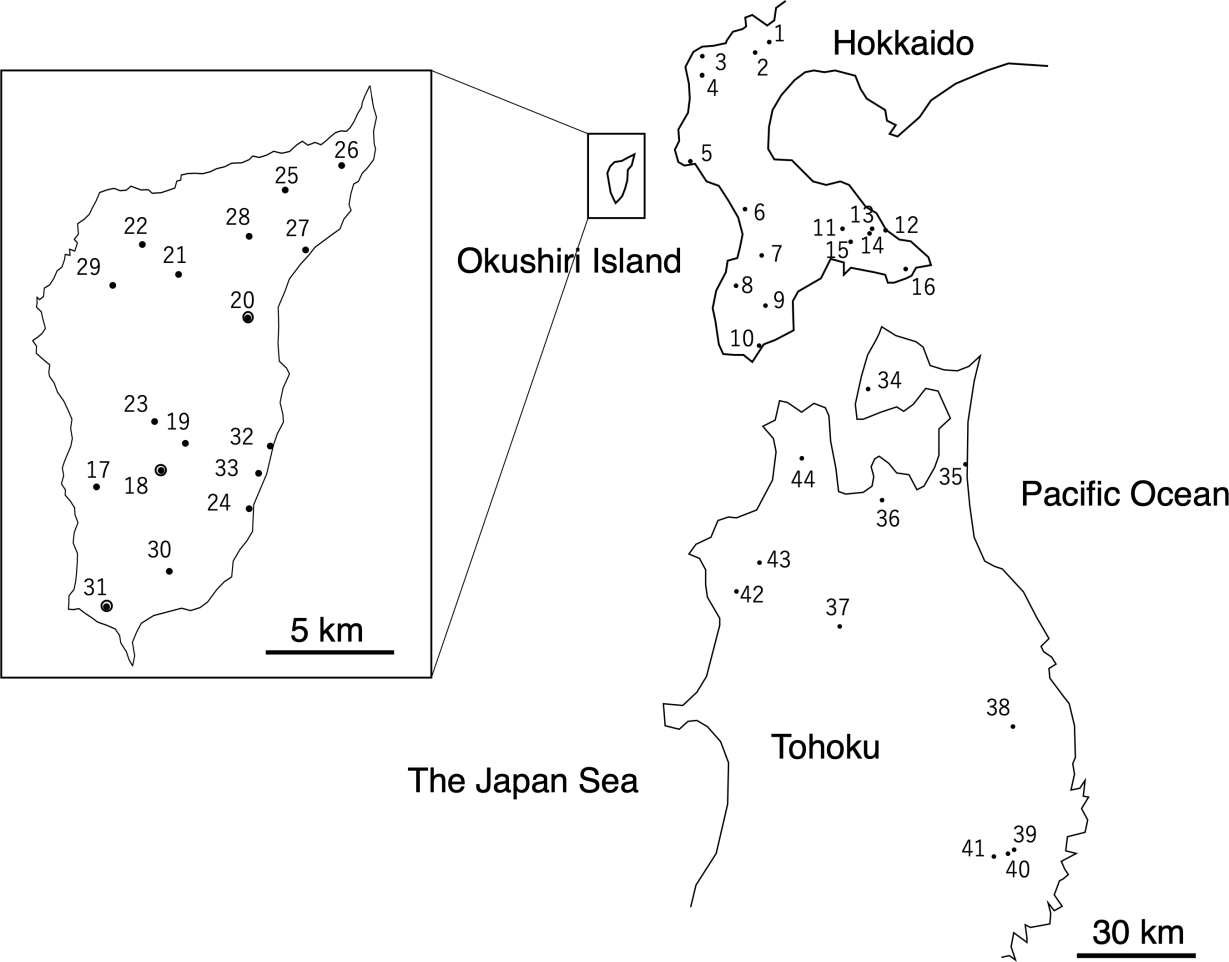
Locations of *Fagus crenata* populations examined in this study (**Supplementary Table 1**) Populations 18, 20, and 31 were referred in the Discussion.

Okushiri Island is located 16 km west of the mainland of Hokkaido in the island-mainland northernmost distribution area of *F. crenata* (**Figure 1**). This Island is a small island of 143 square km, where forest covers 80% of the island, yet the *F. crenata* forest covers approximately 60% of the forested area (Namikawa et al., 2021), and its stand volume is around 60 cubic meters per hectare (Tatewaki, 1948).

In terms of the vegetation classification, the beech forests on Okushiri Island showed diverse floristic features from subarctic to warm temperate elements which is partly due to the warm current (Namikawa et al., 2021). The major vegetation type of beech forest on the island includes species that frequently appear in beech forests on the Japan Sea coast of Hokkaido and northern Tohoku. Another vegetation type found on Okushiri Island is associated with the southern part of the Pacific coast of Hokkaido, which has a short snow-cover period. Thus, despite the small size of the island, regional differences in species composition seen in mainland Hokkaido and Tohoku were condensed in the beech forests on Okushiri Island (Namikawa et al., 2021).

We selected 17 populations from across the *F. crenata* forests on the island, 16 populations from the mainland of Hokkaido, and 11 from the northern Tohoku region of Honshu as study sites, covering most of the species’ distribution in these areas (**Figure 1**, **Supplementary Table 1**). To cover most of the natural distribution, study populations on Okushiri Island were selected from both east- and west-facing slopes of the island, as well as coastal and inland habitats. Populations from mainland Hokkaido and the northern Tohoku region were selected across the contrasting climate conditions of the Pacific coast that shows the dry and low temperature winter, and the Japan Sea coast, which experiences heavy snow fall in winter.

### DNA extraction, chloroplast DNA SNPs and microsatellite genotyping

Mature trees of diameter at breast height (DBH) > 20 cm (11 to 65 trees per population) were chosen at random, and leaves were collected from a total of 1,838 individuals from 44 populations (**Supplementary Table 1**). Collected leaves were kept in a cooling box with ice until they could be stored in the refrigerator or freezer in the laboratory. Total DNA was extracted with the DNeasy Plant Mini Kit (Qiagen K. K., Tokyo, Japan) from 50 mg of leaf sample that was ground into a powder using liquid nitrogen and a Multi-Beads Shocker cell disruptor (Yasui Kikai Co. Ltd., Osaka, Japan).

It has been revealed that there are two chloroplast (cp)DNA haplotypes in these regions, that is, A (GenBank: AB046492.1) and B (GenBank: AB046493.1) (Fujii et al., 2002). We determined the cpDNA haplotypes of each population by SNPs according to the method described by Katai et al. (2014); Takahashi et al. (2022). The first polymerase chain reaction (PCR) was performed to amplify *trn*K fragment by 0.2 µM final concentration of each forward (5’-TTATTCTTAGCGGATCGGTCCA-3’) and reverse (5’-CCGTGCTTGCATCTTTCATTG-3’) primer using Multiplex PCR Kit (Qiagen K. K., Tokyo, Japan) containing 10 to 30 ng of genomic DNA in a final volume of 6 µl. Amplification condition was 95°C for 15 min., 35 cycles of [94°C for 30 sec., 56°C for 90 sec., 72°C for 2 min.], 72°C for 10 min, then hold at 4°C. Amplicons were treated with 0.8 U of *Exo*I (Takara Bio Inc., Shiga, Japan) and 2 U of Shrimp Alkaline Phosphatase (SAP, Takara Bio Inc., Shiga, Japan) with *Exo*I buffer (Takara Bio Inc., Shiga, Japan) to remove unincorporated primers and dNTPs for 1 hour at 35°C followed by 15 min. at 75°C for inactivation of enzyme, then hold at 4°C. The single-base primer extension was performed using SNaPshot Multiplex Kit (Thermo Fisher Scientific K. K., Tokyo, Japan) and *trn*K1754 primer (5’- T^14^CTAGCATTTGACTCCGCACCACTGAAG -3’). The total volume of the reaction mix was 7 µl which contained 1 µl of SNaPshot Master Mix, 0.7 µl of 2 µM primer, 1 µl of BigDye Terminator 5 x Sequencing buffer (Thermo Fisher Scientific K. K., Tokyo, Japan), and 2 µl of the first PCR product. The extension was performed under condition of 25 cycles of [96°C for 10 sec., 50°C for 5 sec., 60°C for 30 sec.], then hold at 4°C. SNaPshot extension reaction was treated with 1 U of SAP (Takara Bio Inc., Shiga, Japan) to remove unincorporated primer and ddNTPs for 1 hour at 35°C followed by 15 min. at 75°C for inactivation of enzyme, then hold at 4°C. The fragments were detected with ABI PRISM 3130*xl* Genetic Analyzer (Thermo Fisher Scientific K. K., Tokyo, Japan) using POP-7 polymer and 36 cm capillary. An aliquot of SNaPshot reaction (1 µl) was mixed with 9.8 µl of Hi-Di Formamide (Thermo Fisher Scientific K. K., Tokyo, Japan) and 0.2 µl of GeneScan 120 LIZ dye Size Standard (Thermo Fisher Scientific K. K., Tokyo, Japan). SNPs were determined by GENESCAN for Windows (Thermo Fisher Scientific K. K., Tokyo, Japan). We first analyzed four individuals from each population to determine cpDNA haplotypes. When four individuals from the same population showed the same haplotypes, the cpDNA haplotype of the population was determined to be monomorphic. When different haplotypes were detected from four individuals from the same population, we further analyzed 12 to 22 individuals from that population to determine the cpDNA haplotype ratio of the population. The cpDNA haplotype are fixed in most of the populations of this species, and we analyzed the limited number of individuals to detect at least 6% of polymorphism.

Eleven nuclear microsatellite (SSR) primer pairs were employed to identify genotypes of all individuals for the following loci: mfc2, mfc12 (Tanaka et al., 1999), FS1-03, FS4-46 (Pastorelli et al., 2003), sfc7, sfc18, sfc36, sfc378, sfc1063, sfc1105, and sfc1143 (Asuka et al., 2004), according to the method described in (Kitamura et al., 2015). PCR was performed with the Multiplex PCR Kit (Qiagen K. K., Tokyo, Japan) containing 10 to 30 ng of genomic DNA in a final volume of 10 µl. Amplification condition was 95°C for 15 min., 35 cycles of [94°C for 30 sec., 57°C for 90 sec., 72°C for 1 min.], 60°C for 30 min, then hold at 15°C. PCR products (1 µl) was mixed with 10 µl Hi-Di Formamide (Thermo Fisher Scientific K. K., Tokyo, Japan) and 0.15 µl of GeneScan 600 LIZ dye Size Standard v2.0 (Thermo Fisher Scientific K. K., Tokyo, Japan) for estimating DNA fragment sizes. The length of amplified fragments was analyzed using the ABI PRISM 3130*xl* Genetic Analyzer using POP-7 polymer, 36 cm capillary, and GENESCAN for Windows (Thermo Fisher Scientific K. K., Tokyo, Japan).

The number of individuals used for analyses are shown in **Supplementary Table 1**.

### Data analyses

The genetic diversity parameters within each population were evaluated by determining the expected heterozygosity (*H*_E_) (Nei 1987), allelic richness (*R*_S_) (El Mousadik and Petit, 1996), and the fixation index (*F*_IS_) (Wright 1965) using the FSTAT 2.9.3 computer program (hereafter, FSTAT) (Goudet, 2001). Significant deviations in *F*_IS_ values from 0 were estimated by 95% confidence intervals (CI) obtained through 999 permutations of bootstrapping using GENODIVE (Meirmans and Van Tienderen, 2004). The degree of genetic differentiation among the populations was evaluated by Nei’s *G’*_ST_. The number of alleles and the effective number of alleles within a region were calculated by GENODIVE. Frequency of null allele were calculated by CERVUS (Marshall et al., 1998).

To determine the coancestry composition among populations and admixtures, we employed multilocus model-based cluster analysis using STRUCTURE 2.3.4 (hereafter, STRUCTURE) (Pritchard et al., 2000). All of the runs consisted of 30,000 Markov chain Monte Carlo (MCMC) generations, after a burn-in period of 70,000 iterations. We confirmed that these settings for the length of the MCMC generations and burn-in period are sufficient to obtain valid genetic structures by comparing the results from initial runs with those involving longer MCMC generations. This analysis was based on the LOCPRIOR model described by Hubisz et al. (2009), an admixture model, and the correlated allele frequencies model (hereafter, the F-model) described by Falush et al. (2003). Ten runs were performed for each value of *K*, ranging from 1 to 10; as we confirmed that the genetic structure among population could be detected at *K* < 10 in the pilot run. CLUMPAK server (Kopelman et al., 2015) was employed to evaluate the probability of the data [LnP (D)] for each *K* and to calculate Δ*K* according to the method described by Evanno et al. (2005), and to evaluate multimodality among runs and major clustering patterns at each *K*.

In addition, principal coordinate analysis (PCoA) of 44 populations based on the allele frequencies of 11 SSR loci, as well as Mantel test for isolation by distance between the pairwise genetic distance and geographic distances were conducted by GENODIVE (Meirmans and Van Tienderen, 2004). Phylogenetic networks among populations on Okushiri Island was obtained from neighbor-net split network method based on pairwise genetic distance using SplitsTree4 (Huson and Bryant, 2006).

### Inference of past population demographic history using the approximate bayesian computation

**Figure 2:**
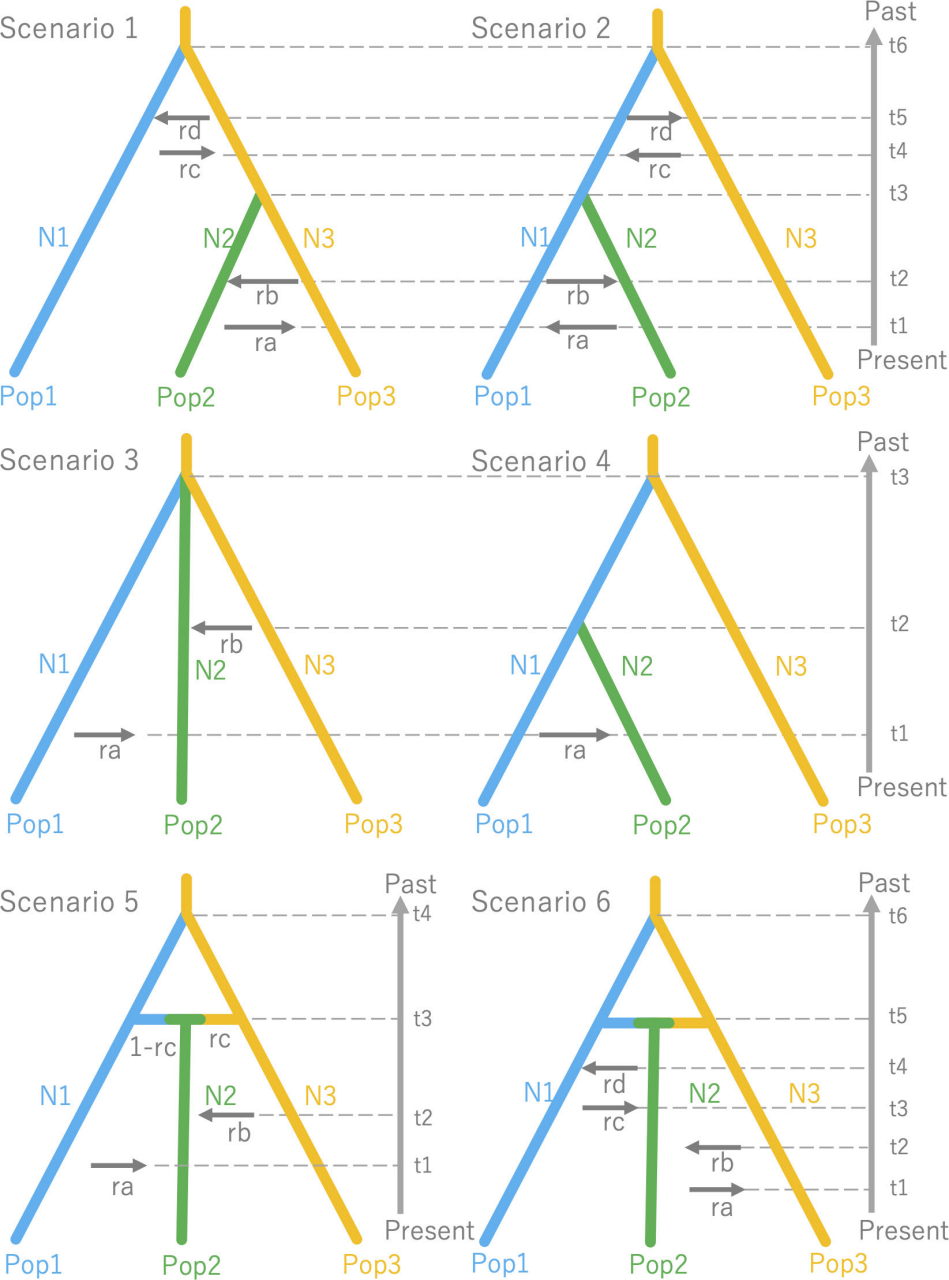
Six simple population demography scenarios used for Approximate Bayesian Computation (ABC).

To infer past demographic history of colonization, we employed the Approximate Bayesian Computation (ABC) approach and used software DIYABC v2.1 (Cornuet et al., 2008; Cornuet et al., 2014). To make the scenarios in the ABC analysis simple, we focused on three representative populations: (1) the mainland of Hokkaido, (2) Okushiri Island, and (3) Tohoku region. These representative populations were made as follows. According to the results of the above-mentioned STRUCTURE analysis, three clusters were detected that corresponded to these three areas at *K* = 3. Then the 50 individuals which showed the highest ancestry within each cluster were selected, and named to Pop1 (the mainland of Hokkaido), Pop2 (Okushiri Island) and Pop3 (Tohoku region). These three representative populations, that is a total of 150 individuals, were examined six simple population demography scenarios (**Figure 2**).

Since DIYABC requires a population which can be traced back to an ancestral population, we set Pop3 to be the ancestor because the Tohoku region showed higher genetic diversity than the mainland of Hokkaido (Takahashi et al., 1994; Hiraoka and Tomaru, 2009) (see also, **Table 1**). This treatment is reasonable according to Tsuda et al. (2015). In these scenarios, t1 to t6 represents the time scale, measured by generation time, N1 to N3 represents the effective population size of the corresponding populations (Pop1, 2 and 3) and ra to rd represents the ratio of admixture. Although the DIYABC basically does not take gene flow after the split into the scenario, a recent study by Chapuis et al. (2020) modified the DIYABC to allow symmetrical admixture for two population data and we extended this approach to make it more flexible for multiple population data, making it possible to put directional admixture (e.g., from Pop1 to 2, from Pop2 to 1) in the scenarios. Thus, this directional admixture can be analogues to gene flow. The inferred value of admixture parameter (r#) from Pop1 to Pop2 could be considered as the relative amount of gene flow at t# and 1-r# was the value of relative amount of gene flow within a parental population for the admixture (gene flow).

**Table 1:** Genetic parameters for each region.

Region	*H*_E_		Effective number of alleles		*G’*_ST_		*F*_IS_		Number of alleles	
Hokkaido		0.776 (0.681 - 0.858)		5.639 (3.872 - 7.759)		0.016 (0.012 - 0.022)		0.010 (-0.008 - 0.031)		22.273 (17.000 - 28.455)
Okushiri Island		0.778 (0.685 - 0.857)		5.332 (3.804 - 7.006)		0.023 (0.016 - 0.034)		0.012 (-0.007 - 0.042)		22.273 (16.909 - 27.818)
Tohoku		0.797 (0.722 - 0.862)		5.795 (4.019 - 7.841)		0.036 (0.014 - 0.079)		0.033 (0.003 - 0.076)		23.455 (17.545 - 30.182)

95% confidence intervals are in parentheses.

Scenario 1: Hierarchical split model I: Pop2 was merged to Pop3 at t3 and Pop1 was merged to Pop3 at t6, assuming populations on the Okushiri Island were split from Tohoku region. The directional admixture from Pop2 to Pop3 (ra), Pop3 to Pop2 (rb), from Pop1 to Pop3 (rc), and from Pop3 to Pop1 (rd) were set at t1, t2, t4, and t5, respectively. As we did not set any orders of these time scale for the admixtures, the time scale order relied on the inference (e.g., t2 could be earlier than t1) and it was the same in the following Scenarios (2-6).

In Scenarios 3, 4, and 5, the unidirectional admixture was adopted whilst populations on Okushiri Island were assumed to be too small to influence mainland populations by emigrant genes.

Scenario 2: Hierarchical split model II: Pop2 was merged to Pop1 at t3 and Pop1 was merged to Pop3 at t6, assuming populations on Okushiri Island were split from the mainland of Hokkaido. Similar to Scenario 1, the directional admixtures between populations after the splitting were set.

Scenario 3: Simple split model with admixture: all 3 populations diverged at the same time at t3, allowing admixture from Pop1 to Pop2 (ra) and Pop3 to Pop2 (rb) at t1 and t2, respectively.

Scenario 4: Hierarchical split model III: The hierarchical split pattern was similar to Scenario 2. However, we assumed only one directional admixture from Pop1 to Pop2 (ra) at t1.

Scenario 5: Isolation with admixture model I: Pop1 was split from Pop3 at t4 and Pop2 was created by admixtures from Pop3 to Pop2 (rc), and from Pop1 to Pop2 (1-rc) at t3. The secondary directional admixture from Pop1 to Pop2 (ra) and from Pop3 to Pop2 were set at t1 and t2, respectively.

Scenario 6: Isolation with admixture model II: Although this scenario was similar to Scenario 5, we assumed all possible combinations of secondary directional admixture from Pop2 to Pop3 (ra), from Pop3 to Pop2 (rb), from Pop1 to Pop2 (rc), from Pop2 to Pop1 (rd), at t1, 2, 3 and 4, respectively.

The prior distributions of these parameters were shown in **Supplementary Table 2**. We employed the higher mutation rate from the generalized stepwise mutation model (GSM) (Estoup et al., 2002) and the lower rate for single nucleotide indels (SNI) as mutation models of SSRs. To summarize the observed and simulated data, the mean values for expected heterozygosity (*H*_E_), the number of alleles (*A*), and the variance of allele size were used as summary statistics for each of the three populations. *H*_E_, *A*, variance of allele size, classification index (analogues to genotype likelihood) and *F*_ST_ were the summary statistics for each of the population pairs. One million simulations were run for each scenario. After all the simulations had been run, the most likely scenario was determined by comparing the posterior probabilities using the logistic regression method. The goodness of fit of the scenario was assessed by the option ‘model checking’ with PCA in DIYABC, which measures the discrepancy between data sets simulated with the prior distributions of parameters and the observed data as well as data sets from the posterior predictive distribution in the scenario.

## Results

### cpDNA polymorphism

**Figure 3:**
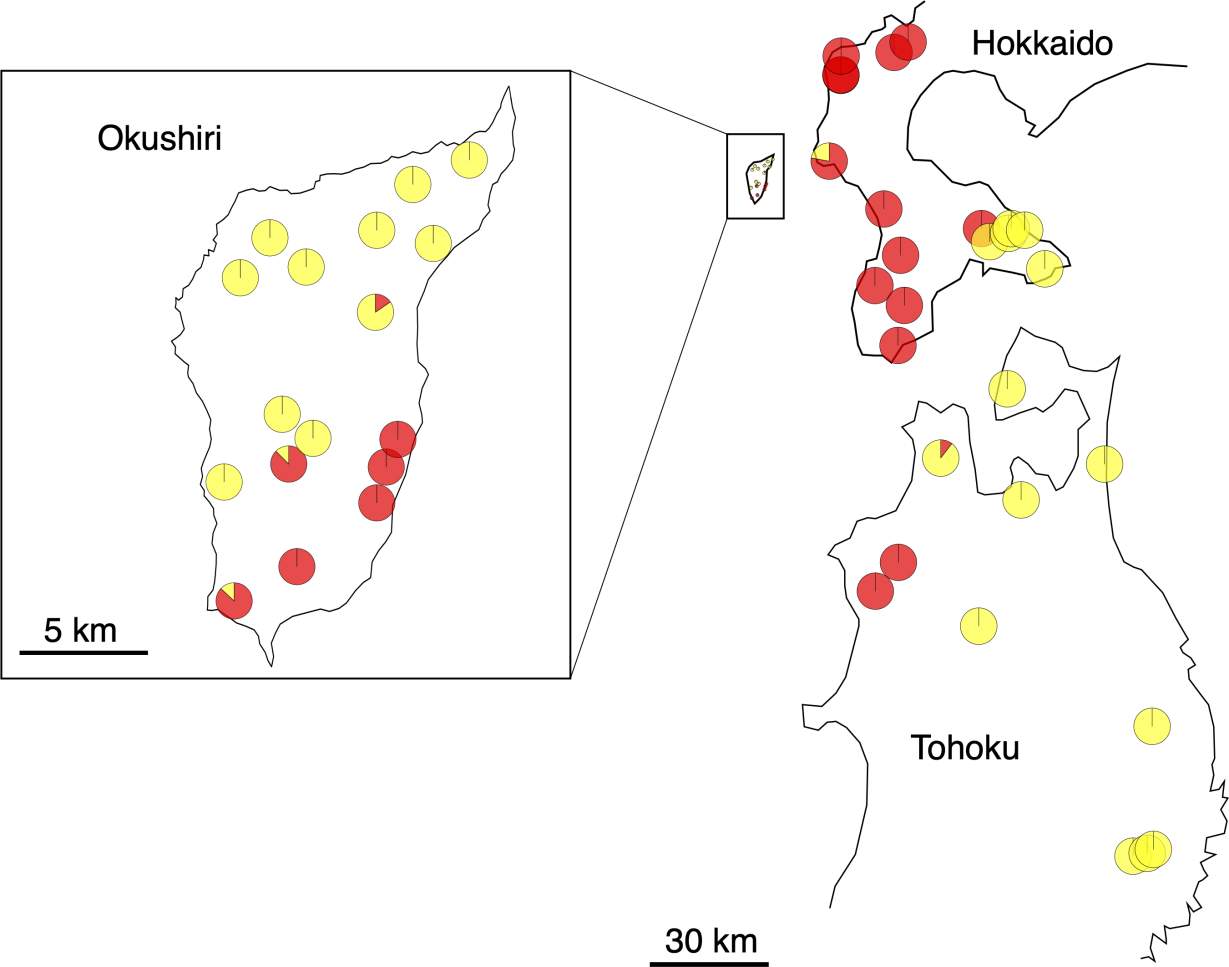
Pie diagrams of cpDNA haplotypes. Red and yellow represent haplotype A and B, respectively.

Two haplotypes were distinguished; namely, A (GenBank: AB046492.1) and B (GenBank: AB046493.1) (Fujii et al., 2002), among the populations studied (**Supplementary Tables 1, 3**, **Figure 3**). The population was determined to be fixed to either haplotype, when the first analysis of 4 individuals showed the same haplotypes. An additional 12 to 22 individuals were analyzed at populations with mixed haplotypes. Most of the populations were fixed to either haplotype; 16 populations to haplotype A and 23 populations to haplotype B. Most of the populations in Hokkaido were fixed to haplotype A, but most of those on Okushiri Island and in Tohoku were fixed to haplotype B. The other 5 populations included both haplotypes, that is, 1 population in Hokkaido, 3 populations on Okushiri Island, and 1 population in Tohoku. However, the ratio of haplotype A/B differed among populations (**Supplementary Table 1**, **Figure 3**). The lower representative haplotype in each region showed specific localization; namely, haplotype B in the southeastern part of Hokkaido, haplotype A in the southeastern part of Okushiri Island, and haplotype A in the northwestern part of Tohoku (**Figure 3**).

### Nuclear gene diversity by SSR

**Figure 4:**
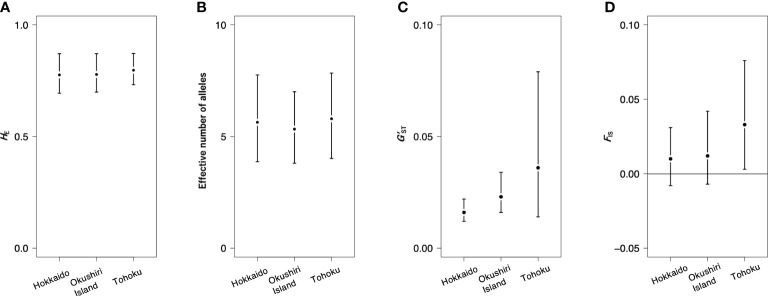
Genetic diversity parameters for 3 regions. **(A)** *H*_E_, **(B)** effective number of alleles, **(C)** *G’*_ST_, and **(D)** *F*_IS_.

The total heterozygosity (*H*_T_) for all 44 populations was 0.783, and the genetic differentiation for all populations (*G’*_ST_) was 0.026. The *H*_E_ values of Hokkaido, Okushiri Island, and Tohoku were 0.776 (values for each population ranges from 0.711 to 0.792), 0.778 (from 0.726 to 0.792), and 0.797 (from 0.731 to 0.800), respectively (**Table 1**, **Supplementary Table 1**). The effective number of alleles of Hokkaido, Okushiri Island, and Tohoku were 5.639 (each population value ranges from 4.811 to 6.338), 5.332 (from 4.576 to 6.377), and 5.795 (from 4.848 to 6.809), respectively (**Table 1**, **Supplementary Table 1**). The *G’*_ST_ for Hokkaido, Okushiri Island, and Tohoku were 0.016, 0.023, and 0.036, respectively (**Table 1**). *F*_IS_ of Hokkaido and Okushiri Island did not show significant deviation from zero, while that of Tohoku was positively deviated from zero (**Table 1**). The positively significant *F*_IS_ was observed at one population from Hokkaido and Okushiri Island, and 4 populations from Tohoku; while negatively significant *F*_IS_ was observed at one population from Okushiri Island (**Supplementary Table 1**). There were no significant regional differences in genetic diversity parameters, such as *H*_E_, the effective number of alleles, *G’*_ST_, and *F*_IS_, among Hokkaido, Okushiri Island, and the Tohoku region (**Table 1**, **Supplementary Table 1**, **Figure 4**). The allelic richness ranged from 7.153 to 8.007, from 6.677 to 8.045, and from 7.056 to 8.325, for Hokkaido, Okushiri Island, and Tohoku, respectively (**Supplementary Table 1**). The number of alleles for Hokkaido, Okushiri Island, and Tohoku were 22.273 (each population value ranged from 7.182 to 13.364), 22.273 (from 7.818 to 13.636), and 23.455 (from 11.091 to 14.364), respectively (**Table 1**, **Supplementary Table 1**). High frequency of null allele (0.278) along with a significant deviation from Hardy-Weinberg equilibrium was estimated in mfc12 at population 8 (**Supplementary Table 4**), however, we believe that it has little influence for the analysis.

### Coancestry composition among population

Although the results from STRUCTURE analysis revealed that Δ*K* was the highest when *K* = 2 (**Supplementary Figure 1**), the Ln P(D) increased with *K*. However, variances of Ln P(D) values among runs were larger after *K* = 4. As regards Δ*K*, even though a hierarchical population scenario is not likely, the highest value can be detected at the uppermost hierarchical level of the population structure (Evanno et al., 2005). Similarly, whilst the highest Δ*K* is often the case of *K* = 2, further population structure could be revealed in *K* > 2 (Pritchard et al., 2000). In this study, the second highest Δ*K* was obtained at *K* = 4, whereas the bar plots of *K* = 3 and 4 show similar population structures. Therefore, *K* = 3 could be still a meaningful clustering with the variance of Ln P(D) being smaller than *K* > 4.

**Figure 5:**
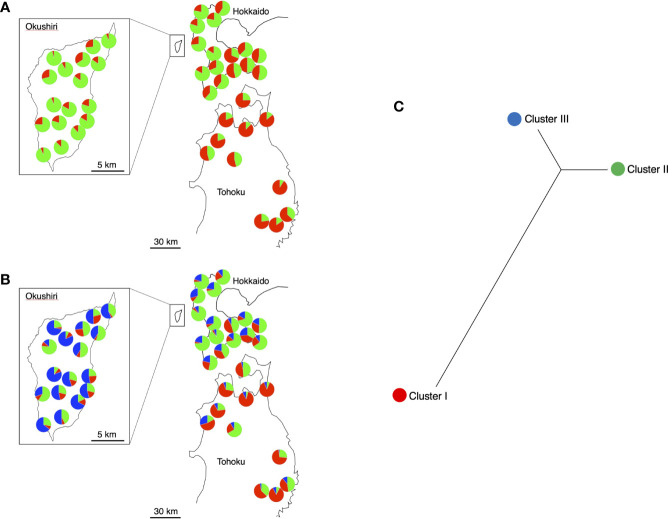
Pie diagrams of STRUCTURE results. The different colors indicate the different ancestral clusters. **(A)** *K* = 2, red and green indicate Cluster I and II, respectively. **(B)** *K* = 3, red, green, and blue indicate Cluster I, II, and III, respectively. **(C)** Relationships among the 3 clusters in *K* = 3 based on *F*_ST_.

We compiled the individual coancestry to each population and drew pie diagrams on geographical map (**Figures 5A, B**).  When *K* = 2 (**Figure 5A**), the F values (analogue to *F*_ST_ between assumed common ancestral population and cluster) were 0.0156 and 0.0275 for Cluster I and II, respectively. The Cluster I was dominant in Tohoku and Cluster II in both Hokkaido and Okushiri Island. Cluster II was more dominant in Okushiri Island than Hokkaido.

When *K* = 3 (**Figure 5B**), the F values were 0.0125, 0.0122 and 0.0141 for Cluster I, II, and III, respectively. Three different clusters dominated the three different regions; that is, Custer I, II, and III for Tohoku, Hokkaido, and Okushiri Island, respectively. Unrooted neighbor-joining cladograms among the 3 clusters based on *F*_ST_ genetic distance matrix showed a closer relationship between Cluster II and III than between Cluster II or III and Cluster I (**Figure 5C**).

### Principle coordinate analysis, isolation-by-distance, and within-island phylogenetic network

**Figure 6:**
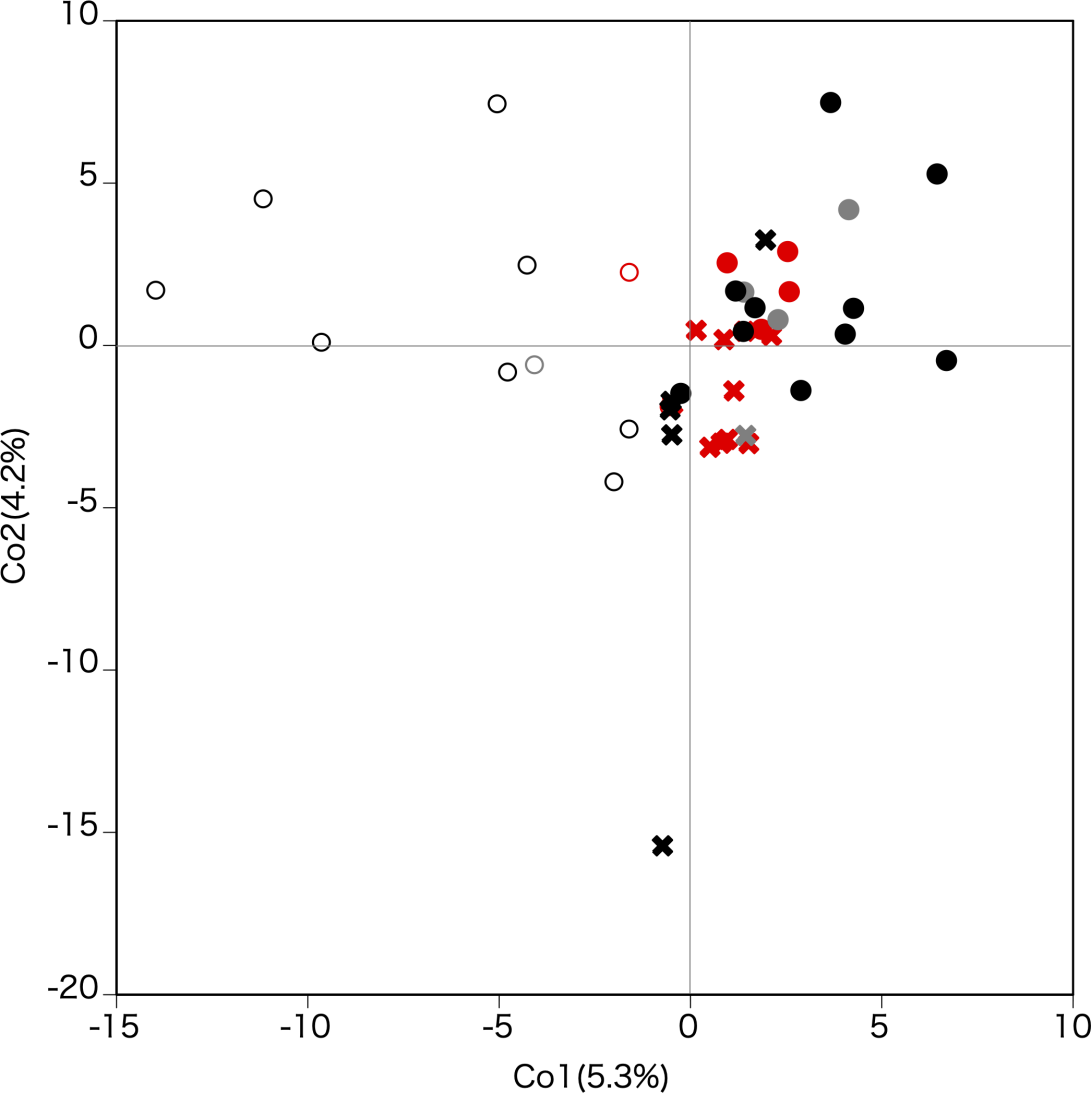
Principle coordinate analysis based on the correlation matrix. The first and second axes represent the first and second main components, respectively. Contribution rates of each axis are given in parentheses. Crosses, filled circles, and open circles indicate Hokkaido, Okushiri Island, and Tohoku, respectively. Red, black, and grey indicate cpDNA haplotype A, B, and mixed A and B populations, respectively.

The results of principle coordinate analysis (PCoA) based on the correlation matrix are shown in **Figure 6**. The first coordinate substantially split the populations in Tohoku from those in Hokkaido and on Okushiri Island. The second coordinate weakly divided populations on Okushiri Island from those in Hokkaido. Also, the second coordinate isolated one population (site 16) located at the tip of the southeastern peninsula of Hokkaido. The cpDNA haplotype differences were reflected in the PCoA diagram (**Figure 6**); populations fixed with haplotype A (red symbols in **Figure 6**) were plotted at the center and those with haplotype B (black) scattered on the periphery of the coordinate plane.

**Figure 7:**
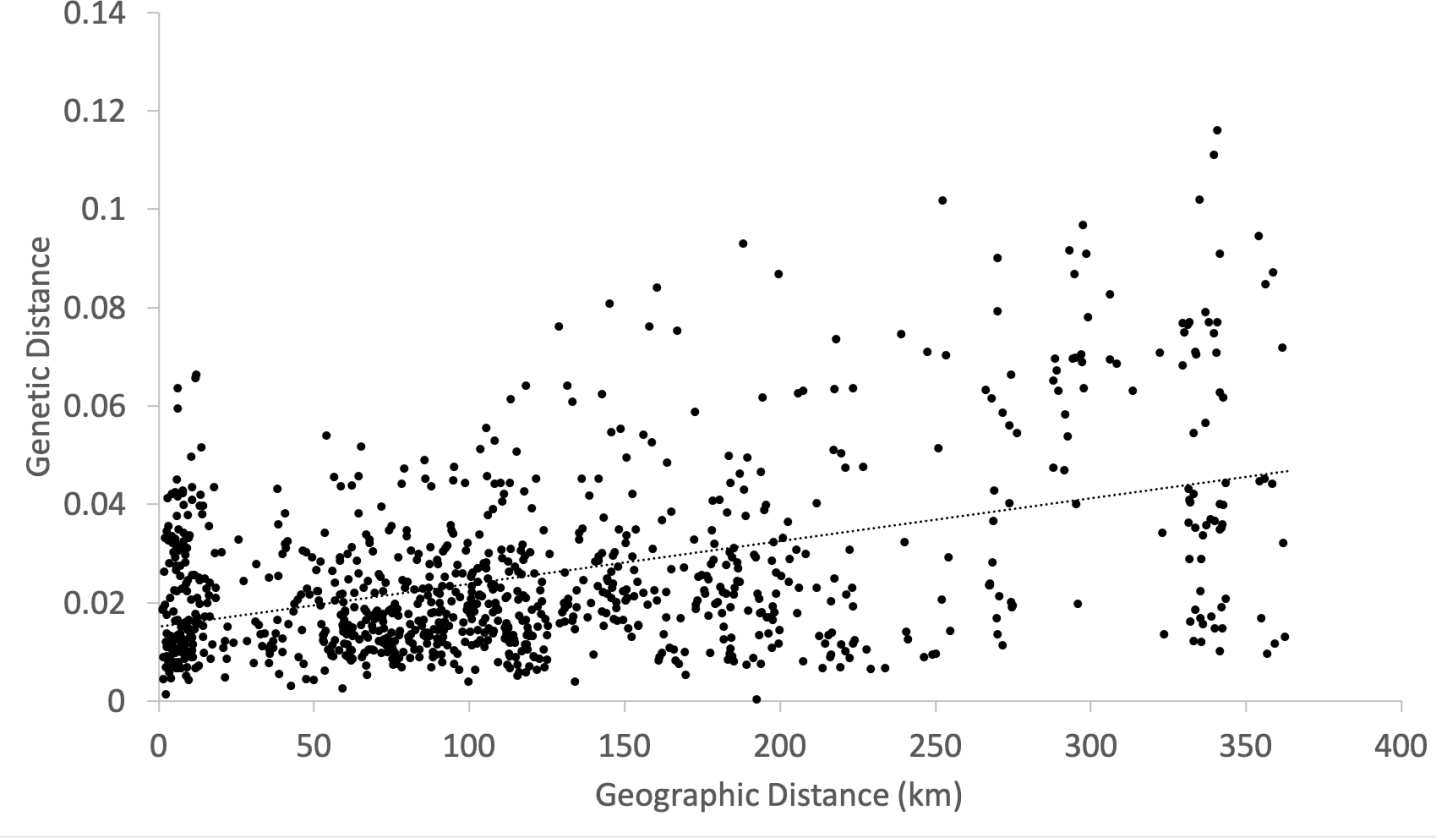
Isolation-by-distance between populations. Genetic distance matrix is based on *F*_ST/_(1 -*F*_ST_). Mantel test by 1,000 permutation detected positive correlation between genetic and geographic distances (*y* = 0.015 + 9E-05*x*, R^2^ = 0.198, *p* = 0.001).

Significant isolation-by-distance was detected between study sites (Mantel test by 1,000 permutations; R^2^ = 0.198, *p* = 0.001) (**Figure 7**).

**Figure 8:**
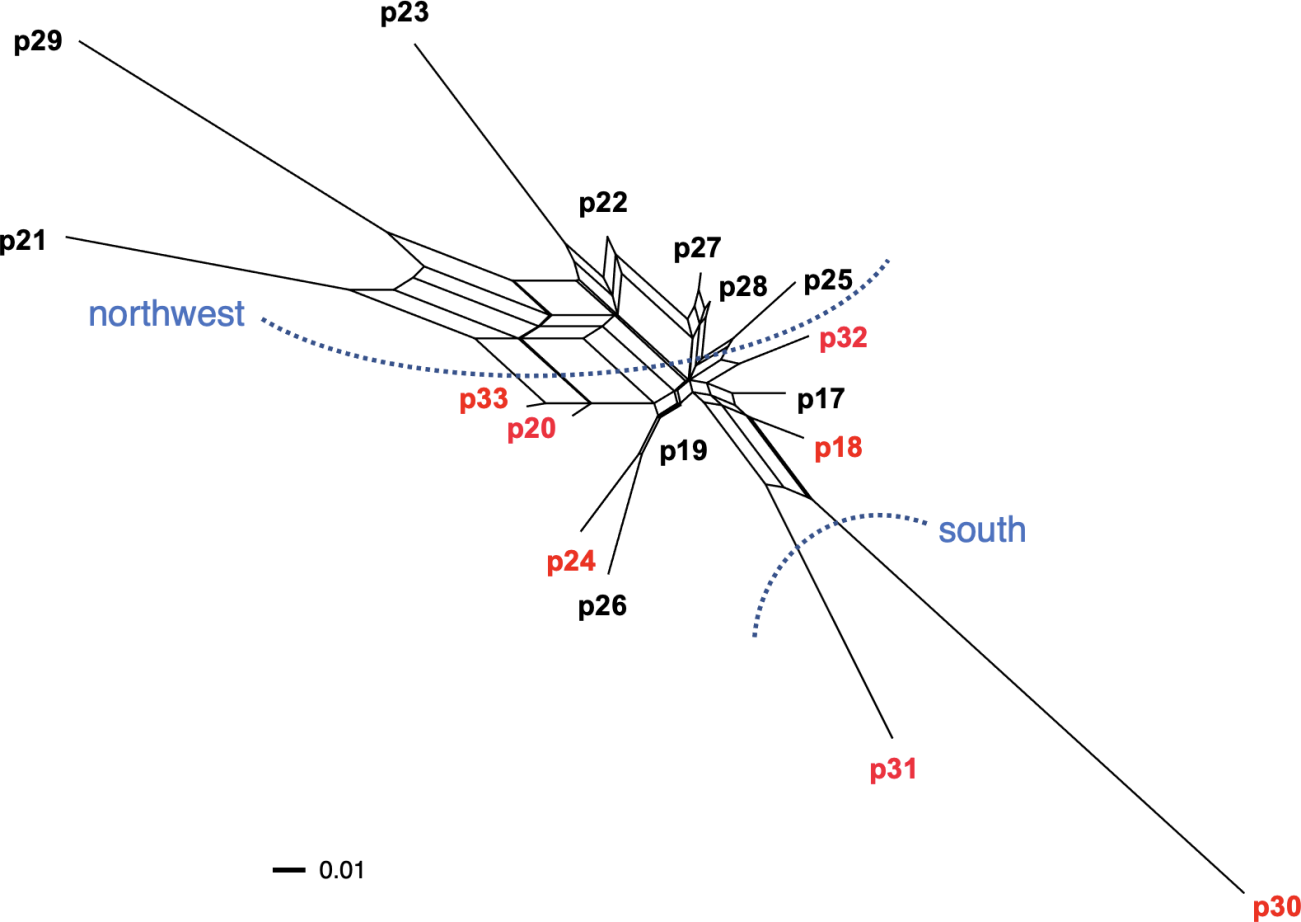
Phylogenetic network among populations within Okushiri Island based on pairwise genetic distance. Population numbers are identical to **Figure 1** and **Supplementary Table 1**. Two distinguished localities, south and northwest, are indicated by blue dotted lines. Population in red letters include cpDNA haplotype A.

A phylogenetic network among populations within Okushiri Island was revealed by neighbor-net split tree (**Figure 8**). The tree distinguished two geographic localities within the island; southern and northwestern populations. The existence of cpDNA haplotype A on the island was associated with phylogenetic network.

### Inference of past population demographic history using the approximate bayesian computation

In DIYABC, the highest posterior probability was found in Scenario 5 (Isolation with admixture model I) (**Table 2**, **Supplementary Figure 2**). The value (0.5388, 95% CI: 0.5305 - 0.5471) was much higher than other scenarios and the 95% CI of Scenario 5 was not overlapped with other scenarios. For Scenario 5, the median values of effective population size of N1 (Pop1), N2 (Pop2), and N3 (Pop 3) were 7,820 (95% hyper probability density (HPD): 4,600-9,640), 5,680 (95% HPD; 2,150-8,640) and 9,180 (95% HPD; 6,450-9,970), respectively (**Supplementary Table 5**). The median values of t1, t2, t3, and t4 were 1,780 (95%HPD: 65.8 -7,500), 4,380 (95%HPD: 998-8,730), 3,870 (95%HPD: 431-8,540) and 7,890 (95%HPD: 3,420-9,910) (**Supplementary Table 5**). The median values of ra, rb and rc were 0.615 (95%HPD: 0.0585-0.983), 0.615 (95%HPD: 0.0402-0.982) and 0.505 (95%HPD: 0.0272-0.975), respectively (**Supplementary Table 5**). The median values of the mean mutation rate of SSR and SNI were 8.68 ×10^-4^ (95% HPD; 4.98×10^-4^-9.99×10^-4^) and 8.69 ×10^-7^ (95% HPD; 1.65×10^-8^-8.50×10^-6^), respectively (**Supplementary Table 5**). The median value of mean P, the parameter of the geometric distribution to generate multiple stepwise mutations was 0.244 (95%HPD; 0.117-0.300) (**Supplementary Table 5**). Only one of the 27 summary statistics of the simulated data was significantly different from the observed data and the PCA yielded a large cloud of data from the prior and observed datasets, centered around a small cluster from the posterior predictive distribution (**Supplementary Figure 3**), suggesting a good fit of the scenario together with high posterior probability.

**Table 2:** Posterior probability of each scenario and its 95% confidence interval based on the logistic estimate by DIYABC.

Scenario	Posterior probability	95% CI (lower - upper)
1	0.0058	0.0029 - 0.0087
2	0.0050	0.0022 - 0.0079
3	0.2201	0.2138 - 0.2265
4	0.1726	0.1670 - 0.1782
5	0.5388	0.5305 - 0.5471
6	0.0576	0.0536 - 0.0616

## Discussion

### Comparable amount of genetic diversity on Okushiri Island to Hokkaido and Tohoku region

The calculated values of *H*_T_ of nuclear SSRs of this study (0.711-0.800, mean value: 0.736) were lower than that for the whole geographical range of *F. crenata* (0.793-0.873, mean value: 0.839) (Hiraoka and Tomaru, 2009). This is because the northern *F. crenata* populations show lower genetic diversity than the geographically central populations (Hiraoka and Tomaru, 2009; Kitamura et al., 2015). Our previous study on the northernmost *F. crenata* populations on mainland Hokkaido revealed a decline in genetic diversity at the northern edge of the species distribution (Kitamura et al., 2015). Although Okushiri Island represents the northernmost distribution of *F. crenata* on islands in the Japan Sea, our data did not show any decline in gene diversity (**Table 1**; **Figure 4**). The northernmost populations on mainland Hokkaido were relatively new founder populations at the northern front of its expansion (Kitamura et al., 2015; Kitamura and Nakanishi, 2021). On the other hand, the high genetic diversity on Okushiri Island indicated that the *F. crenata* populations on that island were not new founders, but might be populations that have persisted for a long time. This was also supported by the fact that *F. crenata* on Okushiri Island were well differentiated with a similar level of *G*_ST_ as Hokkaido and the Tohoku region (**Figure 4**). Thus, although the island is surrounded by ocean, which is a strong practical barrier against gene flow, the effective population size of *F. crenata* remained large enough to compensate for the isolation from the mainland population.

In general, the loss of genetic diversity in island populations can be explained by founder effect during population foundation, limited gene flow and genetic drift due to geographical isolation and small population size (Barrett, 1996; Frankham, 1997). However, our nuclear SSR results revealed that populations on Okushiri Island showed comparable amount of genetic diversity when we focused on Hokkaido and the Tohoku region (**Figures 4A–D**). As a result, the common assumption that island populations have lower genetic diversity than mainland populations (Barrett, 1996; Frankham, 1997) was not applicable to the *F. crenata* populations on Okushiri Island. García-Verdugo et al. (2015) have reported similar results that the nuclear markers do not tend to show lower genetic variation in island populations than in mainland ones.

Relevant amount of genetic diversity on Okushiri Island is also due to the fact that this island is covered with deciduous-broadleaved forest including a substantial population of *F. crenata* (Tatewaki, 1948; Namikawa et al., 2021). Outcrossing by wind-pollination of the species might compensate for the topographic gene flow barriers such as mountain ridges and valleys (Austerlitz et al., 2004; Hu et al., 2008). When genetic connectivity was secured, a large population size might prevent the loss of gene diversity on the island. Also, it is probable that ancestral alleles were retained within the population (Su et al., 2018) as the species has long life span of 250 years on average in Hokkaido (Kitamura et al., 2007) and the oldest tree on Okushiri Island was confirmed to be 374 years by annual ring counts (T. Matsui, M. Kobayashi, and K. Kitamura, unpublished data). Another supportive evidence of persistence of *F. crenata* on Okushiri Island is that the number of private alleles of SSR on Okushiri Island was 16 which was comparative to 14 in Hokkaido. Therefore, the levels of genetic diversity among *F. crenata* on Okushiri Island might be attributable to the large population size, which allows a significant degree of gene flow, and long demographic history, rather than new founder populations from a few individuals. Indeed, although the ABC-based demographic inference showed that the effective population size of Okushiri Island population was smaller than Hokkaido and Tohoku region, the inferred median value was 5,680 (**Supplementary Table 5**), which could be a criterion at least to maintain genetic diversity.

### CpDNA genetic differentiation among *F. crenata* populations in Okushiri Island, Hokkaido, and Tohoku region

Our results from cpDNA haplotypes clearly separated Hokkaido from the Tohoku region (**Supplementary Table 1**, **Figure 3**). A primary difference between *F. crenata* in Hokkaido and the Tohoku region was supported by phytosociology, which categorized the different vegetation types in *F. crenata* forests in Hokkaido and the Japan Sea side of Honshu (Hukusima et al., 1984). Furthermore, the vegetational component of several *F. crenata* populations on the Pacific Ocean side of Hokkaido and the northwestern peninsula of Tohoku were classified into the same category as that on Okushiri Island (Namikawa et al., 2021), which was compatible with the existence of the same haplotype B on Okushiri Island and the Pacific Ocean side of southern Hokkaido (**Figure 3**). The areas are also characterized by short snow-cover period (Namikawa et al., 2021). These vegetational data might afford circumstantial evidence to support the existence of relict *F. crenata* populations on Okushiri Island. In addition, Takahashi et al. (2022) revealed that the leaf area of *F. crenata* with haplotype B was significantly smaller than that of haplotype A individuals, which might be adapted to the dry winter environment, namely short snow-cover period.

Our cpDNA haplotype data suggested that a primary differentiation between Hokkaido and the Tohoku region. *Fagus crenata* on Okushiri Island was dominated by the Tohoku lineage yet showed partial establishment of the Hokkaido lineage in the southeastern populations. In addition, nuclear SSR showed coancestry lineages of Okushiri Island belong to Hokkaido lineages at *K* = 2 of the STRUCTURE analysis. The pollen immigration occurred at the northernmost *F. crenata* population at least 12 km in distance (Kitamura and Nakanishi, 2021). This suggested that gene flow by pollen might occur between Okushiri Island and Hokkaido, where the nearest distance between the two regions is 16 km.

Organelle DNA, such as cpDNA, is maternally inherited and dispersed only by seeds (Mogensen, 1996). Thus, the spatial structure of cpDNA haplotypes might indicate the maternal genetic lineages. Previous studies of organelle DNA diversity of *F. crenata* revealed single or closely related lineages in the northern distribution range (Koike et al., 1998; Tomaru et al., 1998; Fujii et al., 2002; Okaura and Harada, 2002). We focused on local populations in the northern distribution range in this study and observed two haplotypes which can be distinguished by a single SNP difference. These different haplotypes appear to indicate the existence of at least two ancestral origins in the northern distribution range (Hewitt, 1999). Haplotype B was shared among northwestern Okushiri Island and the majority of the Tohoku region, while haplotype A was shared among southeastern Okushiri Island and Hokkaido. Hence, the northwestern populations on Okushiri Island might be more closely related to those in the Tohoku region in this study, while those in southeastern Okushiri Island are closely related to the populations in Hokkaido. This spatial structuring of cpDNA haplotypes might reflect the different expansion routes by seed into Okushiri Island. Like the former studies which characterized refugia of this species by cpDNA haplotypes (Okaura and Harada, 2002; Katai et al., 2011; Katai et al., 2017), different haplotypes on the island might indicate the existence of refugia.

Ooi (2016) resolved the species distribution history by an extensive pollen record. According to this, the most recent increase of *Fagus* pollen in Hokkaido observed after period 5 ka (thousand years ago), with its dominance expanding northward. This is in concordance of the species increase along the Japan Sea coast of Tohoku reflecting the increase of snowfall in this area. However, during the late-glacial (from 14 ka to 12 ka), *Fagus* pollen occurred in southern Hokkaido (Ooi, 2016), an area that corresponded to populations 5, and 11 to 16, which shared haplotype B. Moreover, Hoshino (2001) revealed that *F. crenata* have occurred continuously on Okushiri Island from 6,650 ± 120 years BP to the present. Being so, the species might have existed prior to the most recent northward increase along the Japan Sea coast. Given that the haplotype B is of an earlier prosperous lineage, this lineage, namely relict of *F. crenata*, has been sustained within Okushiri Island. Then, the other lineage, which should be represented by haplotype A, increased dominance from the relict source on the island (Ooi, 2016) or immigrated from the Japan Sea coast of Hokkaido. Possible seed dispersal agents of *F. crenata* were spotted nutcrackers (*Nucifraga caryocatactes*) (Vander Wall and Balda, 1977; Kobayashi and Watanabe, 2003; Nishi and Bekku, 2015) and jays (*Garrulus glandarius brandtii*) (Johnson and Adkisson, 1985). Nishi and Bekku (2015) estimated that the spotted nutcracker flew 10.5 km for hoarding seeds, and Kitamura and Nakanishi (2021) revealed seed migration of *F. crenata* at least 12 km in distance in Hokkaido. Also, European nutcrackers carry seeds as far as 22 km (Vander Wall and Balda, 1977). Thus, a 16 km-long seed dispersal between Hokkaido and Okushiri Island likely occurs. In addition, the cpDNA haplotype mixture such as populations 18, 20, and 31, might have resulted from recent seed dispersal on the island, given that the haplotype A lineage increased dominance more recently than the haplotype B lineage.

### Demographic history and northern persistence of *F. crenata* predated the LGM

The result of the demographic inference suggested that the scenario of the isolation with admixture and secondary admixture was the most likely to explain the genetic structure of *F. crenata* populations examined in this study (**Figure 5**). The STRUCTURE analysis by nuclear SSR genotype revealed different coancestries between Hokkaido (including Okushiri Island) and the Tohoku region when *K* = 2 (**Figure 5A**), and a distinct difference between Hokkaido and Okushiri Island when *K* = 3 (**Figure 5B**). Moreover, PCoA separated the Tohoku region at the first coordinate and secondary separation between Hokkaido and Okushiri Island (**Figure 6**). Although a clear admixture pattern was not detected in Okushiri Island populations at *K* = 2, this might be due to recent secondary gene flow from both Hokkaido and Tohoku populations.

The highest probability was obtained from Scenario 5 (**Table 2**, **Figure 2**, **Supplementary Figure 2**). Although time scales for the demographic events were inferred in this study, transformation of generation time to year is still challenging in tree species as they are long-lived with overlapping of generations (Tsuda et al., 2015). Therefore, generation time assumption causes uncertainty in the inference. However, ecological information is still useful to consider generation time, and a feasible substitution for the generation time can be the earliest reproductive age of the species in the northern range. There are several observations for the earliest reproductive age of *F. crenata* of the northern distribution. In the Kuromatsunai Depression, naturally dispersed *F. crenata* in the open environment have flowered at 20 and 21 years of age (K. Kitamura and H. Saito, personal observations 2013). An ornamental *F. crenata* tree at Kuromatsunai Depression flowered at 18 years after the juvenile plantation (H. Saito, personal observations, 2013). Provenance tests of *F. crenata* at two nurseries of Tokyo University (Chichibu, Saitama, Japan and Furano, Hokkaido, Japan) revealed that the northern originated *F. crenata* flowered 15 and 17 years after juvenile plantation at the earliest (S. Goto and M. Takahashi, personal observations 2013). On the other hand, the age of reproductive trees at the northernmost population was estimated to be 80 years (Kitamura and Nakanishi, 2021). From these facts, we assumed two different generation time; 20 years at the earliest and 100 years at the fully matured status for *F. crenata* in this study (**Table 3**). Thus, the time scales of t1 (admixture from Pop1 to Pop2), t2 (admixture from Pop3 to Pop2), t3 (admixture to generate Pop2) and t4 (splitting time of Pop1 from Pop3) were inferred 35,600 (95% HPD: 1,316 - 150,000), 87,600 (95% HPD: 19,960 - 174,600), 77,400 (95% HPD: 8,620-170,800) and 157,800 (95% HPD: 68,400-198,200) years BP, respectively, under the assumption of generation time of 20 years. When we assumed 100 years for the generation time, they are 178,000 (95% HPD: 6,580 - 750,000), 438,000 (95% HPD: 99,800 - 873,000), 387,000 (95% HPD: 43,100-854,000) and 789,000 (95% HPD: 342,000-991,000) years BP, respectively. The results suggested the divergence between Pop1 (Hokkaido) and Pop3 (Tohoku) predated the LGM even under the assumption of a short generation time of 20 years and even when we considered the 95% HPDs. The divergence time was much earlier when we assumed 100 years for the generation time. Moreover, the demographic inference suggested that the Okushiri Island population was generated by admixture between Hokkaido and Tohoku populations 77,400 (95% HPD: 8,620-170,800) and 387,000 (95% HPD: 43,100-854,000) years BP, under the assumption of the generation time of 20 and 100 years, respectively. The inferred rc value (0.505) suggested that both parental populations equally contributed to the admixture to Okushiri Island population. Notably, asymmetrical secondary admixtures to Okushiri Island population from both parental populations was inferred in this study. However, as the median value of t2 (4,380), time of the secondary admixture from Tohoku to Okushiri Island populations, was slightly larger than t3 (3,870) and their 95% HPDs were overlapped, it is difficult to discuss the time scale of this secondary admixture in detail. On the other hand, the secondary asymmetrical admixture from Hokkaido to Okushiri Island populations was inferred to be 35,600 (95% HPD: 1,316 - 150,000) years BP, under the assumption of the generation time of 20 years, suggesting recent gene flow to Okushiri Island population from Hokkaido, probably in relation to the demographic episode during the last ice age or the LGM. Although it is difficult to estimate locations where ancestors of Hokkaido and Tohoku populations distributed in the past and the secondary admixture could not be a single event nor at a specific time but rather by continuous gene flow over time, these finding shed a new light on past population demography of the northern populations of *F. crenata*, and these results were different from the previous studies which discussed post-LGM northward expansion to Hokkaido from Tohoku region (Tsukada, 1982; Tomaru et al., 1997; Fujii et al., 2002).

**Table 3:** Demographic parameters of Scenario 5 obtained by DIYABC.

		Generation time	
Parameter	Generations	20 years	100 years
t1	1,780 (95%HPD: 65.8-7,500)	35,600 (95%HPD: 1,316 - 150,000)	178,000 (95%HPD: 6,580 - 750,000)
t2	4,380 (95%HPD: 998 - 8,730)	87,600 (95%HPD: 19,960 - 174,600)	438,000 (95%HPD: 99,800 - 873,000)
t3	3,870 (95%HPD: 431-8,540)	77,400 (95%HPD: 8,620-170,800)	387,000 (95%HPD: 43,100-854,000)
t4	7,890 (95%HPD: 3,420-9,910)	157,800 (95%HPD: 68,400-198,200)	789,000 (95%HPD: 342,000-991,000)

Recent studies of distribution shift of species based on palaeoecological and/or genetic data revealed the existence of cryptic refugia and northern persistence of forest tree species in more northerly latitudes than initially expectations which assumed southern refugia in lower altitude, not only in Europe and North America but also Japan (Petit et al., 2002; McLachlan and Clark, 2004; Tsuda and Ide, 2005; Petit et al., 2008; Provan and Bennett, 2008; Liepelt et al., 2009; Parducci et al., 2012; Tsuda et al., 2015; Tsuda et al., 2016; Tsuda et al., 2017). Bradshaw et al. (2010) reviewed long-term distribution dynamics of 3 *Fagus* species; namely, *F. sylvatica*, *F. grandifolia*, and *F. crenata*, and indicated the recent northward spread from several outlying founder populations as well as the location of glacial refugia (Kito and Takimoto, 1999; McLachlan et al., 2005; Magri et al., 2006; Magri, 2008). This hypothesis of northern persistence of cold-tolerant tree species was well supported by species distribution model (SDM) (Svenning et al., 2008) and a combined analysis of the SDM and genetic based-demographic inference (Tsuda et al., 2015). Indeed, a number of palynological studies suggest that there must have been cryptic refugia on the Japan Sea side of Hokkaido (Nakamura and Tsukada, 1960; Nakamura, 1968; Uemura and Takeda, 1987; Kito, 2015; Ooi, 2016). Actually, Tsuda et al. (2015) showed a high possibility of persistence of *Betula maximowicziana*, a birch species that often distributes in cool temperate forests with *F. crenata*, during the LGM mainly in the western part of Hokkaido as well as the northern part of the Tohoku region. The distribution of *F. crenata* in the Pleistocene interglacial periods expanded further north on the Japan Sea side and east to the Pacific Ocean side of Hokkaido (Yano, 1972). Palynological studies revealed that *F. crenata* existed in Hokkaido (Sakaguchi, 1989; Kito and Takimoto, 1999; Kito and Ohkuro, 2012) and Okushiri Island (Yamanoi, 1992; Hoshino, 2001) before LGM. Therefore, *F. crenata* might have been distributed further north and east than the present geographic margin in Hokkaido before the straits divided the Japanese Archipelago (Ito, 1987; Ooi, 2016). Also, phytogeographical evidence of the disjunctive distribution of plants on Okushiri Island and the eastern part of Hokkaido supported the notion that temperate species were distributed further north and east than the present geographic margin (Tatewaki, 1954). Moreover, the Okushiri Strait separated Okushiri Island from Hokkaido prior to the Middle Pleistocene, and then the Tsugaru Strait divided Hokkaido from Honshu in the Riss-Würm interglacial period (Ohshima, 1980). These straits could be indeed physical barriers to prevent long-distance gene flow between regions. Thus, it appears that Okushiri Island, besides the Japan Sea coast of Hokkaido, was a possible persistence of *F. crenata* in the northern range. The similar amount of genetic diversity in the northern populations from Tohoku to Hokkaido regions might be mirrored by long-term persistence of populations as well as no experience of severe bottlenecks by the post-LGM colonization. Indeed, we did not detect any recent bottlenecks in the examined populations in the pilot run of the ABC inference in this study (data not shown), while newly founded northern marginal populations showed the effects of genetic drift (Kobayashi et al., 2013; Kitamura et al., 2015; Kitamura and Nakanishi, 2021). Moreover, *F. crenata* on Okushiri Island showed a comparative number of private alleles of SSR to Hokkaido, which indicated the existence of relict on this island.

Finally, the results of this study coincided with palaeoecological and vegetation studies as well as geology of the Okushiri Island, and supported the existence of possible persistence in the northern distribution of *F. crenata* with secondary admixture from the both parental populations in Hokkaido and Tohoku region. This means Okushiri Island included cryptic refugia of *F. crenata* which persisted for a longer period than expected from the post-LGM expansion.

## Data availability statement

The raw data supporting the conclusions of this article will be made available by the authors, without undue reservation.

## Author contributions

Conceptualization, KK and YT. Methodology, KK and YT. Formal analysis, KK and YT. Investigation KK, KN, TM, and MK. Writing – original draft preparation, KK. Writing- review and editing, KK, KN, YT, TM, and MK. All authors contributed to the article and approved the submitted version.

## Funding

This study was supported by JSPS KAKENHI (JP17K07852 and JP20K06152) and Core-to-Core Program (Asia-Africa Science Platforms: JPJSCCB20220007) from the Japan Society for the Promotion of Science and the 27th Pro Natura Fund Grant Program from the Pro Natura Foundation Japan.

## Acknowledgments

We thank M. Ooue, M. Takahashi, H. Saitou, T. Nagamitsu, A. Nakanishi, and A. Takazawa for their assistance in the field and laboratory work.

## Conflict of interest

The authors declare that the research was conducted in the absence of any commercial or financial relationships that could be construed as a potential conflict of interest.

## Publisher’s note

All claims expressed in this article are solely those of the authors and do not necessarily represent those of their affiliated organizations, or those of the publisher, the editors and the reviewers. Any product that may be evaluated in this article, or claim that may be made by its manufacturer, is not guaranteed or endorsed by the publisher.
