# Supplementary material for: Possible northern persistence of Siebold’s beech, Fagus crenata, at its northernmost distribution limit on an island in Japan Sea: Okushiri Island, Hokkaido
Source: Front Plant Sci. 2022 Dec 15;13:990927. doi: 10.3389/fpls.2022.990927 (PMC9797532; doi:10.3389/fpls.2022.990927)
Supplement: Supplementary file 1 [file DataSheet_1.pdf]

Supplementary Table 1. Study sites of *Fagus crenata* populations.

Site ID	Region	Locality	Longitude East (°)	Latitude North (°)	Altitude (m)	Number of individual s analyzed for SSR	Number of individual s analyzed for cpDNA	Ratio of cpDNA haplotype A	Ratio of cpDNA haplotype B	H_E	Allelic richness	F_{IS} [a)]
1	Hokkaido	Utasai, Kuromatsunai	140.3286	42.6519	88	48	4	1	--	0.771	8.01	0.022
2		Mt. Kuromatsunai, Kuromatsuna	140.2579	42.6118	324	48	4	1	--	0.760	7.99	0.005
3		Garo Plateau, Shimamaki	139.9920	42.5983	510	48	4	1	--	0.766	8.00	0.002
4		Kitahiyama, Setana	139.9913	42.5240	320	24	4	1	--	0.732	7.54	-0.022
5		Kaitorima, Taisei, Setana	139.9315	42.1958	247	47	26	0.77	0.23	0.762	7.64	-0.007
6		Otobe	140.2069	42.0128	199	48	4	1	--	0.751	7.68	0.002
7		Tsubota, Assabu	140.2912	41.8360	312	42	4	1	--	0.776	7.84	0.021
8		Miyakoshi, Kaminokuni	140.1620	41.7200	325	47	4	1	--	0.792	7.91	0.043
9		Chirichiri, Matsumae	140.3101	41.6439	191	48	4	1	--	0.784	7.93	0.053 *
10		Maruyama, Shiriuchi	140.2781	41.4910	196	20	4	1	--	0.785	7.94	-0.014
11		Shirotai, Nanae	140.6985	41.9381	447	46	4	1	--	0.759	7.46	-0.026
12		Ofuna, Minamikayabe	140.9150	41.9325	137	16	4	--	1	0.766	7.56	0.021
13		Manjojiki, Hakodate	140.8470	41.9375	639	48	4	--	1	0.782	7.95	0.009
14		Ayame Marsh, Hakodate	140.8337	41.9199	826	48	4	--	1	0.746	7.41	-0.013
15		Yokotsudake, Hakodate	140.7392	41.8875	299	48	4	--	1	0.769	7.15	-0.002
16		Esan, Hakodate	141.0164	41.7836	209	11	4	--	1	0.711	7.18	0.058
17	Okushiri Island	Monai	139.4281	42.1175	195	48	4	--	1	0.770	7.87	0.013
18		Aonae River upper reach	139.4593	42.1241	389	48	24	0.88	0.13	0.782	7.60	0.042
19		Aonae River upper reach	139.4710	42.1336	425	48	4	--	1	0.773	8.00	0.006
20		Okushiri	139.5012	42.1798	154	45	24	0.13	0.88	0.767	7.95	-0.007
21		Mt. Katsuma	139.4677	42.1964	370	27	4	--	1	0.735	6.68	0.067
22		Washira Horn	139.4502	42.2071	275	51	4	--	1	0.743	7.37	-0.029
23		Aonae River Forest Reserve	139.4562	42.1424	519	42	4	--	1	0.726	7.07	0.004
24		Nagahama	139.5019	42.1099	141	65	4	1	--	0.778	7.71	0.006
25		Takinoma Horn	139.5193	42.2267	67	58	4	--	1	0.757	7.38	0.036 *
26		Inaho	139.5466	42.2357	80	47	4	--	1	0.769	7.81	-0.008
27		Kochidomari	139.5291	42.2051	295	48	4	--	1	0.756	7.79	-0.047 *
28		Tamashimayama	139.5019	42.2099	238	12	4	--	1	0.736	8.00	0.032
29		Yuhama	139.4359	42.1925	96	15	4	--	1	0.732	6.97	-0.026
30		Tomisato	139.4633	42.0865	74	49	4	1	--	0.794	8.04	0.033
31		Yoneoka	139.4329	42.0739	55	39	20	0.85	0.15	0.753	6.91	0.028
32		Akaishi	139.5121	42.1332	94	24	4	1	--	0.776	7.38	0.034
33		Nagahama	139.5064	42.1230	120	24	4	1	--	0.769	7.74	0.015
34	Tohoku	Ohma, Aomori	140.8268	41.3246	70	32	4	--	1	0.731	7.30	-0.011
35		Fukikoshi-Eboshi, Aomori	141.3175	41.0374	460	48	4	--	1	0.783	7.76	0.054 *
36		Asamushi, Aomori	140.8974	40.8997	140	48	4	--	1	0.792	7.53	0.066
37		Ikarigaseki, Aomori	140.6842	40.4171	551	48	4	--	1	0.779	8.23	-0.028
38		Akka, Iwate	141.5556	40.0338	933	47	4	--	1	0.784	7.78	0.058
39		Hayachine, Iwate	141.5619	39.5625	929	48	4	--	1	0.800	8.32	0.081 *
40		Hayachine, Iwate	141.5309	39.5476	864	48	4	--	1	0.777	7.93	0.012
41		Hayachine, Iwate	141.4597	39.5368	912	48	4	--	1	0.755	7.99	0.001
42		Shirakami, Aomori	140.1636	40.5513	708	49	4	1	--	0.762	7.97	0.051 *
43		Mt. Iwakisan, Aomori	140.2796	40.6614	953	47	4	1	--	0.740	7.06	0.053 *
44		Goshogawara, Aomori	140.5412	40.9400	85	48	16	0.31	0.69	0.752	7.69	0.018

[a)] * 5% significant deviation from 0.
