# Supplementary material for: Possible northern persistence of Siebold’s beech, Fagus crenata, at its northernmost distribution limit on an island in Japan Sea: Okushiri Island, Hokkaido
Source: Front Plant Sci. 2022 Dec 15;13:990927. doi: 10.3389/fpls.2022.990927 (PMC9797532; doi:10.3389/fpls.2022.990927)
Supplement: Supplementary file 2 [file DataSheet_2.pdf]

Supplementary Table 2 Prior distributions of the parameters used in DIYABC.

Parameter	Minimum	Maximum
Effective population size		
N1	10	10000
N2	10	20000
N3	10	10000
Time scale in generations		
t1	10	10000
t2	10	10000
t3	10	10000
t4	10	10000
Admixuture rate		
ra	0.001	0.999
rb	0.001	0.999
rc	0.001	0.999
rd	0.001	0.999
Mutation model		
Mean mutation rate	1×10^{-4}	1×10^{-3}
Individual locus mutation rate	1×10^{-5}	1×10^{-2}
Mean coefficient P	1×10^{-1}	3×10^{-1}
Individual locus coefficient P	1×10^{-2}	9×10^{-1}
Mean SNI rate	1×10^{-8}	1×10^{-4}
Individual locus SNI rate	1×10^{-9}	1×10^{-3}
