# Supplementary material for: Possible northern persistence of Siebold’s beech, Fagus crenata, at its northernmost distribution limit on an island in Japan Sea: Okushiri Island, Hokkaido
Source: Front Plant Sci. 2022 Dec 15;13:990927. doi: 10.3389/fpls.2022.990927 (PMC9797532; doi:10.3389/fpls.2022.990927)
Supplement: Supplementary file 3 [file DataSheet_3.pdf]

Supplementary Table 3. Genotypic matrix of 11 microsatellite loci and cpDNA haplotypes used in this study.

Site ID	FS1-03		FS4-46		mfc2		sfc36		sfc378		sfc7		mfc12		sfc1063		sfc1105		sfc1143		sfc18		cpDNA haplotype
01	89	89	228	280	161	186	110	117	237	245	157	161	302	302	205	213	127	130	111	120	170	170	A
01	91	91	225	231	161	213	106	131	239	239	157	157	302	302	211	211	130	130	124	134	168	172	
01	91	97	237	280	170	186	121	121	243	248	157	157	302	302	205	209	127	127	113	120	170	176	A
01	95	95	231	280	166	180	110	110	239	245	153	157	302	302	211	213	130	130	115	115	168	176	
01	93	95	225	280	170	184	121	121	237	241	153	153	302	302	209	219	127	130	119	120	170	170	A
01	91	91	231	280	166	188	133	137	245	246	153	157	302	302	209	211	127	148	113	128	166	170	
01	91	93	234	240	178	190	106	110	235	249	153	153	302	304	203	209	130	161	113	115	158	168	A
01	91	109	231	240	176	178	102	121	243	257	153	161	302	302	209	213	130	171	113	119	168	186	
01	91	95	231	280	180	184	110	129	239	249	153	153	302	302	207	229	127	131	115	130	170	170	
01	97	99	228	231	184	197	106	129	249	251	153	157	302	302	207	213	130	163	111	128	170	174	
01	91	103	231	240	155	176	119	127	237	247	153	157	302	302	203	203	157	171	113	119	167	168	
01	103	109	231	231	182	211	110	110	239	245	153	153	302	302	201	215	130	159	115	117	170	172	
01	95	103	231	237	174	211	110	133	237	251	145	157	302	302	213	215	130	171	115	136	168	168	
01	95	97	231	240	182	190	106	117	237	239	153	161	302	318	203	209	146	150	111	113	164	166	
01	91	91	231	237	176	192	102	117	231	249	153	153	302	304	203	213	131	157	111	113	168	184	
01	91	91	231	231	211	213	102	133	239	248	153	153	302	302	203	211	133	179	111	113	168	168	
01	91	95	231	280	166	176	115	119	239	243	153	153	302	302	203	203	130	131	113	113	168	170	
01	91	95	231	280	166	176	115	119	239	243	153	153	302	302	203	203	130	131	113	113	168	170	
01	91	97	231	231	176	192	106	131	239	245	153	157	302	302	203	203	148	150	111	111	168	170	
01	95	99	231	231	155	192	110	119	239	239	153	153	302	302	201	223	131	159	111	122	166	194	
01	91	97	231	234	166	170	110	121	241	251	157	157	302	302	203	209	127	130	113	120	166	168	
01	95	107	225	280	182	195	108	117	237	248	157	157	302	302	209	211	131	157	111	120	168	168	
01	95	97	231	280	182	201	110	129	243	245	153	153	302	302	205	211	130	131	111	126	166	168	
01	91	95	231	234	176	182	102	110	243	245	145	157	303	310	205	205	130	157	113	120	158	174	
01	97	97	225	280	201	211	102	129	239	243	153	153	302	304	211	211	130	130	111	115	168	168	
01	91	109	231	280	184	211	102	108	243	250	153	157	302	302	211	216	130	130	111	113	167	168	
01	91	97	228	231	155	176	102	133	245	245	153	153	302	311	211	215	130	130	111	111	167	168	
01	93	109	240	280	178	184	110	117	239	239	153	157	302	302	203	203	130	130	111	111	170	194	
01	89	91	231	231	155	190	102	121	239	246	153	161	302	302	203	216	150	171	111	113	168	170	
01	91	91	231	280	182	197	102	121	243	247	145	157	302	302	209	211	130	131	115	122	168	170	
01	97	103	231	244	170	176	108	131	237	252	153	161	300	302	199	213	133	179	111	115	167	194	
01	97	103	231	244	170	176	108	131	237	252	153	161	300	302	199	213	133	179	111	115	167	194	
01	95	97	231	231	170	184	106	131	237	243	145	161	302	302	209	213	131	133	111	115	168	194	
01	91	95	231	231	182	184	115	117	241	243	153	157	302	304	209	213	133	167	122	124	168	168	
01	99	109	231	280	201	211	102	106	245	249	153	161	302	304	205	211	148	154	113	115	164	194	
01	95	99	231	231	188	211	110	117	245	246	153	157	302	304	209	211	148	148	115	124	170	184	
01	91	91	231	280	184	211	117	119	245	245	153	161	302	302	211	215	131	150	111	122	164	168	
01	91	97	237	280	170	186	121	123	237	248	157	157	302	302	205	209	127	130	120	132	170	176	
01	93	97	237	280	195	197	110	123	229	247	153	157	302	302	215	219	167	171	119	144	174	176	
01	91	103	237	280	155	192	108	110	241	245	153	157	302	302	207	211	130	130	115	119	166	167	
01	97	97	231	231	195	236	102	151	241	249	153	153	302	306	203	219	130	130	119	119	170	194	
01	95	99	231	231	155	192	110	119	239	239	153	153	302	302	201	223	131	159	111	122	166	194	
01	91	97	231	231	155	207	117	129	245	252	153	157	302	302	209	209	130	130	113	120	168	170	
01	95	103	231	280	161	184	108	131	249	252	153	157	302	304	213	213	127	130	113	113	164	166	
01	93	95	231	231	176	182	115	119	241	241	157	161	299	302	199	215	130	159	119	126	168	170	
01	95	107	231	231	184	192	110	119	245	245	153	157	302	302	199	211	130	159	117	128	164	170	
01	95	97	231	280	174	184	117	119	245	250	153	153	302	302	211	217	130	159	113	117	170	170	
01	93	95	231	231	182	182	119	121	237	245	153	153	302	302	203	211	127	130	119	124	168	170	
02	95	97	231	237	182	186	108	121	239	246	153	157	290	302	203	211	131	161	117	117	166	170	
02	93	97	231	234	155	182	119	125	243	253	153	153	302	302	205	213	130	169	111	122	170	170	
02	93	93	237	280	155	199	106	117	241	253	153	153	302	302	199	209	127	130	111	117	168	184	A
02	89	93	228	231	182	195	110	113	239	247	153	153	302	302	203	203	127	130	115	117	162	164	
02	93	95	231	231	166	182	102	121	217	255	153	157	302	302	203	211	130	179	111	130	170	172	
02	95	107	231	280	172	182	110	113	241	241	153	157	302	304	203	203	130	148	126	128	168	170	A
02	95	97	231	237	174	192	113	117	245	245	153	153	302	302	199	219	130	148	111	128	168	168	
02	95	95	231	231	195	209	108	129	241	245	153	153	302	302	203	211	130	131	120	126	162	170	
02	91	97	228	231	174	192	115	117	246	252	153	153	302	302	215	215	130	181	111	113	168	170	A
02	93	95	231	237	155	180	110	113	241	249	153	153	302	302	209	211	130	130	120	126	162	172	
02	91	97	231	280	182	192	110	113	237	245	157	157	302	302	203	203	130	159	115	117	164	190	
02	87	95	231	280	182	203	108	133	235	247	153	153	299	302	203	209	127	130	124	126	166	168	
02	87	117	231	231	211	213	121	121	242	247	153	157	302	302	209	215	130	130	113	120	167	172	
02	91	95	225	280	155	190	110	121	241	245	153	153	302	302	209	221	130	159	111	115	168	170	
02	95	95	240	280	182	195	108	131	241	253	153	153	302	306	207	209	130	159	111	111	168	168	
02	91	93	231	231	166	209	110	110	241	247	153	157	302	302	209	211	130	130	111	113	166	170	
02	93	93	231	237	195	195	119	121	237	245	153	153	302	302	209	213	130	161	113	124	166	184	
02	91	95	231	231	166	182	121	127	235	253	153	157	302	304	203	211	131	171	113	119	168	170	
02	89	93	231	280	182	201	102	102	243	254	153	157	302	302	197	215	130	171	117	124	168	168	A
02	93	95	231	231	184	201	117	117	243	245	153	161	302	302	199	211	131	131	117	120	164	166	
02	91	93	231	240	155	192	108	117	237	241	153	157	302	319	203	205	130	163	113	117	164	168	
02	95	99	231	280	174	199	102	108	241	247	153	153	299	302	213	213	130	161	115	132	166	170	
02	93	99	231	234	161	178	108	108	248	249	153	153	302	302	209	213	127	146	124	132	168	170	
02	93	97	228	228	180	182	121	121	245	248	153	153	302	302	205	211	130	163	111	117	166	168	
02	91	97	222	280	168	168	108	110	239	253	145	153	302	306	203	209	130	130	122	122	168	170	
02	91	93	231	231	166	182	110	117	241	247	153	153	302	304	199	205	130	179	111	120	168	170	
02	89	97	234	237	186	192	113	117	241	245	153	157	299	302	209	211	130	130	111	117	166	170	
02	93	97	225	240	188	201	106	110	239	245	153	157	302	302	209	211	129	135	111	128	172	194	
02	91	97	231	231	192	192	110	135	245	250	153	157	302	302	203	211	130	131	111	117	164	170	
02	91	97	237	280	182	201	110	135	239	250	145	153	299	306	207	215	138	150	111	113	170	172	
02	89	89	228	231	166	186	110	121	245	249	153	157	302	302	213	219	130	148	111	132	166	168	
02	97	97	231	231	180	209	102	117	243	247	153	157	302	318	203	216	130	154	124	124	166	168	
02	95	97	231	240	209	213	121	135	246	250	157	157	302	304	203	209	127	130	119	128	168	176	
02	89	91	231	240	166	188	110	117	241	250	153	157	302	302	219	219	130	130	111	122	168	170	
02	91	97	231	240	192	213	108	115	245	250	157	157	302	302	203	216	171	181	117	119	168	170	
02	95	97	231	231	166	213	108	115	217	250	153	157	302	302	203	203	157	181	117	128	170	172	
02	95	103	280	280	182	182	117	139	237	239	153	161	302	302	211	217	130	130	111	111	164	168	
02	93	95	231	280	180	211	121	151	243	245	153	153	302	302	203	209	146	175	111	120	170	170	
02	91	97	231	234	155	155	121	151	235	235	157	161	302	302	211	223	130	130	113	128	166	172	
02	91	95	231	231	182	195	121	129	235	245	153	153	302	302	199	211	148	159	113	128	166	170	
02	93	95	231	231	161	168	110	113	239	239	153	161	302	304	203	215	127	130	120	124	168	170	
02	93	95	231	231	182	195	135	147	239	241	153	153	302	302	211	215	130	159	111	128	168	168	
02	87	95	231	231	166	180	121	129	235	245	153	153	300	302	203	211	127	130	111	126	167	168	
02	89	99	231	231	168	190	121	121	239	239	153	157	300	319	215	223	127	130	111	115	168	168	
02	91	95	231	280	184	215	102	121	245	247	153	153	302	302	205	209	130	157	111	128	168	170	
02	91	95	240	280	155	166	110	110	239	241	153	157	302	302	209	219	148	181	117	122	164	170	
02	91	97	231	231	172	186	108	123	231	249	157	157	302	302	203	203	130	171	111	111	164	170	
02	89	95	280	280	180	182	121	137	239	250	153	161	302	311	203	215	130	130	113	124	164	176	
03	91	93	228	237	197	203	110	113	239	251	153	153	302	302	211	213	130	159	111	113	168	172	A
03	91	91	250	280	155	182	108	117	239	249	153	157	302	302	205	209	130	130	113	124	166	170	
03	91	97	207	280	186	192	108	110	239	241	153	157	315	337	203	213	130	157	124	128	168	170	A
03	91	97	280	280	192	215	117	133	243	250	153	157	302	319	209	211	130	130	124	128	170	170	
03	93	97	231	231	176	182	102	117	247	253	153	157	302	302	215	215	130	130	124	126	168	176	A
03	91	99	231	240	203	262	108	110	223	242	153	157	302	306	207	209	130	130	111	142	168	168	
03	91	93	225	237	170	174	108	110	241	245	153	153	299	300	209	217	130	175	113	113	164	170	A

03	87	93	231	231	176	184	102	102	235	241	153	153	302	304	205	211	130	130	115	120	167	172	
03	93	97	234	237	155	203	117	125	241	245	153	157	302	319	203	213	159	161	130	130	168	170	
03	87	95	231	240	184	192	117	129	246	250	153	157	302	302	199	209	127	130	113	120	164	172	
03	95	97	210	231	176	182	102	117	243	249	153	161	302	302	209	211	130	130	113	124	168	170	
03	93	95	240	280	197	262	117	117	223	235	153	153	304	306	213	215	130	130	111	111	170	170	
03	91	91	225	271	170	195	102	113	229	246	153	157	302	304	211	215	130	175	111	113	168	168	
03	91	95	231	240	166	188	117	131	235	237	153	153	302	302	201	215	130	171	124	128	168	168	
03	95	103	231	231	155	192	102	117	247	249	153	153	301	302	203	217	130	130	115	120	168	168	
03	91	91	231	280	172	199	113	117	247	247	153	161	302	302	209	215	131	163	113	113	168	170	
03	91	91	231	280	172	184	113	129	239	247	153	153	302	302	203	205	131	163	119	120	168	170	
03	91	103	207	237	192	205	110	121	243	247	153	153	306	315	209	215	130	157	111	119	170	172	
03	91	97	228	231	172	176	110	123	231	239	153	157	302	302	213	219	130	130	115	124	170	170	
03	87	95	240	240	184	195	117	127	239	245	153	153	302	302	213	215	127	130	111	120	168	170	
03	91	95	231	231	166	201	110	117	235	246	153	157	300	302	201	203	131	171	120	128	168	170	
03	89	93	231	231	192	195	110	117	239	239	153	153	302	302	203	209	130	130	109	111	168	170	
03	91	97	225	240	170	172	102	108	241	244	153	153	302	318	209	211	157	157	111	120	168	170	
03	91	95	231	244	172	222	117	123	245	245	153	153	302	302	201	209	130	154	111	132	166	168	
03	97	97	237	280	180	195	117	129	237	249	153	161	290	302	203	209	154	157	120	122	168	172	
03	97	97	231	237	178	182	102	108	245	247	153	161	302	302	211	217	131	163	113	113	168	170	
03	93	95	207	280	174	184	102	117	241	241	153	157	302	304	211	211	130	157	105	120	168	170	
03	91	95	228	231	186	211	117	127	249	250	153	157	302	302	209	213	179	179	111	113	168	168	
03	91	95	231	280	172	180	108	110	239	250	153	153	302	337	209	211	130	130	120	128	164	168	
03	91	95	225	231	172	195	108	108	241	244	157	161	302	306	213	217	179	179	120	124	170	172	
03	91	97	231	240	172	182	102	108	249	250	153	161	302	302	203	211	130	131	115	124	168	170	
03	89	95	228	231	174	201	119	121	237	244	153	157	302	302	199	199	127	148	111	124	168	172	
03	93	97	237	280	188	203	108	108	239	241	153	153	302	302	211	213	130	159	113	115	166	168	
03	91	91	231	231	170	184	117	121	241	245	153	153	302	302	209	213	130	130	113	115	168	168	
03	93	99	207	228	166	178	102	110	241	252	153	153	304	304	211	211	130	159	113	117	168	176	
03	93	95	231	231	170	182	108	121	241	245	153	153	299	300	203	209	130	163	113	124	170	170	
03	95	99	225	231	182	182	108	117	237	243	153	157	300	302	199	211	130	130	124	142	170	190	
03	91	95	222	234	186	201	102	129	245	248	153	161	301	302	209	211	130	131	113	120	170	170	
03	91	97	231	231	174	224	125	129	241	245	153	157	302	302	203	211	130	171	115	115	166	170	
03	91	97	231	231	186	203	117	121	241	249	153	153	299	302	211	211	169	179	111	115	166	192	
03	93	93	210	240	190	195	117	117	241	250	153	153	302	302	211	215	130	131	119	122	168	170	
03	91	93	237	237	178	186	102	106	245	248	153	153	302	302	199	211	130	148	117	124	166	190	
03	91	105	231	231	155	195	117	119	245	245	153	153	302	302	209	211	130	130	111	124	168	170	
03	93	95	231	231	178	184	110	117	241	246	153	157	302	304	209	213	130	131	111	113	168	176	
03	95	109	231	231	186	192	110	117	241	250	153	153	302	306	211	215	130	130	111	126	168	170	
03	91	93	207	231	166	197	113	117	237	241	153	157	302	304	213	215	130	154	111	120	172	186	
03	89	95	234	240	180	184	110	110	245	250	153	153	288	302	211	211	130	157	111	124	164	167	
03	95	95	207	280	178	184	113	117	239	241	153	157	299	302	199	219	130	157	111	111	164	170	
04	93	117	231	271	172	195	102	133	247	249	153	157	302	302	207	211	130	133	111	120	166	170	
04	93	99	240	280	172	178	106	108	245	253	153	157	302	304	211	211	130	131	124	126	168	170	A
04	0	0	0	0	0	0	0	0	0	0	0	0	302	302	211	213	130	130	119	126	170	170	
04	91	93	231	280	178	184	102	117	239	241	153	153	299	302	211	211	130	167	120	132	168	168	A
04	93	93	231	237	199	234	117	125	243	249	153	157	302	302	215	215	130	161	111	117	170	170	
04	91	93	231	280	184	186	129	131	235	253	153	157	302	304	211	215	127	130	111	113	168	170	A
04	91	97	231	231	155	192	106	117	247	253	153	157	302	302	203	211	131	148	113	115	168	170	
04	89	91	231	231	195	215	102	131	249	249	153	153	302	302	211	217	130	154	111	115	170	172	
04	91	97	231	231	199	209	102	117	239	243	153	153	302	302	213	215	161	202	113	115	170	170	A
04	93	93	237	237	182	186	102	106	246	246	153	153	302	302	211	215	130	148	111	128	168	190	
04	91	91	237	280	195	215	102	131	249	253	153	157	302	302	203	217	131	161	111	117	168	170	
04	89	89	228	237	182	186	121	137	237	250	153	153	302	302	203	211	130	131	113	113	168	170	
04	95	99	228	231	199	199	110	113	237	249	153	157	302	302	199	203	127	131	124	126	164	168	
04	91	97	234	280	182	184	110	125	245	247	153	157	302	302	207	207	130	130	102	122	166	168	
04	89	93	231	280	155	182	108	121	241	249	153	153	302	302	203	223	130	130	117	117	168	170	
04	89	109	240	280	155	180	108	110	237	246	153	153	299	304	209	215	130	163	113	120	168	170	
04	93	93	231	231	174	182	110	119	239	242	153	153	302	302	203	213	130	148	113	119	168	190	
04	91	93	228	231	184	213	121	131	243	246	153	153	302	302	211	211	130	167	113	113	164	168	
04	89	93	231	231	182	192	106	117	243	245	153	157	302	302	209	215	127	150	115	132	164	170	
04	91	109	231	237	155	213	117	139	243	247	153	153	302	302	209	211	130	154	113	117	164	170	
04	93	97	231	231	163	195	106	110	241	249	153	153	0	0	0	0	0	0	0	0	0	0	
04	93	109	231	234	174	213	110	117	249	251	153	153	0	0	0	0	0	0	0	0	0	0	
04	93	117	228	231	176	201	110	113	239	245	153	161	0	0	0	0	0	0	0	0	0	0	
04	91	93	237	280	155	192	117	121	243	249	153	153	0	0	0	0	0	0	0	0	0	0	
05	91	95	231	280	172	203	108	125	241	247	153	153	302	302	209	211	130	130	111	117	168	170	A
05	93	95	231	237	176	186	106	108	241	243	153	153	301	302	209	219	130	148	113	113	168	170	B
05	93	95	231	237	174	219	102	147	237	245	153	157	302	302	207	221	130	131	111	120	168	170	B
05	91	97	231	231	184	186	110	121	245	248	153	161	302	302	203	219	131	138	111	130	172	174	B
05	91	95	231	280	190	192	102	108	239	245	153	153	302	319	211	211	131	140	111	126	170	176	B
05	93	93	231	231	180	186	108	117	237	239	153	157	302	302	211	211	131	140	120	126	164	170	B
05	89	97	240	240	186	195	108	135	217	245	153	157	299	299	209	211	130	131	126	126	170	170	B
05	89	97	231	231	178	178	102	110	245	245	153	153	302	302	203	211	130	131	111	117	170	176	A
05	89	93	231	280	168	178	113	123	241	243	153	157	302	304	211	213	130	131	111	113	170	170	A
05	91	97	231	231	184	199	108	108	241	247	153	153	302	302	211	211	130	154	113	128	168	172	A
05	87	91	228	280	166	209	102	108	239	252	153	157	302	302	203	209	127	130	122	122	158	172	A
05	95	99	231	231	174	213	110	131	235	248	153	157	302	302	203	215	130	130	111	113	168	168	A
05	91	109	231	237	197	213	119	151	217	245	153	153	302	302	215	215	159	161	120	124	164	170	A
05	91	91	231	231	166	182	106	117	239	253	153	153	299	302	211	215	131	157	111	119	164	170	A
05	91	91	231	231	182	199	106	108	250	253	149	153	302	319	203	211	130	130	102	136	166	168	A
05	91	95	231	231	182	182	108	110	249	250	153	161	302	304	211	215	140	161	117	117	164	168	A
05	91	93	231	280	176	186	102	108	241	245	153	153	302	304	199	203	130	130	122	126	170	186	A
05	97	99	231	231	178	192	108	119	237	245	153	157	301	302	211	211	130	150	105	124	168	170	A
05	93	95	231	231	182	186	108	127	243	245	153	157	301	304	203	209	130	152	102	122	162	170	A
05	91	97	231	231	184	199	102	110	247	249	153	153	319	319	203	203	130	140	117	120	168	170	A
05	91	97	231	231	180	182	108	110	237	241	153	157	301	304	211	211	130	187	111	120	164	168	A
05	95	99	280	280	166	192	110	119	217	249	153	157	302	304	203	211	130	159	119	128	168	170	A
05	89	91	231	234	155	205	110	119	241	245	153	157	302	302	203	211	159	159	119	119	168	168	A
05	91	97	231	280	182	197	110	117	239	252	153	153	304	306	209	213	131	148	111	122	166	170	A
05	91	97	231	280	166	197	108	110	248	252	153	157	302	304	209	209	127	148	111	122	168	168	A
05	89	97	231	231	178	197	110	121	239	252	153	157	302	302	209	213	130	148	122	126	168	194	A
05	91	91	231	240	176	182	102	121	241	249	153	153	302	304	209	215	130	152	120	132	164	166	
05	91	97	231	231	176	182	108	110	241	249	153	153	302	304	209	215	130	152	113	120	164	166	
05	91	93	231	280	166	166	102	110	239	245	153	153	304	304	203	211	131	138	120	122	164	172	
05	93	95	231	231	180	186	110	127	237	246	153	153	302	302	203	203	127	130	111	119	168	168	
05	91	99	231	231	192	215	110	119	235	239	153	157	302	302	211	215	130	130	111	111	168	168	
05	93	97	225	280	155	192	110	117	237	237	153	157	302	302	199	211	127	130	115	130	168	182	
05	93	97	231	240	197	201	110	131	241	247	153	153	299	302	215	221	131	152	111	115	168	170	
05	91	95	231	231	172	197	108	117	239	239	153	153	302	302	207	209	133	148	113	117	168	170	
05	91	97	231	280	182	192	102	121	245	249	153	157	302	302	203	215	130	131	111	111	174	176	
05	89	91	228	231	186	192	108	127	239	249	153	157	302	304	197	211	127	130	120	132	168	170	
05	91	91	231	231	172	178	117	117	243	245	153	153	302	302	203	213	130	161	111	119	170	172	
05	93	95	231	240	195	195	108	115	241	252	153	153	302	319	203	211	130	157	115	119	172	194	
05	93	97	231	280	166	186	108	110	248	267	153	157	300	304	209	211	127	130	111	124	168	170	
05	93	95	231	231	195	213	108	117	237	237	157	161	286	302	209	211	127	130	111	124	168	170	
05	95	97	228	228	201	213	108	110	239	247	157	161	286	302	209	211	130	131	113	115	170	170	

05	89	95	228	231	155	213	108	110	237	239	157	157	304	304	211	213	130	130	113	126	170	170	
05	93	95	225	228	155	188	110	110	245	250	153	157	302	302	203	215	130	131	105	113	166	166	
05	93	99	228	231	170	186	117	121	237	245	153	157	302	304	205	211	131	131	113	126	170	170	
05	97	99	228	231	201	238	110	121	245	249	153	153	302	302	205	211	130	130	113	120	166	170	
05	95	99	231	280	170	178	110	121	245	249	153	157	302	302	205	209	130	130	113	119	168	172	
05	97	109	231	240	178	180	108	108	243	249	153	157	302	304	203	209	131	159	117	126	168	175	
06	91	93	231	280	170	174	110	125	245	245	153	157	302	302	197	213	169	173	130	132	166	170	
06	89	91	231	231	166	184	102	102	247	253	153	153	302	302	203	213	130	135	111	128	170	172	
06	93	95	231	231	166	186	102	123	239	245	157	157	302	302	203	203	130	173	126	132	158	164	
06	91	97	237	280	184	199	108	125	237	249	153	157	302	302	207	209	127	187	105	117	164	170	A
06	89	93	231	280	174	186	102	110	245	247	157	157	302	302	203	213	130	173	111	132	158	170	A
06	87	93	231	237	166	182	110	110	239	241	153	161	302	302	203	213	130	171	113	132	164	170	
06	87	97	231	280	182	211	110	133	239	241	153	157	302	302	203	209	130	159	111	113	170	172	
06	89	93	280	280	184	186	108	110	241	249	153	157	302	302	199	213	130	173	111	134	168	170	
06	89	91	231	231	184	192	102	110	247	249	153	153	302	302	199	213	130	135	113	128	168	170	
06	93	97	231	280	186	190	117	117	239	242	149	153	301	319	209	211	129	159	119	126	168	170	
06	95	97	231	231	182	201	108	127	247	252	153	153	302	302	203	211	159	163	102	117	170	170	
06	95	97	237	280	176	182	110	123	245	248	153	153	302	302	199	203	130	130	113	122	168	170	
06	93	93	234	240	184	190	102	151	237	248	153	153	302	302	207	211	130	130	113	122	170	170	A
06	97	99	231	240	174	186	110	125	245	245	153	157	302	302	203	207	130	131	117	120	166	168	
06	99	109	231	280	166	203	110	110	237	239	157	161	302	302	203	209	130	130	111	126	162	168	
06	95	99	231	231	184	186	110	123	245	245	157	157	302	302	209	211	130	130	120	126	170	172	
06	91	129	231	280	163	182	110	123	237	253	153	153	302	302	203	209	130	130	113	120	168	172	A
06	97	107	231	280	182	184	119	125	239	251	153	153	302	302	213	221	130	159	111	122	170	170	
06	95	99	231	231	182	201	110	123	237	246	153	153	302	302	203	203	130	130	115	120	170	172	
06	89	123	228	240	186	192	108	121	245	245	153	153	315	315	211	215	130	130	119	124	170	170	
06	89	97	231	231	184	192	108	110	235	245	153	153	290	302	209	211	130	159	115	120	167	168	
06	87	97	228	231	176	176	102	108	241	245	153	153	290	302	209	211	159	161	113	120	168	170	
06	89	97	228	231	155	192	108	110	241	245	153	153	319	319	209	211	130	130	120	134	170	172	
06	87	97	231	240	174	192	108	117	241	245	153	153	302	302	205	209	130	159	119	120	168	170	
06	93	95	231	231	182	201	117	121	245	249	153	157	301	302	197	209	130	130	111	120	170	194	
06	91	93	240	280	180	199	110	110	239	248	153	153	302	319	211	211	154	159	119	128	164	168	
06	97	107	231	240	190	205	117	137	237	247	153	157	302	302	203	203	130	179	111	117	170	172	
06	95	99	240	280	163	199	121	137	247	248	153	153	302	306	205	211	129	130	111	120	170	170	
06	95	97	231	240	166	199	110	137	246	247	153	153	302	302	211	215	130	130	111	124	170	170	
06	97	97	231	240	184	199	110	121	235	247	153	161	302	302	203	209	130	130	111	134	170	172	
06	87	97	231	231	155	203	108	113	249	257	157	157	302	302	203	203	129	130	105	115	166	168	
06	91	91	231	280	182	184	102	110	242	252	153	161	302	302	199	213	130	130	115	126	170	170	
06	97	109	231	280	174	176	121	123	239	249	153	153	302	315	203	203	131	154	117	120	170	172	
06	97	99	231	280	166	184	117	127	239	246	153	153	302	302	203	211	127	131	113	120	164	168	
06	91	99	231	280	161	184	102	117	246	252	153	153	302	302	203	209	131	133	113	122	170	170	
06	91	97	228	231	195	266	108	121	241	250	153	161	302	302	209	211	161	175	124	132	164	170	
06	91	93	231	234	161	166	102	110	239	252	153	153	302	315	211	213	133	179	111	119	168	170	
06	87	99	231	231	190	201	110	117	245	251	153	153	286	302	197	219	129	130	113	120	168	170	
06	87	89	231	280	166	182	106	108	245	251	153	153	302	302	197	209	130	179	117	120	168	170	
06	91	93	231	231	182	186	108	110	239	245	157	157	286	302	211	221	129	179	120	120	168	172	
06	91	95	231	231	186	186	110	110	245	249	157	157	301	302	215	221	129	130	111	136	168	172	
06	91	91	231	240	155	170	102	123	241	249	153	153	302	302	213	213	131	150	111	120	168	170	
06	91	91	225	231	163	195	106	117	245	249	157	157	302	304	211	211	131	159	111	115	167	170	
06	91	97	225	231	176	184	102	117	241	245	153	157	302	302	203	209	127	130	117	119	168	170	
06	89	97	225	280	184	195	110	121	239	239	153	161	302	302	209	211	130	179	111	128	166	170	
06	93	109	231	231	166	192	102	102	237	239	153	157	302	310	203	211	130	133	111	122	164	168	
06	91	97	225	237	166	184	110	110	241	247	161	161	302	302	209	213	127	130	132	132	170	176	
06	89	93	225	280	176	201	129	139	237	245	153	157	301	302	209	209	130	130	111	111	168	172	
07	89	109	231	231	186	195	110	121	245	249	153	161	302	304	211	221	127	130	124	124	166	170	A
07	91	97	231	280	170	182	110	110	245	247	153	157	302	304	209	221	130	130	111	122	164	170	A
07	93	97	216	240	176	197	108	110	241	245	153	157	302	304	207	209	130	131	111	126	170	176	A
07	95	109	231	240	203	203	102	106	243	245	153	157	302	302	209	213	130	131	111	111	168	168	A
07	93	95	231	231	176	211	110	117	245	245	153	161	302	302	216	216	130	152	111	113	167	168	
07	91	91	280	280	180	201	110	110	241	245	153	161	302	304	203	213	130	154	113	119	168	170	
07	97	99	231	231	184	186	102	110	241	243	153	161	302	302	209	209	130	130	119	130	164	166	
07	91	91	231	234	155	186	108	110	239	245	153	153	301	304	211	221	131	167	111	113	156	170	
07	91	91	231	231	176	186	102	117	241	242	153	153	302	302	211	213	130	131	109	124	156	166	
07	87	93	231	240	155	201	108	108	239	247	157	157	302	302	211	211	130	146	113	126	166	170	
07	91	123	231	280	166	184	110	121	239	249	153	153	318	318	203	209	131	150	111	130	158	170	
07	89	95	231	231	174	213	133	139	243	253	153	153	302	302	209	211	127	130	120	136	168	170	
07	91	93	228	280	197	199	110	110	243	253	153	153	302	304	207	215	130	130	111	117	168	176	
07	93	93	231	280	155	192	110	117	241	243	153	161	286	301	203	215	130	130	111	113	168	170	
07	95	99	231	240	184	197	102	110	247	253	157	161	302	302	215	215	130	169	111	115	168	170	
07	87	91	231	231	176	186	108	119	241	243	157	161	301	301	211	215	130	130	117	122	170	174	
07	91	91	231	280	174	184	121	137	246	247	153	153	302	306	211	211	127	131	113	130	170	190	
07	93	97	231	231	174	184	117	125	237	247	153	153	302	302	203	213	130	133	122	130	158	164	
07	91	97	280	280	163	192	110	110	248	249	153	154	302	304	209	211	130	152	117	132	164	172	
07	91	97	231	234	182	186	121	121	245	247	153	157	302	302	203	209	130	131	130	142	170	190	
07	93	97	231	280	186	188	117	125	239	241	153	153	302	302	199	203	130	130	122	130	158	174	
07	97	103	234	280	163	168	108	110	241	249	157	157	302	302	209	211	130	152	117	124	168	168	
07	89	95	225	280	174	182	110	151	239	248	149	153	301	301	211	211	130	157	111	120	164	170	
07	93	95	231	231	180	180	102	110	237	245	153	157	304	318	199	209	130	130	117	124	168	170	
07	93	93	231	234	190	192	108	113	239	249	153	157	302	302	211	211	130	131	111	122	168	170	
07	91	93	216	280	176	186	108	110	241	250	153	157	303	318	203	211	127	127	113	119	168	170	
07	93	99	231	231	155	199	102	102	243	247	153	157	302	306	211	211	130	138	113	122	164	164	
07	95	99	210	231	180	190	102	108	239	250	153	157	302	303	203	209	127	130	113	117	170	170	
07	93	95	231	231	186	211	102	113	244	250	153	153	302	304	203	203	130	146	119	122	170	170	
07	93	93	225	231	176	190	102	108	237	241	157	161	302	318	205	211	130	135	111	120	168	174	
07	91	91	231	280	186	195	108	110	239	243	157	161	301	301	209	211	130	148	122	130	164	172	
07	95	97	231	231	166	198	102	113	237	245	153	157	301	301	211	217	130	130	105	124	168	168	
07	91	109	231	280	172	186	108	110	239	247	153	153	302	302	211	223	130	131	111	130	168	172	
07	91	97	234	280	184	184	102	110	240	244	153	153	302	302	209	211	127	130	124	130	170	188	
07	93	97	231	231	184	199	110	123	244	246	153	153	302	318	199	213	130	157	113	124	168	168	
07	91	95	237	280	184	198	102	110	245	246	153	157	302	302	217	217	130	138	111	124	166	168	
07	91	99	231	280	199	203	102	110	246	247	153	161	289	289	213	221	130	130	113	122	162	166	
07	91	93	240	240	155	197	113	151	243	245	153	153	302	304	209	209	130	130	111	113	170	176	
07	91	93	231	240	166	172	110	119	239	247	153	161	304	316	209	209	130	130	111	126	168	168	
07	93	95	228	231	155	201	117	137	237	245	153	153	302	304	209	213	130	130	113	113	166	170	
07	91	97	234	280	184	184	102	110	240	244	153	153	302	302	209	211	127	130	124	130	170	188	
07	91	95	231	231	186	207	110	117	241	245	153	157	302	304	209	217	130	131	111	115	164	168	
08	87	97	228	240	182	195	117	117	245	245	153	153	302	302	213	213	130	135	113	124	166	168	
08	91	97	231	231	166	182	102	102	249	253	153	153	302	319	203	209	130	130	119	142	166	170	
08	91	91	231	231	172	195	110	117	253	253	153	161	302	319	209	215	130	130	119	142	166	172	
08	89	91	231	240	205	213	108	145	239	241	157	157	302	337	203	209	130	131	102	120	168	170	
08	91	91	231	231	166	211	102	102	245	249	157	157	302	337	211	221	130	131	113	113	170	170	
08	91	107	231	231	168	186	102	117	243	245	153	153	302	304	211	213	167	177	113	113	164	170	
08	91	93	225	231	155	184	102	110	246	247	153	157	337	337	209	211	131	154	120	126	168	170	
08	89	97	231	280	161	182	108	123	247	249	153	157	302	302	209	213	133	177	111	120	166	168	
08	95	97	280	280	182	184	110	110	239	241	153	153	302	302	203	205	130	152	113	117	164	170	
08	87	97	231	231	155	176	117	119	243	245	153	153	302	302	199	209	130	130	111	124	164	170	A

08	95	103	228	231	186	201	110	133	248	248	153	153	302	302	213	215	131	161	130	130	166	168	
08	89	97	231	280	166	184	108	117	243	245	153	153	302	302	205	213	130	130	117	119	166	170	A
08	93	95	228	240	192	201	110	127	237	247	153	153	302	302	211	215	150	161	126	130	168	170	A
08	93	107	240	280	155	213	102	106	245	245	153	153	302	319	203	209	130	130	111	113	168	170	
08	91	97	231	231	184	209	117	117	245	249	157	157	302	304	209	213	177	179	113	115	170	170	A
08	91	97	240	280	166	195	110	110	246	249	153	157	302	302	203	215	130	159	119	128	164	170	
08	91	91	231	280	201	201	108	137	237	252	153	157	302	304	209	215	130	130	111	113	168	170	
08	93	97	231	231	174	242	102	110	245	245	153	153	302	302	205	209	152	152	111	120	168	170	
08	89	91	228	280	155	176	110	119	239	246	153	153	302	302	211	215	135	138	111	113	168	170	
08	97	103	240	240	182	184	113	151	241	252	157	161	302	302	211	215	131	173	102	119	166	170	
08	91	93	240	280	176	182	113	129	245	252	153	157	302	302	211	215	130	150	102	117	170	170	
08	91	93	231	231	182	209	102	117	237	243	153	157	302	304	209	211	130	131	120	122	166	166	
08	95	95	231	240	166	176	102	117	239	241	153	157	302	302	211	211	127	167	109	120	168	170	
08	89	91	231	240	176	207	108	110	243	247	157	157	302	302	209	211	138	161	120	144	166	168	
08	91	93	240	280	182	186	102	117	247	249	153	153	302	302	209	211	127	130	102	113	164	168	
08	87	99	231	280	182	192	106	123	245	247	153	157	302	304	213	217	130	187	111	122	168	170	
08	89	91	231	280	166	199	102	121	245	245	153	153	301	304	209	213	163	187	115	120	168	170	
08	97	99	219	240	184	203	110	110	243	245	153	153	302	302	199	215	130	135	111	120	168	170	
08	91	97	231	237	184	197	102	123	243	245	153	153	301	302	209	213	130	131	111	120	170	184	
08	91	93	231	280	174	205	108	110	235	243	153	153	302	304	203	211	130	148	113	113	168	168	
08	89	97	231	231	176	182	108	131	239	245	157	161	296	296	203	209	130	130	124	124	166	168	
08	87	91	231	231	176	178	113	117	243	246	157	157	0	0	0	0	0	0	0	0	0	0	
08	91	93	228	240	166	195	102	108	241	248	153	157	303	303	203	223	127	130	113	120	168	170	
08	91	93	231	237	159	166	108	117	245	245	153	157	303	303	203	207	130	157	113	122	168	176	
08	91	99	231	240	209	215	108	131	239	257	157	161	303	303	209	211	131	133	113	130	168	170	
08	95	99	231	280	155	217	117	119	245	246	153	157	303	303	209	213	130	131	111	126	166	180	
08	95	103	231	234	172	195	102	115	247	249	153	157	303	319	211	217	130	130	117	126	164	178	
08	95	103	231	234	172	195	102	115	247	249	153	157	303	319	211	217	130	130	117	126	164	178	
08	103	103	231	231	166	178	113	115	246	247	153	157	288	303	211	217	130	148	111	117	164	170	
08	91	91	231	240	163	163	123	133	237	245	153	153	303	303	213	217	133	146	111	120	166	170	
08	95	97	225	228	174	174	102	117	249	252	153	157	303	303	209	215	131	154	119	122	168	170	
08	91	91	231	231	176	176	108	133	247	247	153	161	303	303	213	215	130	130	117	120	170	172	
08	91	91	231	231	176	246	108	133	247	247	153	161	303	303	213	215	130	130	117	120	170	172	
08	89	91	240	280	184	190	110	117	245	246	157	161	303	304	199	211	130	146	113	126	170	170	
08	93	93	231	280	155	176	110	137	239	241	157	161	303	303	209	211	154	169	111	122	164	170	
08	91	97	231	231	166	192	110	121	245	247	153	153	302	302	209	211	131	146	111	113	166	170	
08	91	103	231	231	176	182	108	108	241	249	153	153	303	319	211	215	157	187	111	115	168	170	
09	91	93	280	280	155	182	108	119	241	248	153	157	302	302	209	211	130	150	115	120	174	194	
09	87	93	234	280	176	201	119	121	248	249	153	157	302	302	197	217	130	163	111	120	170	194	A
09	91	97	228	280	155	174	102	119	239	252	153	153	302	302	209	211	130	133	111	128	164	166	
09	93	95	240	280	180	184	110	121	239	249	153	161	288	302	197	215	131	133	111	111	166	186	
09	91	97	231	234	170	174	108	117	239	253	153	157	299	304	197	209	127	130	113	115	172	174	A
09	91	93	280	280	192	195	102	117	243	249	153	153	302	302	203	215	130	138	113	128	172	186	A
09	93	99	231	234	172	188	106	110	243	252	153	161	302	302	203	211	130	167	126	128	164	164	A
09	91	109	228	228	159	190	108	110	237	249	153	153	302	302	209	211	130	169	111	111	168	168	
09	91	99	231	280	182	192	110	131	239	241	153	157	302	302	209	213	154	169	111	128	192	194	
09	91	93	231	280	190	203	102	106	245	249	153	153	302	302	201	203	130	154	113	126	168	168	
09	91	97	231	231	155	166	108	133	241	248	153	157	302	337	203	211	130	130	120	122	164	168	
09	91	93	231	231	174	195	108	108	241	241	157	157	302	306	209	215	129	161	111	128	170	194	
09	91	91	231	237	166	186	102	110	239	239	153	153	0	0	0	0	0	0	0	0	0	0	
09	93	95	237	280	184	195	102	117	249	249	153	153	302	302	215	219	130	165	111	113	170	186	
09	95	95	228	237	182	184	102	102	241	241	153	153	302	302	209	215	131	138	126	128	166	186	
09	91	91	225	237	170	182	102	110	239	249	153	153	302	302	203	223	130	165	128	130	168	172	
09	91	91	237	280	195	211	102	110	247	249	153	161	302	306	213	215	130	138	120	128	168	170	
09	95	95	234	237	180	182	110	119	237	249	153	153	302	302	213	223	130	138	113	128	168	168	
09	95	97	231	231	166	166	110	110	237	257	145	153	302	304	203	219	127	129	115	124	168	170	
09	91	97	231	234	157	188	110	110	243	249	153	157	302	302	211	213	127	167	111	120	168	172	
09	91	91	237	237	186	195	110	119	239	241	153	153	302	302	211	223	131	138	111	128	168	170	
09	97	99	225	240	166	197	117	117	239	239	153	157	302	302	199	213	130	131	117	119	166	170	
09	91	93	222	240	155	186	108	117	241	247	153	153	302	319	199	217	130	131	111	117	170	172	
09	91	97	231	280	166	192	117	123	239	245	153	153	302	302	209	211	130	130	111	126	170	170	
09	93	97	234	280	163	166	108	127	241	241	153	161	302	306	203	209	127	130	113	152	166	166	
09	91	103	228	231	163	184	102	110	237	237	153	153	302	302	209	209	130	130	111	111	166	170	
09	91	91	210	240	155	213	117	133	237	241	153	153	302	302	209	211	131	138	113	130	170	170	
09	91	95	231	280	184	197	102	108	241	245	153	153	302	302	209	209	130	148	113	115	166	170	
09	93	93	231	231	184	205	110	121	239	245	153	153	300	300	203	213	130	154	117	128	170	176	
09	93	97	231	280	176	184	108	137	241	248	157	157	302	302	199	203	130	130	111	124	166	168	
09	89	95	231	280	161	178	110	117	241	241	153	153	306	315	211	213	129	133	111	111	168	186	
09	95	97	231	280	174	215	117	117	235	247	157	157	302	302	197	213	131	135	120	128	166	180	
09	93	95	280	280	174	180	110	117	241	249	157	157	302	315	203	209	131	135	111	117	166	180	
09	95	103	234	280	170	182	102	108	241	247	153	153	304	304	219	219	130	130	105	111	170	170	
09	95	103	231	231	182	197	102	110	241	245	153	153	302	306	203	209	130	138	119	124	164	170	
09	91	95	280	280	182	219	123	129	241	246	153	157	302	302	209	209	130	154	115	128	166	194	
09	95	103	280	280	170	170	102	137	237	248	157	157	302	304	215	215	130	130	105	111	170	194	
09	91	91	240	280	174	186	108	117	241	246	153	157	301	304	217	219	130	130	117	124	164	194	
09	93	93	240	240	182	217	110	131	239	249	153	157	302	304	203	209	131	154	111	124	168	170	
09	91	99	231	280	172	192	102	117	243	245	153	157	301	306	203	209	130	135	111	120	168	194	
09	91	93	225	231	180	184	108	110	241	245	153	153	301	302	215	219	130	130	115	124	164	176	
09	91	91	210	231	178	184	110	121	246	249	153	157	302	302	209	215	131	154	113	113	166	170	
09	91	93	225	228	180	197	110	135	241	245	153	153	301	302	211	219	131	157	124	126	164	172	
09	95	95	231	237	174	176	108	110	241	249	153	153	302	315	213	215	129	130	111	124	166	186	
09	91	97	231	231	157	166	110	127	237	245	153	153	302	304	211	211	130	130	120	124	172	194	
09	87	91	280	280	190	190	108	108	248	248	161	161	302	302	209	211	152	154	111	120	168	170	
09	91	103	240	280	186	205	108	115	237	237	153	153	302	306	199	209	130	154	111	126	170	170	
09	91	93	237	240	166	197	106	117	249	249	153	157	302	306	209	209	127	163	115	115	168	194	
10	93	95	240	280	157	190	117	121	239	247	153	153	302	302	203	209	130	187	113	128	168	170	A
10	89	95	231	231	176	192	108	131	241	245	153	157	302	302	209	215	130	165	111	128	162	166	A
10	91	93	228	231	182	192	110	117	243	247	153	153	301	302	209	209	130	175	119	126	168	194	A
10	97	107	225	280	184	184	133	151	247	249	153	157	301	302	209	213	131	152	122	126	166	194	A
10	91	107	228	237	184	197	117	133	235	247	153	157	302	302	203	209	167	175	126	126	168	182	
10	87	93	225	231	182	192	117	123	239	249	153	153	302	319	209	209	130	152	111	113	164	168	
10	83	93	225	225	168	192	117	127	249	253	157	157	300	302	199	213	165	175	113	119	168	170	
10	93	93	225	231	184	242	102	133	247	249	153	153	302	302	197	213	133	152	126	128	164	168	
10	91	93	237	237	182	186	110	119	237	241	157	161	301	302	211	213	130	130	113	124	168	168	
10	91	99	231	237	186	188	102	119	237	249	153	157	302	319	203	211	130	130	113	113	164	168	
10	95	97	231	280	170	190	121	151	245	248	153	153	290	306	213	215	130	130	113	120	164	194	
10	89	93	228	237	184	197	108	133	246	250	153	157	302	302	209	213	130	131	117	119	168	184	
10	95	97	231	237	157	182	131	131	237	239	153	161	300	302	209	225	130	131	115	124	172	174	
10	91	93	231	280	155	174	110	127	241	243	157	161	302	302	211	215	129	130	111	119	172	194	
10	91	99	231	280	176	180	110	121	239	240	153	153	302	302	199	213	131	133	111	120	174	194	
10	89	93	231	231	178	192	102	110	243	253	153	153	302	302	209	211	130	131	115	128	168	174	
10	93	93	225	231	155	174	123	127	239	241	153	157	302	302	215	223	130	130	111	120	174	194	
10	93	93	231	231	163	180	110	119	239	242	153	157	302	302	203	213	130	131	111	111	168	168	
10	91	93	225	231	174	176	110	127	241	245	153	153	302	304	211	215	130	167	120	124	168	174	
10	87	93	237	237	163	186	108	108	243	245	157	161	302	302	211	213	130	130	119	119	172	194	
11	91	95	274	280	166	197	108	117	235	237	153	153	302	319	203	203	130	131	113	119	167	168	A

11	91	97	231	231	168	197	117	125	235	241	153	157	302	302	209	219	130	130	113	113	168	174	A
11	91	97	231	280	166	197	119	121	239	249	153	153	302	302	203	203	130	161	113	115	170	174	A
11	97	99	231	280	180	186	106	121	241	247	153	153	302	302	211	213	131	154	122	130	167	170	
11	91	93	231	280	180	186	106	143	241	257	153	157	304	319	209	213	130	154	111	111	167	168	
11	89	91	231	280	155	190	117	121	237	239	153	153	302	302	203	211	130	133	113	113	168	172	
11	91	93	280	280	176	176	108	127	239	241	153	153	302	306	209	213	130	161	119	122	168	172	
11	93	103	228	231	163	180	121	151	246	247	153	153	302	304	211	211	130	133	113	119	166	170	
11	91	93	240	280	180	190	102	121	239	247	153	161	300	304	213	215	130	133	113	113	168	174	
11	91	93	231	280	166	180	102	121	241	247	153	153	302	304	199	211	130	133	113	130	166	166	
11	91	103	231	231	163	180	121	151	239	246	153	153	302	302	201	211	130	133	111	120	166	168	
11	91	93	210	231	166	180	108	108	239	247	153	157	302	304	203	211	133	148	113	128	166	166	
11	91	95	231	231	180	199	121	121	239	246	153	153	302	304	211	213	130	133	113	120	166	168	
11	93	95	231	231	155	207	102	121	239	247	153	153	302	337	213	219	131	133	113	113	166	170	
11	93	99	231	280	180	213	121	137	239	250	153	153	302	304	209	211	161	161	111	122	166	168	
11	91	97	231	280	155	201	106	121	241	247	153	157	302	304	203	213	127	133	113	115	168	172	
11	91	95	280	280	155	155	108	108	239	245	153	153	304	304	211	215	130	133	111	119	168	168	
11	91	99	231	231	166	213	108	117	237	239	153	161	302	302	209	217	130	131	109	122	158	168	
11	91	91	231	240	168	172	102	117	239	246	153	153	302	304	203	211	130	161	109	132	158	170	
11	91	99	231	231	155	172	117	119	239	239	153	161	302	304	201	203	130	130	111	111	158	166	
11	91	97	231	280	174	180	108	110	239	241	153	153	302	304	209	213	133	171	113	113	166	168	
11	97	109	231	231	172	184	102	110	239	239	153	157	302	302	203	213	159	161	109	124	158	170	
11	91	93	231	240	155	180	106	121	235	239	153	153	299	302	211	213	133	175	111	113	166	170	
11	91	107	231	280	174	184	108	119	246	249	153	153	302	306	211	213	130	161	111	115	164	172	
11	91	97	231	280	184	201	106	108	229	239	153	153	302	302	203	213	127	130	111	119	168	172	
11	91	95	231	280	176	178	102	108	239	247	153	153	302	319	203	213	127	130	111	113	164	172	
11	87	91	231	280	176	209	102	106	239	248	153	153	302	306	211	213	127	131	111	122	168	168	A
11	91	93	228	280	176	184	102	106	241	249	153	157	302	306	209	213	154	161	111	122	167	172	
11	91	91	240	280	184	186	102	102	239	241	153	157	302	302	205	213	130	161	111	126	172	186	
11	87	93	228	280	176	180	106	108	239	241	153	153	302	302	211	213	127	154	111	119	167	168	
11	91	91	280	280	174	184	108	108	239	243	153	153	302	302	213	215	127	130	111	119	168	172	
11	95	97	231	240	166	176	102	110	245	248	153	153	306	319	203	209	130	138	111	120	170	172	
11	91	97	231	231	176	184	102	106	241	245	153	153	301	302	211	211	130	148	113	120	166	172	
11	93	97	231	280	157	186	125	125	245	249	153	153	301	304	203	213	130	138	113	115	168	172	
11	95	97	231	244	157	176	102	119	245	252	153	153	302	306	203	203	138	157	111	113	168	168	
11	91	93	231	231	176	211	102	133	245	252	153	153	301	302	209	209	138	175	111	120	166	168	
11	93	97	231	280	176	205	102	108	245	249	153	157	304	306	205	205	138	167	111	120	168	174	
11	91	91	231	237	157	180	110	125	229	237	153	157	301	301	203	213	131	148	113	126	168	168	
11	87	93	225	280	182	199	108	123	246	249	153	153	302	321	207	213	135	154	111	128	168	172	
11	91	93	231	280	176	197	117	125	249	249	157	157	302	321	203	213	130	130	115	119	168	170	
11	93	97	231	280	176	192	131	153	237	246	157	157	302	321	199	213	130	131	120	120	168	168	
11	91	91	280	280	186	205	102	131	249	259	153	157	302	321	203	213	130	130	111	120	168	170	
11	91	95	231	231	176	205	102	131	241	245	153	153	306	306	203	203	148	175	113	130	168	170	
11	91	91	210	231	178	190	108	127	239	251	153	157	302	302	203	203	148	179	111	117	166	170	
11	91	93	280	280	176	176	117	125	235	249	153	153	302	321	203	213	130	130	115	120	166	170	
11	95	97	231	231	176	199	125	151	229	245	153	153	302	306	203	209	130	148	113	122	168	168	
12	93	95	231	280	155	192	121	127	245	251	153	161	302	319	203	203	130	157	111	120	168	168	
12	93	97	280	280	180	192	108	139	245	245	153	153	302	302	211	211	130	130	113	122	168	168	B
12	83	93	216	231	173	184	102	108	237	245	153	157	300	302	209	211	130	131	111	111	166	170	B
12	89	93	231	280	168	211	106	108	239	239	153	153	302	302	209	211	130	130	111	111	166	168	B
12	91	93	228	280	173	180	108	117	237	245	153	157	302	302	211	211	130	152	111	111	164	168	B
12	83	89	210	280	178	192	108	121	239	245	153	153	302	302	213	213	130	130	122	128	168	170	
12	93	103	240	240	186	192	121	129	239	249	153	157	302	302	205	215	131	163	113	126	168	170	
12	89	89	228	234	176	201	108	110	239	245	153	153	302	304	211	229	130	148	111	119	164	176	
12	97	99	228	231	166	168	106	121	239	248	153	157	300	325	209	215	130	148	111	113	168	168	
12	83	91	216	240	172	173	108	117	243	245	157	157	302	302	209	209	130	131	122	126	170	170	
12	91	97	240	280	155	197	108	123	239	253	153	157	301	302	203	205	131	131	113	115	168	170	
12	91	97	231	240	176	186	110	123	245	249	153	161	302	302	203	209	131	179	122	122	168	170	
12	89	93	231	234	192	250	108	121	239	249	157	161	302	302	199	209	130	161	120	122	166	172	
12	91	101	231	280	166	176	110	110	247	247	153	157	302	304	199	203	130	130	111	128	168	170	
12	93	95	237	237	166	180	102	110	246	247	157	161	302	302	211	221	130	130	113	126	164	190	
12	93	103	231	240	186	195	108	117	245	252	153	153	302	302	199	211	130	163	117	117	168	170	
13	91	93	231	280	176	197	113	119	239	245	153	157	303	303	203	203	131	163	113	132	170	174	
13	93	97	234	237	186	192	113	127	247	248	153	153	303	303	211	219	131	167	111	128	170	170	
13	91	93	231	237	155	155	117	137	241	246	153	153	303	305	211	217	130	131	111	128	168	168	B
13	91	93	231	234	192	195	113	151	235	243	153	161	302	304	217	221	130	130	113	122	168	168	
13	93	95	231	237	172	199	102	110	237	246	153	153	302	305	209	219	130	130	117	122	168	170	
13	93	105	228	231	184	192	102	151	235	235	153	157	302	304	211	211	127	131	113	115	170	188	B
13	89	95	231	240	182	182	117	121	239	245	149	157	302	302	209	213	131	154	124	126	168	170	
13	97	109	228	231	195	201	110	117	239	246	153	153	302	302	205	205	130	165	111	126	166	170	
13	93	95	210	231	155	155	106	121	235	239	153	157	302	304	207	209	130	131	115	122	170	170	
13	91	95	225	231	182	205	102	117	237	239	153	153	303	304	209	211	130	154	113	124	168	170	
13	91	99	228	237	188	195	102	129	241	246	153	153	302	302	211	217	130	130	111	113	166	166	
13	93	93	234	280	172	192	108	117	239	241	153	157	302	302	211	217	130	130	117	132	170	170	
13	89	95	231	231	155	184	113	123	229	239	153	161	302	319	199	213	130	130	113	132	164	170	
13	91	95	231	280	182	203	108	117	245	249	153	153	302	302	199	215	130	130	119	122	166	170	
13	93	95	231	280	155	182	108	110	239	243	157	161	302	302	209	215	130	130	113	122	168	168	
13	93	109	225	280	182	195	110	131	239	246	153	157	302	302	209	213	129	130	113	128	166	168	
13	91	93	231	231	166	205	102	113	249	251	153	153	302	302	213	213	133	169	113	126	168	168	
13	87	91	237	280	155	182	102	121	247	247	153	157	302	302	205	211	130	167	111	122	166	168	
13	93	93	231	231	172	215	110	117	245	249	153	157	302	302	211	215	130	130	120	122	168	168	
13	91	93	231	231	166	182	133	145	229	239	153	153	302	302	203	213	130	148	111	115	164	168	
13	93	95	280	280	166	182	135	143	247	249	153	153	301	302	211	211	129	130	111	120	168	170	B
13	91	97	231	231	166	186	102	113	241	249	153	153	302	302	211	211	127	130	113	122	164	170	
13	93	95	231	234	184	190	108	113	239	249	153	157	302	319	199	209	130	130	111	115	166	170	B
13	89	91	228	280	205	224	102	110	241	243	157	161	302	306	203	209	130	130	113	124	170	176	
13	93	95	231	280	176	182	117	123	242	245	149	153	301	302	203	209	130	154	117	124	170	170	
13	93	107	228	240	182	215	121	137	235	249	153	153	304	306	209	211	131	131	113	115	166	170	
13	87	89	231	280	184	224	102	131	241	249	153	161	302	302	209	211	130	152	113	124	168	176	
13	91	97	231	280	182	201	102	121	235	242	149	153	302	304	209	209	131	154	124	130	170	170	
13	93	95	231	280	170	182	121	151	239	246	153	157	302	305	203	215	130	130	124	126	172	172	
13	93	93	234	280	182	192	151	151	239	241	153	157	302	304	203	221	130	130	113	130	168	172	
13	91	93	228	234	192	192	121	151	235	241	153	157	304	304	211	221	127	130	113	113	168	188	
13	95	103	231	231	161	201	117	121	239	243	153	161	302	306	209	209	130	131	111	122	168	170	
13	93	93	210	237	166	172	108	110	241	241	153	157	301	302	211	217	131	169	117	130	170	170	
13	93	95	240	280	188	199	106	121	243	245	153	153	303	303	197	211	130	133	111	122	166	170	
13	91	109	228	280	155	186	102	121	250	251	153	153	302	302	209	213	130	133	111	124	166	168	
13	93	99	231	280	182	186	102	121	247	251	153	157	302	305	209	209	130	133	119	124	166	168	
13	87	91	228	231	172	186	117	139	235	251	153	157	302	304	209	209	131	133	115	130	166	170	
13	97	99	228	231	176	186	108	121	247	251	153	157	303	303	203	219	130	133	111	124	170	176	
13	91	97	240	240	166	182	102	135	239	241	157	157	302	302	203	219	130	130	111	115	170	170	
13	89	103	231	231	166	170	113	131	239	241	153	153	304	306	209	219	130	131	111	111	168	170	
13	89	91	231	231	159	159	102	113	246	249	153	153	302	319	209	215	130	131	111	124	158	170	
13	91	95	231	280	184	186	108	139	235	251	153	157	301	304	203	211	133	154	119	130	170	176	
13	95	107	231	240	155	197	110	110	239	239	153	153	302	302	203	209	130	150	122	126	168	170	
13	91	91	231	231	180	190	110	121	239	245	153	153	0	0	0	0	0	0	0	0	0	0	
13	91	91	228	231	184	188	117	139	237	245	153	153	301	302	203	211	130	133	122	124	166	174	

13	91	91	231	280	184	199	108	113	241	245	153	153	300	302	211	213	130	163	126	126	166	170	
13	91	95	231	280	184	190	108	110	245	249	153	157	300	302	211	211	130	130	119	126	166	170	
13	91	97	231	231	166	195	102	127	241	242	153	153	302	302	207	215	131	159	117	120	158	164	
14	95	99	231	280	186	203	113	121	245	252	153	157	302	310	211	217	130	130	111	120	168	172	
14	91	93	231	231	155	159	108	129	239	245	153	161	302	302	209	215	130	130	111	130	166	168	
14	93	95	231	240	155	229	110	113	245	247	153	157	302	302	199	215	161	171	111	113	164	168	B
14	97	99	231	280	217	229	110	121	243	247	153	153	302	304	203	213	154	171	111	128	166	168	
14	93	103	231	280	163	188	102	123	246	249	153	157	302	302	209	211	131	131	113	128	168	170	
14	93	95	231	231	163	186	108	123	239	249	153	157	302	302	213	215	130	131	111	120	164	172	B
14	89	93	231	231	180	190	102	125	248	252	153	153	302	304	205	215	130	131	115	119	166	170	
14	91	95	237	280	176	197	102	117	239	245	153	153	302	313	211	221	131	161	113	115	164	168	
14	91	91	231	280	174	176	102	117	239	245	153	157	302	313	199	203	130	131	113	119	162	168	B
14	91	93	231	231	176	196	102	131	239	241	153	153	300	302	203	209	130	131	113	115	170	174	B
14	91	99	231	234	155	186	102	102	245	249	153	157	302	337	209	209	130	131	124	126	168	168	
14	89	99	237	280	161	192	102	106	248	249	153	153	302	302	215	217	130	131	113	124	167	168	
14	91	97	237	280	161	211	106	113	231	245	153	161	302	302	209	215	131	157	113	122	167	170	
14	91	91	280	280	155	155	117	151	246	249	153	161	302	302	199	215	130	133	126	128	167	170	
14	93	99	280	280	180	190	110	110	235	241	153	161	302	304	211	217	131	148	111	113	168	168	
14	91	91	231	234	155	199	102	110	237	249	153	161	302	302	199	209	130	161	119	128	162	170	
14	91	95	280	280	211	215	102	110	239	241	157	157	302	302	199	221	131	179	115	128	172	174	
14	89	93	231	280	155	182	110	113	239	246	153	157	302	302	199	209	133	161	111	120	167	170	
14	95	95	231	231	166	190	102	110	244	245	153	161	302	302	211	215	131	148	111	115	164	168	
14	93	95	231	280	155	172	102	110	239	249	153	153	302	302	203	211	130	161	119	120	167	170	
14	93	95	231	231	172	174	102	117	239	243	153	153	302	302	203	215	130	130	119	126	170	170	
14	91	93	225	280	190	190	117	121	239	246	153	157	302	302	211	217	131	131	113	113	168	194	
14	93	99	231	231	176	195	110	117	239	242	153	153	302	302	205	215	131	131	126	130	168	170	
14	91	91	234	280	155	180	110	113	249	249	153	153	302	302	211	215	130	131	128	132	170	170	
14	93	97	231	231	155	190	110	110	245	246	153	157	302	302	209	211	130	131	120	128	166	167	
14	95	99	231	231	174	217	108	117	243	245	153	153	302	302	215	217	130	131	113	126	170	170	
14	97	99	231	234	176	209	102	110	243	249	153	157	302	302	209	215	131	131	113	120	168	168	
14	95	95	231	280	176	178	110	110	249	249	153	153	302	302	209	211	127	133	117	128	167	168	
14	91	95	231	231	186	186	113	117	239	245	153	157	302	304	211	215	130	130	111	113	168	170	
14	93	97	231	237	195	195	102	117	241	242	153	153	302	302	207	217	130	130	111	126	168	170	
14	91	99	231	280	176	188	117	131	239	249	153	153	302	302	211	211	131	165	111	122	168	170	
14	93	97	231	280	178	182	106	151	241	245	153	157	302	302	209	211	127	130	111	126	168	168	
14	99	109	280	280	205	215	121	131	241	247	153	153	302	302	207	213	130	154	113	128	170	170	
14	93	97	225	280	174	197	117	135	243	249	153	153	302	302	213	217	130	130	113	115	170	170	
14	91	93	231	231	184	195	121	121	239	243	157	157	302	302	213	217	130	131	120	126	164	170	
14	91	97	225	234	176	192	117	135	237	245	153	153	302	302	211	215	130	154	115	119	166	194	
14	93	97	231	280	172	192	102	108	245	249	153	153	302	306	203	211	135	161	119	120	168	170	
14	93	97	240	280	180	190	110	119	239	247	153	153	302	302	209	215	131	165	109	119	172	176	
14	91	95	219	280	180	190	110	113	241	247	153	157	302	302	209	211	130	130	111	113	168	170	
14	95	97	234	237	178	182	117	131	241	247	153	161	302	302	211	213	130	130	119	126	168	170	
14	91	91	219	231	174	195	110	121	239	247	153	153	302	302	213	213	130	130	111	111	168	170	
14	95	99	231	231	186	190	117	125	235	247	153	161	302	302	199	209	130	165	120	128	166	168	
14	93	97	231	240	184	195	106	117	237	245	157	157	302	302	209	211	131	154	111	115	172	182	
14	89	97	231	240	176	213	102	119	247	249	153	157	302	304	215	223	130	131	111	111	172	174	
14	93	99	231	231	166	186	113	119	235	243	153	157	302	302	199	217	130	131	111	130	170	170	
14	91	99	225	231	192	197	123	135	241	245	153	161	302	302	211	213	130	130	119	120	166	170	
14	89	99	231	240	155	215	108	108	239	252	153	153	301	302	211	211	130	131	111	113	166	172	
14	91	91	231	231	195	236	121	135	241	245	153	157	302	302	203	213	130	133	111	113	166	166	
15	91	95	240	240	166	176	102	115	239	241	157	161	302	304	203	203	130	133	111	128	168	170	
15	91	103	231	231	155	166	102	121	249	253	153	153	302	304	203	205	131	133	124	124	170	172	B
15	91	99	231	240	166	211	113	119	239	253	157	157	304	306	211	211	130	133	111	124	168	168	
15	91	91	231	280	166	186	108	119	241	253	157	157	304	306	203	211	130	135	126	128	170	172	B
15	91	91	231	240	172	199	110	119	245	253	153	157	301	304	211	211	130	133	122	128	172	174	
15	91	93	240	240	166	172	106	110	239	249	153	153	302	302	203	219	130	131	111	113	170	194	B
15	91	93	231	240	166	186	108	119	239	241	153	157	302	304	203	221	130	133	113	124	164	172	
15	95	97	231	237	176	199	121	131	237	245	153	157	302	302	199	199	131	163	113	120	164	166	
15	91	93	231	231	166	184	102	110	241	249	153	157	302	304	211	221	130	135	120	128	170	172	
15	95	95	222	231	195	197	102	113	239	253	153	157	304	304	201	203	133	161	111	113	168	172	
15	91	91	231	240	172	176	119	121	241	241	153	153	302	304	203	211	129	135	113	128	168	172	
15	95	95	231	240	166	197	102	113	241	254	153	157	304	304	203	207	133	135	128	128	168	172	B
15	91	95	231	231	180	186	121	131	240	245	153	153	302	302	211	211	130	163	111	120	170	172	
15	91	103	231	231	186	190	131	145	245	249	153	157	302	302	211	211	130	163	111	111	164	168	
15	91	91	240	280	180	180	108	139	237	239	153	157	304	337	211	215	130	133	111	113	167	176	
15	91	99	231	231	188	201	113	117	239	243	153	153	304	304	203	211	130	130	111	119	164	172	
15	89	91	231	237	178	178	102	121	235	245	153	153	301	302	209	211	130	148	113	132	168	170	
15	95	101	222	237	199	211	102	110	239	241	153	153	301	302	209	211	130	161	111	132	168	168	
15	91	95	234	240	201	205	110	117	237	254	157	161	299	302	209	215	130	161	111	113	170	170	
15	91	97	231	231	178	192	102	110	237	239	153	153	301	302	203	209	130	130	111	132	164	170	
15	91	91	237	280	155	184	110	121	237	249	153	161	299	302	211	211	130	130	111	113	168	168	
15	93	97	231	280	184	204	110	115	237	249	153	153	302	302	203	211	130	130	111	111	168	170	
15	91	91	237	280	184	211	108	117	245	249	157	161	299	301	199	215	130	130	113	126	168	174	
15	91	93	237	280	176	195	108	117	249	253	153	157	302	304	207	209	130	133	126	128	168	172	
15	91	91	231	237	174	211	108	110	245	251	153	157	302	302	215	215	130	130	113	120	164	168	
15	91	91	231	237	178	196	102	117	245	245	149	153	301	302	203	213	130	130	115	126	170	176	
15	95	97	231	231	182	184	108	110	245	245	157	161	302	319	213	215	130	157	102	124	164	168	
15	91	91	237	280	176	211	110	110	239	249	157	161	301	302	209	215	130	130	124	126	164	168	
15	89	91	231	237	180	190	117	121	245	249	153	157	299	302	203	215	130	130	113	120	164	166	
15	91	91	231	280	172	184	110	117	245	247	153	161	301	302	199	209	130	130	124	128	168	170	
15	91	95	222	240	172	211	102	110	239	245	153	157	302	302	201	209	130	130	113	122	168	174	
15	91	93	237	280	176	211	110	110	239	245	153	161	299	302	199	215	130	130	124	126	168	174	
15	91	91	231	237	184	190	110	110	237	239	153	157	290	301	205	215	130	133	111	111	164	170	
15	91	95	225	240	172	178	102	145	247	253	153	157	302	304	203	215	130	135	111	128	168	168	
15	91	93	240	240	166	182	119	123	241	253	153	153	302	304	203	211	129	135	128	130	170	172	
15	89	95	231	280	184	201	106	117	243	249	149	149	302	302	211	221	130	130	113	120	170	170	
15	89	91	240	280	176	176	108	110	244	249	154	161	301	302	205	209	130	131	124	126	168	170	
15	91	95	231	237	168	211	110	113	245	249	157	157	299	302	209	211	130	130	113	117	168	174	
15	91	95	231	231	190	211	113	119	239	239	153	157	302	319	209	211	130	130	113	117	166	168	
15	91	95	222	240	190	211	110	117	239	249	153	157	299	299	201	209	130	130	113	113	168	174	
15	91	95	231	240	211	211	102	117	245	249	157	157	299	302	209	211	130	130	111	113	168	174	
15	91	99	231	231	166	172	110	115	239	241	153	153	301	302	209	211	130	131	113	120	166	168	
15	99	109	231	231	155	211	102	108	248	249	157	157	302	306	199	211	130	131	111	126	168	168	
15	91	99	231	240	168	211	113	117	239	245	153	157	302	302	209	215	130	130	113	117	168	174	
15	91	99	280	280	184	184	108	110	241	241	153	153	302	304	203	211	130	130	120	122	164	168	
15	89	89	280	280	174	182	102	127	237	239	153	157	302	302	211	211	163	163	111	113	170	176	
15	89	95	231	280	155	176	102	106	239	253	153	157	302	302	199	211	130	163	113	122	168	186	
15	91	99	231	231	168	190	102	117	239	241	153	153	304	306	209	209	130	130	111	130	168	176	
16	91	109	280	280	178	182	110	113	237	242	153	153	302	304	211	211	130	171	120	120	168	172	B
16	91	95	231	231	190	197	110	110	239	245	153	153	302	302	203	213	130	130	124	130	168	186	
16	95	97	231	231	157	178	108	131	241	245	153	153	302	302	209	213	130	131	102	128	164	166	B
16	93	93	231	280	184	205	110	119	241	245	153	153	302	302	211	215	130	131	105	113	170	170	
16	93	97	240	280	182	252	102	110	239	239	153	153	302	302	199	211	130	131	111	119	166	166	B
16	109	111	219	228	174	197	113	117	239	245	157	157	302	302	211	211	130	131	115	124	166	168	
16	91	91	231	240	180	192	117	117	241	245	153	153	302	302	209	215	131	154	115	120	194	194	B

16	91	109	280	280	178	182	110	113	237	242	153	153	302	304	211	211	130	171	120	120	168	172	
16	89	93	231	231	172	186	119	121	235	253	153	153	302	302	209	211	130	131	111	115	167	170	
16	91	99	231	231	176	180	102	110	235	241	153	153	302	302	209	211	130	157	111	113	166	168	
16	93	123	231	280	178	188	110	119	243	253	153	157	302	302	203	213	130	130	113	117	166	168	
17	93	97	231	237	192	213	110	129	245	257	153	157	302	302	211	213	171	171	111	126	170	194	
17	91	91	231	280	192	201	113	117	239	249	153	153	302	302	211	211	127	130	115	124	164	186	
17	87	91	228	231	161	168	110	110	245	252	157	161	301	302	199	213	127	130	111	130	164	168	B
17	91	97	231	231	155	166	117	121	239	245	153	157	302	302	199	203	130	130	113	117	168	174	
17	93	93	231	237	174	192	117	133	239	248	153	157	302	318	205	215	130	131	113	113	167	170	
17	95	97	231	234	174	180	102	121	243	246	153	153	304	304	197	211	130	130	111	126	164	168	
17	91	97	231	231	186	213	117	121	241	247	153	157	302	302	207	211	130	130	120	120	170	172	B
17	93	97	231	280	155	180	102	110	239	247	157	161	304	304	209	209	127	159	111	122	168	170	
17	87	97	228	231	182	195	102	108	247	252	153	157	302	302	209	213	130	131	111	111	158	170	
17	89	93	231	231	180	190	108	110	239	249	145	157	302	302	209	211	129	146	111	111	164	170	
17	0	0	0	0	0	0	0	0	0	0	0	0	301	302	203	209	130	152	111	120	170	172	
17	91	97	231	280	184	186	121	121	239	245	153	153	302	318	211	219	130	148	113	138	170	170	
17	93	95	231	231	174	213	117	121	229	243	153	153	302	302	209	211	127	130	111	122	168	170	B
17	93	101	231	237	166	184	106	121	239	249	157	157	300	302	211	211	130	130	111	126	164	170	
17	95	97	231	280	155	178	129	131	245	246	153	153	302	302	203	223	127	131	111	126	166	168	
17	97	97	231	240	186	186	102	121	243	245	153	157	302	302	211	215	130	130	111	132	164	170	
17	89	99	231	240	155	176	117	117	239	243	153	157	302	304	209	211	130	159	105	113	170	178	
17	91	93	231	231	155	205	108	121	245	249	153	157	302	318	209	213	130	159	124	124	168	170	
17	91	93	231	280	166	205	108	121	239	249	153	153	302	302	203	209	130	159	111	124	166	168	B
17	95	99	231	234	161	190	113	135	245	246	153	157	302	302	203	209	129	130	111	122	168	168	
17	89	93	231	231	155	184	108	110	237	241	157	161	302	304	203	211	130	169	113	128	164	170	
17	95	95	274	280	172	182	106	139	239	241	153	157	302	304	203	211	130	150	111	111	164	170	
17	91	97	231	280	174	211	106	117	239	253	157	157	304	319	209	209	154	159	113	124	168	168	
17	91	103	231	231	173	182	102	151	245	246	153	157	302	302	203	215	148	171	105	111	170	170	
17	93	95	228	231	172	213	121	151	247	250	153	153	319	319	199	209	130	133	120	130	168	170	
17	91	107	231	280	155	155	102	129	239	241	153	153	302	302	203	209	161	171	113	120	170	174	
17	0	0	0	0	0	0	0	0	0	0	0	0	302	302	211	215	130	130	113	119	167	170	
17	91	97	231	231	155	174	129	133	239	245	157	161	302	302	209	211	130	167	113	126	168	170	
17	87	97	234	280	174	192	108	131	247	249	157	157	302	304	209	211	130	171	119	120	166	170	
17	91	95	231	231	166	190	108	117	239	243	153	157	301	302	211	211	130	130	111	111	170	174	
17	89	91	231	280	176	192	102	121	241	248	153	157	302	339	203	211	131	131	111	124	164	168	
17	97	99	210	231	190	192	108	121	239	249	157	157	302	302	203	215	130	131	115	122	164	174	
17	89	99	231	280	172	199	102	108	243	246	153	161	302	302	211	215	130	130	111	119	168	170	
17	93	103	225	280	192	195	108	115	239	245	153	153	290	302	209	213	130	171	124	144	170	170	
17	93	107	231	237	155	184	106	145	239	246	153	153	306	306	205	211	133	138	111	126	164	170	
17	97	97	231	231	155	197	117	121	243	247	153	153	302	304	211	211	130	167	105	113	168	170	
17	91	93	231	231	180	213	110	131	237	252	157	161	302	302	209	211	130	133	119	122	170	170	
17	91	91	231	280	166	192	119	127	239	245	153	153	302	304	203	211	130	130	111	111	166	168	
17	91	93	231	237	186	186	102	102	245	252	153	153	302	302	215	215	127	130	113	115	167	174	
17	95	95	231	240	155	188	102	108	239	247	153	157	319	319	203	213	130	159	111	120	170	170	
17	91	93	231	231	180	213	110	131	237	252	157	161	0	0	0	0	0	0	0	0	0	0	
17	93	99	231	280	188	195	108	108	239	253	153	153	319	319	203	203	131	183	113	117	167	168	
17	0	0	0	0	0	0	0	0	0	0	0	0	302	304	211	215	130	130	111	130	170	172	
17	89	91	231	280	166	190	102	119	247	258	153	153	301	302	203	211	130	135	105	111	168	174	
17	91	103	231	231	192	195	108	121	241	247	153	153	302	302	211	211	131	183	113	117	166	170	
17	97	97	231	237	155	155	119	121	239	245	153	153	302	302	213	221	127	130	111	111	158	170	
17	91	91	225	240	166	184	115	119	239	243	153	157	286	302	203	215	130	150	113	115	170	170	
17	93	93	231	280	174	176	127	143	245	245	161	161	302	302	209	211	131	159	120	122	168	168	
18	89	97	228	231	192	195	102	108	245	246	153	157	296	302	205	205	130	161	113	126	164	170	
18	97	97	231	280	163	166	110	125	239	247	153	161	302	302	207	211	131	135	111	122	164	168	
18	91	95	231	231	182	186	108	123	249	253	153	157	302	304	199	209	157	169	113	126	168	170	A
18	95	99	231	280	182	192	108	123	239	249	157	157	302	304	199	209	138	157	117	126	166	170	
18	95	99	237	237	176	184	110	121	243	245	153	157	302	304	199	205	130	173	117	126	168	168	A
18	93	107	231	244	166	182	106	108	237	246	157	161	302	302	211	223	127	130	117	119	164	168	
18	95	97	231	231	176	186	117	123	235	253	153	153	302	304	199	211	157	161	111	124	166	168	A
18	91	93	228	240	188	192	108	108	249	249	153	153	302	304	209	211	127	138	120	128	164	172	
18	87	91	225	237	184	190	108	117	247	249	157	161	302	319	211	211	130	138	111	126	166	168	A
18	91	93	222	280	174	205	102	110	235	239	153	157	302	302	199	213	130	171	124	128	164	168	
18	89	93	225	237	172	182	108	110	239	249	157	157	302	304	209	211	130	130	111	111	167	168	B
18	91	95	231	237	192	201	117	125	239	245	153	153	302	302	211	211	130	148	111	119	166	170	A
18	97	97	231	280	192	215	108	117	245	249	153	153	302	302	209	213	130	133	111	115	168	170	
18	95	99	237	237	176	184	110	121	243	245	153	157	302	304	199	205	130	173	117	126	168	168	A
18	89	95	231	237	176	184	106	117	245	249	157	157	302	319	211	213	130	159	111	126	164	170	
18	91	91	231	231	170	173	102	110	239	249	153	153	302	302	209	211	127	130	126	128	164	170	A
18	93	95	228	231	182	186	102	108	243	249	153	157	302	304	199	209	130	150	105	130	170	174	
18	91	97	225	231	182	184	121	121	239	253	153	153	319	319	211	217	130	171	120	122	167	170	A
18	91	93	234	237	166	168	117	117	239	245	161	161	302	304	199	209	130	163	111	124	168	170	
18	91	93	280	280	174	192	117	121	239	248	153	153	302	304	211	215	138	187	111	111	170	172	A
18	91	95	231	231	176	180	102	108	239	245	153	161	302	319	211	215	130	130	111	126	166	168	
18	91	91	237	240	176	176	106	137	245	246	153	153	301	301	211	215	138	148	115	126	164	174	B
18	0	0	0	0	0	0	0	0	0	0	0	0	302	302	199	209	130	130	115	126	168	170	
18	91	93	225	231	176	188	129	139	231	247	153	153	302	302	203	203	130	130	113	122	166	170	A
18	91	99	231	231	182	211	102	113	239	243	153	157	301	301	211	211	130	131	113	115	167	170	
18	91	91	231	237	176	178	102	110	243	247	153	153	301	301	207	209	130	154	111	120	168	170	A
18	91	93	228	240	188	192	108	108	249	249	153	153	301	303	209	211	127	138	120	128	164	172	
18	87	95	225	231	178	192	117	121	247	249	153	153	301	301	199	211	130	135	117	120	168	170	A
18	93	99	231	237	170	178	102	110	245	247	153	153	301	303	209	215	130	154	111	128	168	170	
18	91	97	237	280	174	180	117	137	239	245	153	157	302	304	211	213	130	163	111	119	168	170	A
18	89	97	231	280	163	178	106	110	235	247	153	153	302	302	203	211	127	131	111	126	168	170	
18	91	95	225	237	178	180	102	121	245	247	153	153	302	302	203	207	130	130	111	120	167	167	A
18	97	97	228	231	176	180	102	106	243	246	153	153	306	306	211	215	130	131	117	130	164	168	
18	91	97	228	237	182	182	110	129	241	248	153	157	301	301	211	215	131	171	122	126	168	170	A
18	89	97	237	280	182	184	102	102	239	245	153	157	301	301	211	211	129	130	111	132	170	174	
18	89	93	231	280	176	190	106	108	249	249	157	157	301	301	211	211	130	130	126	130	164	168	A
18	93	103	231	231	184	206	102	110	245	245	153	153	300	303	209	215	130	133	111	115	168	184	
18	93	97	225	280	170	170	121	129	235	240	153	153	302	302	201	207	130	171	124	126	164	170	A
18	99	99	231	231	155	155	102	106	239	245	153	161	302	302	199	209	127	127	124	124	164	168	
18	91	93	240	280	188	203	108	139	239	245	153	153	302	302	209	213	127	130	111	128	164	168	A
18	93	101	231	231	155	182	108	108	241	247	153	161	302	302	211	211	130	130	111	111	164	168	
18	95	107	228	231	182	182	102	123	241	253	153	153	302	304	199	221	130	131	115	124	168	170	A
18	91	95	228	231	174	176	102	102	241	243	153	157	302	302	211	215	130	130	111	119	170	194	
18	93	99	228	280	155	201	108	121	239	245	153	153	302	302	213	215	130	130	111	113	164	170	A
18	91	91	231	237	155	182	108	108	235	243	153	153	302	302	207	211	130	130	113	126	164	170	
18	91	93	237	280	182	211	110	110	239	247	157	157	302	302	209	211	130	130	115	126	168	168	B
18	91	91	231	231	182	211	102	102	243	243	153	153	302	302	211	211	130	130	126	128	167	168	
18	95	103	237	280	176	201	110	117	245	245	153	153	302	319	211	211	148	171	105	119	162	170	A
19	89	91	228	231	155	192	110	151	247	249	153	153	302	302	211	215	130	131	122	132	168	170	B
19	91	95	240	240	178	195	108	131	237	245	153	157	302	302	205	215	127	133	111	120	168	170	
19	87	95	237	240	170	192	108	119	231	245	153	161	302	302	209	213	130	150	111	132	168	170	
19	93	143	225	231	176	195	117	129	239	247	153	157	302	304	207	209	130	131	111	124	168	172	
19	91	93	231	240	166	192	108	108	249	253	153	157	304	324	203	229	133	157	111	111	168	170	
19	91	93	231	240	176	190	106	113	235	249	157	161	286	302	203	211	130	130	113	136	168	170	

19	97	107	228	280	155	184	108	129	245	247	153	153	302	302	207	211	130	133	111	124	168	170	B
19	97	97	231	280	176	199	102	125	245	247	153	153	302	302	209	211	130	171	122	126	166	170	
19	91	93	231	280	182	197	106	113	241	241	153	157	302	302	209	211	130	171	119	128	168	170	
19	87	99	231	237	166	186	106	121	239	252	153	153	304	319	209	211	130	173	115	130	174	174	
19	89	91	231	280	178	186	102	110	239	249	153	153	306	319	205	211	130	173	111	128	168	172	
19	91	91	231	231	161	217	106	110	245	249	153	153	302	302	203	209	130	131	105	113	170	170	
19	95	107	225	234	199	199	108	117	237	253	153	161	302	304	211	215	130	171	111	115	168	170	B
19	91	93	231	280	166	186	102	106	247	247	153	157	315	315	199	205	130	179	122	124	166	190	
19	91	91	231	240	163	195	102	117	243	249	153	153	296	302	209	213	131	138	113	128	162	170	
19	91	109	231	237	172	192	115	117	233	247	149	153	302	311	209	213	130	135	113	113	168	168	
19	87	91	231	280	192	201	102	110	239	245	153	157	302	302	209	209	130	131	111	111	170	170	
19	91	97	231	231	176	192	108	115	237	247	153	157	302	302	203	207	130	130	111	113	166	172	
19	91	95	231	240	161	192	117	121	239	249	153	157	302	302	209	211	130	130	115	117	170	170	B
19	91	97	228	280	211	213	102	110	245	247	153	153	302	302	207	211	131	135	111	115	164	170	
19	93	97	231	231	192	192	117	135	233	247	153	153	302	306	211	211	130	154	113	119	164	170	
19	91	95	237	280	195	201	102	137	245	245	153	157	302	302	203	207	130	163	111	113	170	174	
19	97	99	231	234	174	174	106	106	239	239	153	153	301	306	209	213	127	138	124	124	162	170	
19	95	97	237	237	163	195	102	135	245	253	153	157	302	302	207	207	130	163	111	122	170	174	
19	91	97	231	231	163	174	102	108	239	245	153	153	302	302	203	211	131	175	113	115	167	170	
19	91	101	231	237	161	176	106	139	247	249	153	153	302	304	199	199	130	130	105	115	170	170	
19	95	97	231	280	170	190	106	108	248	249	153	153	302	302	211	211	131	154	111	122	170	170	
19	95	105	231	280	174	176	117	123	239	242	153	153	302	302	209	211	130	130	113	124	170	170	
19	95	97	231	231	157	161	110	117	237	239	153	153	304	304	209	215	130	130	115	122	168	172	
19	91	93	231	231	178	199	102	110	239	239	153	157	302	306	205	213	131	173	111	111	168	172	
19	87	103	231	237	186	192	117	139	241	252	153	153	302	302	209	213	130	130	111	111	166	168	
19	89	93	228	231	168	182	106	106	243	245	153	153	300	300	213	215	127	154	115	128	168	170	
19	91	97	231	231	195	203	108	110	245	245	153	157	302	302	201	209	130	130	119	126	166	190	
19	89	95	231	240	186	195	102	108	239	249	153	157	314	319	205	215	130	130	111	122	168	172	
19	93	95	225	280	174	184	117	145	245	245	153	153	302	302	207	211	130	131	111	126	168	168	
19	93	107	234	237	170	197	102	108	239	243	153	157	300	302	207	215	131	161	124	132	167	172	
19	95	103	231	240	182	190	117	129	243	252	153	153	302	302	203	211	130	131	111	115	167	168	
19	89	91	237	240	182	192	110	113	247	253	153	157	302	304	211	211	130	152	111	126	170	186	
19	89	91	228	231	155	192	110	151	247	249	153	153	302	302	211	215	130	131	122	132	168	170	
19	87	95	231	280	174	184	102	108	245	247	153	153	301	302	205	207	130	130	105	113	170	170	
19	95	97	234	280	155	176	102	113	239	245	157	157	302	302	203	211	127	131	113	119	166	182	
19	91	93	231	280	174	184	115	117	235	243	153	153	302	304	203	213	129	130	111	134	170	172	
19	91	91	231	280	155	161	110	123	235	247	157	157	302	302	211	213	130	133	111	111	168	194	
19	97	103	231	237	192	192	108	110	242	243	153	153	300	300	209	211	130	131	111	111	168	194	
19	93	97	231	231	186	192	102	110	237	246	157	157	302	302	207	211	130	165	122	132	166	172	
19	91	107	237	280	186	192	110	110	249	249	153	153	303	304	199	213	130	181	111	132	168	170	
19	87	95	225	280	182	199	102	110	239	245	153	153	302	302	203	211	130	161	126	128	164	172	
19	91	93	231	280	155	184	110	121	239	245	153	157	302	304	211	215	130	131	115	119	168	168	
20	87	91	231	280	192	195	102	110	243	252	153	161	302	302	203	211	130	130	111	113	164	180	
20	89	91	234	240	184	211	131	131	248	248	153	153	0	0	0	0	0	0	0	0	0	0	
20	91	93	231	240	166	197	110	110	235	241	153	157	302	311	205	213	130	131	124	124	167	170	
20	91	99	225	237	176	184	106	127	239	247	153	153	302	302	203	207	130	167	105	117	162	166	
20	95	97	231	240	163	166	102	121	247	249	153	153	302	306	207	211	130	150	113	132	164	180	
20	91	97	231	240	163	192	102	121	229	241	157	157	302	304	203	211	130	175	111	113	168	172	A
20	91	99	231	231	174	192	108	127	243	253	153	153	302	302	203	211	130	171	120	126	168	168	A
20	93	93	231	280	178	192	102	102	239	253	153	153	302	302	203	207	130	130	111	120	168	180	B
20	93	95	231	240	182	203	102	125	237	239	153	157	302	302	199	203	131	157	111	124	164	170	
20	93	95	240	280	159	192	110	110	246	253	153	157	302	302	199	211	130	161	111	111	166	168	B
20	91	99	231	280	168	205	108	113	247	252	153	153	302	302	209	209	130	131	126	128	168	176	
20	91	95	231	231	192	203	102	110	239	247	153	153	302	302	203	211	130	131	111	117	168	170	
20	91	97	231	240	172	192	110	110	245	245	153	153	302	302	207	211	130	159	117	117	168	170	B
20	93	95	231	231	195	203	102	110	239	243	153	157	302	302	209	209	130	131	113	113	170	170	
20	91	93	231	240	182	192	102	110	241	247	153	157	302	302	209	215	146	150	111	124	172	174	B
20	91	95	237	240	186	195	106	117	242	242	153	161	302	306	211	213	130	130	120	124	164	170	B
20	93	93	231	240	201	209	102	102	246	246	153	161	304	306	207	209	127	177	120	122	168	174	B
20	91	93	228	231	176	215	102	102	239	247	153	161	302	302	203	217	167	175	111	113	162	167	B
20	91	93	231	234	182	203	102	110	239	247	153	157	302	302	205	209	157	198	117	124	170	178	B
20	91	97	240	240	174	186	102	108	249	253	153	157	302	302	207	215	130	150	111	132	167	168	
20	91	93	237	240	176	180	102	106	239	247	153	161	304	306	211	213	130	157	113	113	164	168	B
20	91	91	231	231	166	184	102	127	245	245	153	153	302	302	203	209	130	173	124	126	170	194	B
20	91	97	231	234	178	211	108	110	229	246	153	153	302	306	203	209	130	130	111	117	170	180	
20	97	103	231	240	180	195	108	129	249	249	153	153	302	302	203	211	130	171	117	122	164	166	
20	91	97	231	280	192	199	102	123	248	249	153	161	302	302	199	223	130	130	111	120	168	168	B
20	95	97	231	231	163	195	106	151	239	247	157	157	302	302	209	211	127	152	120	124	170	170	B
20	97	103	231	240	170	180	108	133	241	249	153	157	302	302	203	209	130	130	122	122	164	170	B
20	91	97	231	280	192	195	106	125	237	239	153	157	302	319	209	209	127	130	105	120	168	170	
20	91	93	231	231	178	184	102	108	243	249	153	161	299	301	203	211	130	130	113	126	170	180	B
20	93	97	240	280	195	199	110	131	241	249	153	157	302	302	209	209	127	150	120	120	170	170	
20	91	93	231	240	182	211	125	131	237	247	153	157	302	302	199	209	130	157	124	128	164	188	B
20	87	93	231	231	176	178	127	143	229	239	153	161	302	319	207	215	130	169	119	122	170	170	
20	91	97	231	240	176	201	113	117	239	239	153	153	302	304	211	211	130	130	105	111	168	170	B
20	87	91	231	231	163	197	106	151	245	249	145	153	302	302	215	215	130	131	124	124	170	170	
20	91	93	280	280	182	186	110	135	245	252	153	153	302	302	209	215	161	175	122	126	168	174	
20	91	93	231	240	163	174	129	131	241	247	153	157	302	302	209	211	127	150	119	120	168	174	
20	0	0	0	0	0	0	0	0	0	0	0	0	302	304	203	211	130	175	111	113	168	172	A
20	93	93	231	240	182	199	115	125	237	247	153	157	302	302	199	216	130	157	119	128	164	168	
20	89	99	231	231	174	184	108	127	241	243	153	153	302	302	203	211	131	171	126	128	168	170	
20	97	97	240	280	163	205	110	110	235	250	153	153	302	302	203	207	131	159	115	126	170	172	B
20	91	93	231	240	182	211	125	131	237	247	153	157	302	302	199	209	130	157	124	128	164	188	B
20	93	99	231	231	166	182	108	113	241	252	153	157	302	302	205	211	130	157	111	126	167	170	
20	89	97	240	244	166	192	121	121	249	254	153	157	301	304	207	213	130	131	111	113	164	168	B
20	91	93	231	280	192	201	108	117	241	245	153	153	300	306	211	215	148	177	120	132	168	172	B
20	91	93	231	240	176	182	102	108	243	253	153	157	302	302	207	211	130	161	120	126	164	167	B
21	87	105	237	240	192	197	119	121	239	239	153	157	302	302	207	209	130	175	111	124	164	168	
21	91	93	231	280	192	192	102	102	249	252	153	157	300	304	205	205	130	179	111	113	166	174	B
21	97	97	225	280	192	219	129	143	247	247	157	157	0	0	0	0	0	0	0	0	0	0	
21	93	95	231	231	180	219	119	143	247	249	153	161	302	302	209	213	130	130	105	124	164	166	B
21	93	97	231	240	176	192	113	143	239	249	153	157	302	302	211	223	130	154	111	111	168	168	
21	91	91	231	280	182	192	117	129	247	247	153	153	302	304	203	215	157	179	111	113	166	168	B
21	91	97	240	240	163	195	110	117	239	246	157	157	302	302	199	203	138	163	111	126	167	168	
21	91	91	237	237	166	195	117	117	247	249	153	153	302	302	199	223	130	131	111	115	167	167	B
21	95	95	234	280	174	192	102	121	239	248	153	153	302	304	211	219	130	130	113	122	168	170	
21	97	97	240	280	166	192	102	151	239	239	153	157	302	302	211	211	130	131	122	126	166	194	
21	91	91	240	280	192	192	108	143	239	239	153	157	302	302	209	217	129	131	122	132	166	194	
21	91	91	231	237	192	192	110	119	245	245	153	157	302	302	209	211	129	133	124	128	168	172	
21	89	91	231	231	176	192	102	127	243	249	153	153	302	302	203	215	130	179	113	132	168	168	
21	91	97	240	240	163	195	110	117	239	246	157	157	302	302	199	203	138	163	111	126	167	168	
21	91	97	240	240	163	195	110	117	239	246	157	157	302	302	199	203	138	163	111	126	167	168	
21	89	91	225	231	182	195	117	117	239	239	153	153	302	302	199	213	129	159	124	126	170	172	
21	91	97	240	240	163	195	110	117	239	246	157	157	302	302	199	203	138	163	111	126	167	168	
21	91	91	231	271	192	213	102	113	248	248	153	157	302	302	203	219	127	130	113	115	168	168	
21	91	91	237	237	166	195	117	117	247	249	153	153	302	302	199	223	130	131	111	115	167	167	

21	95	95	231	280	155	192	102	133	235	249	153	157	302	302	207	211	157	161	111	113	166	168	
21	93	95	231	280	176	178	117	117	245	248	153	153	302	306	209	211	130	131	105	111	168	168	
21	93	95	231	280	176	178	117	117	245	248	153	153	302	306	209	211	130	131	105	111	168	168	
21	93	95	231	280	176	178	117	117	245	248	153	153	302	306	209	211	130	131	105	111	168	168	
21	91	97	240	240	163	195	110	117	239	246	157	157	302	302	199	203	138	163	111	126	167	168	
21	93	95	231	231	155	195	110	129	239	245	153	153	302	302	209	215	130	133	111	126	168	168	
21	91	97	240	240	163	195	110	117	239	246	157	157	302	302	199	203	138	163	111	126	167	168	
21	95	95	231	280	176	195	102	129	241	245	153	153	302	302	203	209	127	130	111	126	168	176	
22	91	107	228	231	172	176	117	117	247	249	153	157	302	302	203	211	130	131	111	120	167	168	B
22	95	97	240	280	174	192	102	117	247	252	153	157	302	302	209	211	130	163	111	128	172	172	
22	0	0	0	0	0	0	0	0	0	0	0	0	302	302	209	209	127	130	122	126	168	168	
22	91	105	231	237	192	213	102	143	239	245	153	157	302	302	207	211	131	175	111	132	168	168	B
22	95	109	231	240	182	195	108	110	237	241	153	153	302	302	209	211	130	131	117	130	168	170	
22	89	107	228	237	163	188	110	117	247	253	153	153	302	302	199	209	159	167	111	122	167	168	
22	0	0	0	0	0	0	0	0	0	0	0	0	302	302	209	209	130	131	122	126	168	170	B
22	93	95	231	231	192	213	117	119	231	241	153	157	302	302	209	213	130	130	111	113	170	172	B
22	95	95	231	231	192	192	108	117	249	252	153	153	302	302	209	215	130	161	113	126	168	172	
22	91	93	231	240	161	186	117	117	239	243	153	153	286	302	211	211	131	135	122	126	164	168	
22	91	91	237	240	166	182	121	121	245	248	153	157	302	302	199	199	130	163	111	122	164	166	
22	93	95	225	231	155	186	110	117	239	249	153	153	302	337	199	211	130	130	111	119	168	168	
22	89	95	231	231	174	182	106	110	239	243	153	157	302	337	209	211	130	135	111	126	166	170	
22	93	101	231	280	186	199	108	110	243	249	153	157	302	302	211	211	130	130	111	115	170	170	
22	0	0	0	0	0	0	0	0	0	0	0	0	286	303	209	215	130	135	111	111	168	168	
22	0	0	0	0	0	0	0	0	0	0	0	0	302	302	199	211	130	157	115	136	172	194	
22	93	101	231	280	166	197	110	121	241	243	153	157	318	318	203	211	130	204	120	124	170	176	
22	0	0	0	0	0	0	0	0	0	0	0	0	302	302	209	211	150	179	119	126	170	170	
22	0	0	0	0	0	0	0	0	0	0	0	0	302	304	209	211	130	130	111	120	166	172	
22	0	0	0	0	0	0	0	0	0	0	0	0	302	337	199	211	135	135	111	122	168	168	
22	0	0	0	0	0	0	0	0	0	0	0	0	302	302	209	211	150	161	126	128	170	176	
22	0	0	0	0	0	0	0	0	0	0	0	0	302	304	211	223	130	130	115	119	170	170	
22	91	95	231	231	155	174	110	117	239	245	153	157	302	306	209	211	130	130	120	128	166	168	
22	0	0	0	0	0	0	0	0	0	0	0	0	302	302	199	203	130	173	119	124	168	170	
22	93	101	225	240	182	197	110	121	239	239	153	157	302	302	209	209	127	130	111	124	168	172	
22	93	97	231	280	155	166	110	117	239	243	153	161	302	304	211	211	130	133	113	120	168	170	
22	91	95	231	280	174	199	102	106	239	249	153	153	302	302	209	209	130	150	111	119	168	170	
22	87	95	231	234	184	190	106	117	239	241	153	157	302	304	205	211	130	157	111	113	176	194	
22	0	0	0	0	0	0	0	0	0	0	0	0	302	302	211	215	150	161	119	122	170	170	
22	95	95	231	280	188	192	108	117	241	245	153	157	302	302	209	209	130	130	111	124	170	172	
22	93	95	231	240	166	182	102	110	237	239	153	161	302	302	211	211	131	165	111	120	166	176	
22	0	0	0	0	0	0	0	0	0	0	0	0	303	303	211	213	130	130	119	128	166	167	
22	91	95	231	231	188	199	102	110	239	249	153	153	302	306	203	215	130	171	119	122	168	176	
22	91	93	231	280	166	192	108	127	243	249	153	153	302	304	209	211	127	127	126	130	168	172	
22	89	97	231	237	203	205	108	110	239	239	153	157	302	304	211	213	130	159	120	126	168	170	
22	89	93	231	231	178	186	102	117	239	243	153	157	286	304	211	211	130	130	117	122	168	170	
22	91	93	231	231	192	195	117	117	243	247	153	153	302	304	203	213	130	131	124	128	170	186	
22	95	95	231	237	182	192	117	125	239	252	157	157	302	302	211	215	130	131	111	126	170	170	
22	93	101	231	237	190	201	106	117	239	249	153	153	302	302	207	209	130	150	111	128	170	170	
22	93	99	225	231	186	199	117	121	239	250	153	157	306	337	211	211	130	135	122	124	167	168	
22	95	97	225	231	163	213	110	117	245	253	153	153	302	318	209	211	130	131	111	124	166	167	
22	95	95	231	280	180	192	108	133	239	252	153	153	302	302	207	209	127	130	111	126	170	172	
22	91	95	231	280	182	192	113	117	240	248	153	157	302	302	209	211	130	131	111	126	168	170	
22	91	93	231	280	186	205	117	121	239	241	153	161	302	302	211	219	130	130	111	130	168	168	
22	95	95	240	280	186	192	110	117	239	252	153	157	302	302	199	209	130	131	113	126	168	172	
22	91	93	231	240	157	201	110	117	241	247	153	157	302	302	209	209	130	150	119	119	168	170	
22	89	89	231	234	192	203	108	113	239	241	153	153	302	302	199	209	127	127	113	126	167	172	
22	91	95	231	280	172	174	108	131	245	248	153	153	302	302	209	211	130	130	111	113	168	172	
22	95	101	225	280	166	172	108	110	246	248	153	153	302	302	209	211	130	130	126	126	167	172	
22	91	107	225	280	163	176	108	110	239	253	153	161	302	302	209	213	131	167	111	111	164	164	
22	97	97	228	228	163	178	110	117	253	253	153	157	302	302	209	209	130	131	113	122	167	170	
23	91	97	240	280	155	182	108	117	237	249	153	153	302	302	211	211	127	130	113	126	170	170	B
23	95	119	231	234	159	182	102	117	239	247	153	157	302	302	211	213	130	148	111	124	168	168	
23	93	95	237	240	174	184	102	102	247	247	153	153	302	302	201	203	130	154	111	124	166	170	B
23	89	93	225	231	188	192	108	121	243	259	153	153	311	318	197	211	130	130	119	126	166	170	
23	95	97	280	280	184	192	108	143	229	249	153	157	302	302	209	211	130	130	111	115	170	172	B
23	91	119	231	234	159	192	102	117	239	247	153	157	302	302	199	211	127	130	111	126	168	168	
23	91	95	231	280	186	192	108	121	241	249	153	157	302	302	211	211	130	130	113	128	166	172	
23	93	95	231	280	174	174	108	110	241	249	157	157	302	302	203	219	127	130	111	124	170	170	B
23	95	95	231	240	174	192	102	108	239	249	157	157	302	302	211	211	130	130	111	113	166	166	
23	93	97	231	240	174	182	102	121	247	249	157	157	302	302	211	211	130	171	105	144	166	170	
23	95	97	240	240	155	192	108	108	245	251	153	157	302	302	209	211	131	138	111	130	166	166	
23	93	99	237	280	182	205	117	121	239	253	153	157	302	302	211	211	130	130	113	122	170	176	
23	93	95	231	231	176	213	108	110	243	247	153	161	302	302	211	217	130	131	105	122	168	174	
23	91	95	231	280	192	262	102	117	246	246	153	157	302	302	199	211	127	130	126	132	168	170	
23	89	95	280	280	180	180	119	131	237	245	153	153	302	302	209	211	127	130	126	132	168	170	
23	93	107	231	280	155	155	113	129	237	239	153	157	301	301	203	211	130	148	111	113	170	172	
23	91	95	234	280	182	199	117	127	239	249	153	157	302	302	211	211	129	130	113	126	168	172	
23	91	95	231	234	182	213	110	131	243	247	153	153	302	302	211	211	130	130	126	128	168	172	
23	91	95	231	234	182	195	102	131	237	249	153	153	302	302	203	211	130	133	113	126	166	168	
23	95	95	231	240	176	190	102	121	235	247	153	157	302	302	201	203	163	165	111	124	164	170	
23	87	95	240	280	174	190	108	117	239	247	153	153	301	302	203	211	130	154	111	132	168	170	
23	93	93	237	237	174	192	117	133	245	247	153	157	302	302	209	211	130	154	124	126	168	170	
23	95	95	280	280	163	217	117	129	246	247	153	157	302	303	203	211	130	138	124	126	170	172	
23	95	99	228	240	157	174	108	131	239	249	157	161	302	302	203	211	130	130	111	111	166	170	
23	91	97	231	280	155	163	117	129	245	245	153	161	302	302	199	199	127	130	120	120	168	168	
23	95	95	228	240	176	182	110	151	239	246	153	157	300	302	211	215	131	131	117	119	166	168	
23	93	95	225	280	182	192	110	117	247	251	153	153	302	302	199	211	130	130	111	111	170	170	
23	93	95	237	280	192	215	108	117	239	249	157	157	302	302	201	211	130	130	111	120	170	172	
23	95	97	231	280	182	182	102	108	243	247	153	153	302	304	209	209	130	130	111	126	168	170	
23	93	97	234	280	188	188	102	121	239	239	153	153	303	314	209	213	130	130	111	130	168	170	
23	93	93	231	280	170	217	113	135	246	247	153	161	302	302	209	217	130	130	111	115	170	174	
23	93	95	231	280	182	182	108	121	241	241	153	157	302	302	199	211	130	154	113	124	166	168	
23	95	97	228	240	174	195	108	117	239	247	153	157	302	302	203	211	130	138	111	111	166	167	
23	87	97	234	280	182	190	131	145	249	251	153	157	301	302	211	211	130	130	111	132	168	168	
23	91	97	234	240	182	215	121	131	243	247	153	153	302	302	209	211	130	131	105	126	168	172	
23	93	97	237	237	166	192	117	151	239	247	153	157	302	302	201	215	130	154	111	120	170	170	
23	93	95	228	231	188	192	108	131	247	259	153	153	303	318	197	211	130	130	119	126	166	170	
23	97	97	228	274	184	188	108	121	237	245	153	157	302	302	211	223	150	159	111	126	170	170	
23	93	95	231	280	182	213	110	117	247	253	157	161	302	302	211	215	130	130	111	122	164	170	
23	95	107	231	237	188	217	110	131	247	247	153	153	302	302	209	211	130	131	111	126	170	170	
23	95	99	231	231	166	182	121	121	249	249	153	157	302	302	211	215	127	167	117	124	168	170	
23	93	107	231	231	182	188	110	110	247	247	153	157	302	302	209	215	130	131	122	126	170	170	
24	93	93	231	231	180	211	108	119	242	245	153	153	302	304	211	213	130	144	111	115	167	168	
24	93	93	228	231	195	199	119	129	235	245	153	153	302	302	213	229	130	131	111	113	168	170	A
24	97	99	231	240	166	182	117	129	235	247	153	153	302	304	213	215	130	165	111	122	164	168	
24	89	93	231	231	174	188	117	121	239	241	153	153	302	302	203	211	131	133	115	119	170	170	
24	95	97	231	280	197	211	121	129	235	237	157	161	301	304	211	217	130	130	111	132	164	170	

24	93	95	240	240	184	197	110	129	235	250	153	153	301	303	209	217	159	165	111	111	164	194	
24	97	99	240	240	155	166	110	129	246	249	153	153	304	304	213	217	130	165	122	122	158	168	A
24	95	97	231	240	166	184	102	108	246	249	153	153	302	304	201	215	130	130	111	122	164	164	
24	93	95	231	237	188	192	102	133	241	249	153	157	302	318	211	215	130	171	126	132	164	167	
24	91	95	231	231	166	182	117	129	246	249	153	153	302	304	211	215	165	165	111	122	164	168	
24	91	99	231	280	176	190	110	137	245	256	153	157	302	302	215	225	130	131	111	115	168	170	
24	91	95	231	231	184	186	121	129	245	252	153	157	302	302	209	217	130	131	117	122	170	172	A
24	91	103	237	237	155	186	110	117	245	248	149	153	302	318	211	211	130	165	115	120	164	168	A
24	89	91	231	240	166	178	102	121	241	249	153	153	302	302	203	211	165	202	111	111	168	170	
24	95	97	231	280	163	174	121	129	237	256	153	153	302	302	209	211	130	131	132	132	166	168	
24	91	95	231	271	155	166	117	117	239	247	153	161	302	304	209	211	130	165	122	126	168	168	
24	91	93	231	240	166	182	108	110	249	252	153	153	304	304	209	211	135	165	105	122	168	170	
24	91	93	231	240	182	188	110	137	247	249	153	153	302	304	209	211	135	165	113	122	167	170	
24	93	95	231	231	186	195	102	129	235	241	153	157	302	302	213	229	130	130	111	113	168	170	
24	91	93	231	280	176	182	106	108	237	248	153	153	302	304	201	213	131	133	126	132	168	172	
24	91	97	231	231	176	178	108	108	237	245	153	153	302	304	201	211	133	135	132	136	164	168	
24	97	99	231	231	155	163	117	117	237	247	153	153	0	0	0	0	0	0	0	0	0	0	
24	91	103	225	237	178	192	117	133	241	249	153	153	302	318	211	211	171	202	111	132	168	170	
24	89	91	237	240	186	192	117	117	246	248	153	153	302	302	209	211	130	165	111	120	168	168	
24	89	121	234	237	176	178	102	108	239	248	149	157	302	319	213	215	130	130	120	120	164	170	
24	95	95	231	231	182	186	117	137	231	237	153	153	302	302	211	213	135	173	111	113	164	176	
24	91	91	231	280	182	192	110	117	247	247	153	153	302	304	199	211	130	165	113	122	168	176	
24	93	93	240	280	182	184	108	108	237	248	153	157	302	302	211	213	130	131	113	115	164	167	
24	95	103	228	240	176	186	102	117	237	249	149	153	301	304	215	215	127	202	120	128	168	170	
24	91	93	231	231	178	197	106	108	246	253	153	161	302	302	203	211	130	135	105	132	168	186	
24	91	97	228	228	176	199	108	108	239	245	153	153	299	299	209	209	130	130	105	119	164	172	
24	89	91	280	280	155	182	113	121	246	250	153	153	300	302	199	219	129	130	111	126	168	168	
24	91	93	231	280	155	192	102	106	246	250	153	153	302	302	199	209	135	202	111	115	166	168	
24	91	103	231	240	186	186	117	117	248	252	153	157	302	318	209	215	130	202	120	122	168	170	
24	89	95	231	240	184	197	110	127	247	247	153	161	302	302	207	215	131	146	111	119	170	170	
24	91	93	231	231	182	182	102	110	246	257	153	157	299	304	203	211	130	131	117	126	170	170	
24	91	95	231	240	192	197	121	121	239	245	153	153	300	302	213	213	131	131	111	132	166	170	
24	97	97	231	237	184	209	102	135	239	249	153	153	304	306	201	207	130	165	111	113	164	167	
24	93	95	225	280	186	192	108	113	241	247	153	153	302	302	211	213	165	171	111	128	166	170	
24	89	97	228	280	192	195	108	121	239	250	157	161	302	302	209	211	135	157	132	132	170	170	
24	97	97	231	280	166	182	102	106	247	250	153	153	302	302	199	211	133	135	111	134	168	168	
24	91	99	231	231	155	195	110	121	250	252	153	157	302	318	203	211	130	131	111	113	168	170	
24	93	95	231	280	161	184	102	106	241	248	153	153	302	304	209	213	131	135	111	111	170	172	
24	97	97	231	280	166	182	102	106	247	250	153	153	302	302	199	211	133	135	111	134	168	168	
24	89	95	231	237	176	186	102	110	247	249	149	153	319	337	207	215	169	202	120	140	166	168	
24	91	97	228	231	161	188	102	106	239	249	153	157	302	302	209	211	130	173	115	134	166	168	
24	91	93	231	231	166	192	108	121	241	246	153	153	302	303	209	211	159	173	115	117	168	170	
24	91	93	231	240	184	188	102	108	239	252	153	157	302	304	211	211	130	131	117	128	158	170	
24	91	99	280	280	161	188	108	113	237	246	157	157	302	302	211	211	131	148	113	128	168	168	
24	95	103	231	237	176	178	108	117	247	248	149	153	300	318	211	215	165	171	111	126	168	170	
24	91	93	231	231	192	195	108	113	239	241	153	153	302	302	211	213	130	171	122	128	164	170	
24	91	91	240	280	166	213	113	137	245	246	157	157	302	302	203	211	130	130	117	122	167	168	
24	91	97	240	280	161	188	113	113	246	250	153	157	302	304	211	211	130	131	113	128	168	170	
24	93	95	231	240	184	188	102	129	237	239	153	153	302	302	199	211	130	131	113	130	167	167	
24	91	99	225	280	184	186	127	133	237	241	153	153	302	302	211	211	130	130	117	128	164	172	
24	93	99	231	240	155	188	127	137	239	247	153	153	300	302	209	211	131	165	111	113	164	167	
24	91	121	234	280	174	176	108	121	239	241	153	157	302	304	207	213	130	131	111	120	164	174	
24	93	93	240	240	184	188	121	127	239	248	153	153	300	302	209	209	130	131	111	111	167	168	
24	95	95	231	280	166	192	102	108	239	245	153	157	302	302	213	216	127	173	111	111	168	170	
24	91	95	225	231	184	192	108	113	241	242	153	153	302	304	209	211	131	171	105	132	168	170	
24	93	97	225	231	184	184	108	108	241	246	153	153	302	302	199	209	130	135	105	132	168	170	
24	91	97	240	280	184	201	127	137	239	252	153	161	302	302	209	215	129	130	126	128	167	172	
24	93	97	225	231	184	184	108	108	241	246	153	153	302	302	199	209	130	135	105	132	168	170	
24	91	95	231	240	188	211	102	121	239	252	153	153	302	302	199	209	131	131	111	111	158	170	
24	93	99	231	280	161	201	102	106	241	248	153	157	302	304	211	213	129	130	111	128	167	168	
25	89	91	237	240	176	192	108	121	243	245	153	153	286	302	209	211	129	130	111	120	166	170	B
25	91	97	231	280	178	192	102	102	239	245	153	153	302	302	199	203	130	167	111	113	168	176	
25	95	95	231	280	178	207	102	135	239	249	157	157	302	302	211	215	131	171	124	126	168	170	B
25	93	95	231	280	153	178	102	117	239	245	153	153	302	302	199	203	130	130	113	113	166	170	
25	95	95	231	280	157	190	102	102	245	245	153	157	302	302	199	203	127	130	113	115	166	176	B
25	103	109	231	280	176	217	102	108	239	245	153	157	302	302	203	216	130	154	111	126	170	172	B
25	95	99	231	231	166	192	119	125	243	246	153	153	302	302	199	213	127	148	115	124	168	190	
25	95	95	228	280	178	192	102	133	245	245	153	153	302	302	203	211	127	130	113	134	168	170	
25	91	99	231	231	176	184	102	119	239	243	153	157	286	302	203	209	127	130	115	128	167	180	
25	91	95	231	280	186	199	119	135	249	257	153	157	302	303	203	209	130	131	122	126	162	172	
25	91	93	231	237	166	192	102	108	237	246	153	153	0	0	0	0	0	0	0	0	0	0	
25	91	95	231	280	182	184	108	110	239	249	153	157	302	302	203	205	130	131	111	115	168	170	
25	91	93	231	237	184	186	108	108	237	246	153	153	302	304	209	213	130	130	111	111	170	170	
25	93	95	231	240	163	166	102	123	243	246	157	157	318	318	203	215	130	131	113	119	172	172	
25	95	95	231	280	178	192	102	102	246	250	153	153	302	302	199	211	130	130	113	117	166	172	
25	93	95	231	237	166	199	100	143	243	249	153	153	286	304	199	209	130	131	109	111	170	170	
25	91	95	231	231	166	176	102	110	241	249	153	153	302	304	209	209	130	130	113	124	170	170	
25	91	91	231	231	176	199	108	129	247	257	153	153	302	302	205	209	130	133	120	120	166	168	
25	89	93	231	280	163	176	102	143	245	249	153	153	302	306	209	211	130	171	113	113	167	170	
25	87	95	231	240	155	163	110	117	239	257	153	157	300	318	211	215	130	161	119	122	168	172	
25	89	95	231	231	176	178	102	117	239	245	153	153	302	306	203	213	130	131	115	124	168	176	
25	87	95	237	280	163	178	102	137	245	249	153	153	302	302	203	203	130	130	120	120	164	164	
25	93	97	231	280	155	199	102	108	245	245	153	157	302	302	203	215	127	130	120	128	164	172	
25	91	95	231	231	176	199	108	117	237	245	153	153	302	302	209	213	127	130	122	128	168	170	
25	91	97	240	240	155	188	106	110	241	247	153	153	302	302	207	209	131	131	111	124	172	172	
25	93	97	231	280	159	199	102	119	245	245	153	157	302	302	203	203	130	130	115	120	164	170	
25	89	95	231	240	163	190	102	102	242	250	153	157	302	302	199	217	130	130	111	113	166	172	
25	93	95	231	231	176	178	102	110	237	250	153	153	302	303	203	211	130	175	113	115	168	170	
25	91	109	210	240	155	174	102	108	241	249	153	157	302	302	199	209	129	130	111	120	167	167	
25	93	93	231	240	159	174	108	119	239	246	153	157	302	302	213	217	130	130	115	144	168	170	
25	91	93	234	240	184	186	102	119	245	245	157	161	302	302	209	213	130	165	113	126	168	172	
25	95	95	231	231	174	184	117	117	241	249	153	157	302	304	215	217	130	130	113	122	166	167	
25	91	97	231	231	176	182	117	119	237	239	153	157	286	303	209	211	133	150	115	124	167	172	
25	97	97	231	240	166	178	102	119	245	245	153	153	302	302	203	209	130	130	115	124	166	168	
25	91	95	231	280	166	192	102	110	239	246	153	153	302	302	199	211	130	130	111	117	170	172	
25	97	97	231	240	192	199	108	129	239	239	157	157	286	302	203	209	130	130	120	124	167	170	
25	97	109	228	240	163	174	102	110	247	247	153	157	302	318	203	209	130	131	119	119	164	167	
25	95	109	231	280	174	182	110	110	241	245	157	161	302	314	199	215	130	130	111	119	168	170	
25	91	93	240	280	176	176	102	117	237	239	157	157	302	302	211	211	157	163	126	138	170	170	
25	91	93	237	240	182	213	110	119	241	245	153	153	286	286	205	211	131	131	111	124	166	168	
25	91	95	231	231	163	168	117	117	245	247	153	153	302	302	203	209	130	154	111	124	166	168	
25	91	91	231	237	174	184	119	121	241	245	153	153	302	302	209	215	130	165	111	122	168	168	
25	91	93	231	231	184	203	102	121	239	245	153	157	301	302	213	215	130	131	115	122	168	170	
25	93	95	228	280	166	182	108	110	239	239	153	157	302	304	203	215	127	130	111	122	164	168	
25	91	97	231	231	182	186	108	108	235	248	153	153	302	302	211	215	131	138	117	117	164	170	
25	95	95	228	231	182	192	102	110	245	246	153	153	302	302	211	213	130	130	120	124	164	168	

25	91	95	231	231	192	192	102	125	231	246	153	153	302	302	199	211	130	148	117	124	168	172	
25	95	95	231	240	163	197	106	110	245	249	153	153	302	303	215	215	127	130	122	142	166	166	
25	91	93	240	240	182	192	117	135	240	247	153	153	303	306	194	209	131	150	113	126	162	174	
25	97	105	231	280	176	188	106	119	239	246	153	157	304	314	211	211	150	159	111	128	164	170	
25	95	95	231	280	174	178	106	131	239	249	153	157	302	303	203	213	131	131	115	124	168	170	
25	93	93	240	280	182	199	102	102	240	250	157	157	302	302	209	209	130	157	113	126	164	180	
25	95	97	240	280	155	190	102	133	243	245	153	153	302	303	199	209	130	157	113	124	166	167	
25	91	95	240	280	176	184	110	113	246	247	153	157	302	302	207	217	130	148	113	113	170	180	
25	91	91	231	231	186	215	102	119	245	246	153	153	302	303	199	211	130	131	113	126	170	170	
25	93	95	231	240	161	174	102	110	241	247	153	157	289	289	203	203	130	130	111	119	167	167	
25	95	95	231	240	174	192	110	137	243	247	157	157	302	302	203	203	130	133	117	120	164	167	
25	95	95	231	240	159	176	106	113	239	239	153	157	302	302	203	217	130	131	115	144	168	170	
26	95	97	231	240	182	186	110	127	239	241	153	153	302	302	213	215	130	163	115	117	168	168	
26	91	97	240	240	186	192	110	110	243	249	153	153	302	302	199	229	130	135	119	126	168	170	B
26	93	93	228	231	166	213	106	108	245	245	153	161	302	302	209	209	130	157	111	126	164	170	
26	87	97	231	240	163	209	108	110	241	251	153	153	286	302	211	215	130	133	111	122	164	170	
26	91	95	231	280	163	192	108	125	237	247	153	153	318	337	211	217	130	130	111	124	168	168	B
26	93	97	228	231	163	207	121	145	237	239	153	153	302	318	199	203	130	148	117	124	168	168	
26	93	93	210	240	163	166	117	117	239	246	153	153	302	306	203	203	130	130	111	128	170	170	
26	93	93	231	240	166	207	108	145	239	253	157	161	302	302	203	209	130	157	122	124	164	168	
26	93	93	240	240	166	174	108	117	245	247	153	161	302	302	203	209	130	157	106	122	168	176	B
26	87	95	231	237	166	178	110	117	245	248	153	161	302	302	209	211	130	131	117	120	172	174	B
26	91	99	280	280	188	201	121	125	242	247	153	161	302	318	203	211	130	130	111	113	168	168	
26	91	97	231	280	182	192	102	125	242	247	153	153	319	319	203	211	130	130	111	122	168	172	
26	93	93	225	231	155	166	108	131	239	245	153	161	302	302	209	211	130	130	113	122	164	168	
26	91	93	231	240	166	176	123	131	240	247	153	153	0	0	0	0	0	0	0	0	0	0	
26	91	91	225	231	166	166	108	108	235	239	153	161	304	306	203	211	130	130	111	115	168	172	
26	91	93	231	280	176	195	108	117	245	251	153	153	304	337	207	217	130	130	113	122	166	172	
26	87	93	234	240	163	166	108	117	237	245	157	161	302	302	209	211	130	131	120	122	168	170	
26	89	93	280	280	184	192	106	133	243	245	153	153	302	311	203	211	130	130	111	119	170	176	
26	89	91	225	231	174	195	110	113	239	245	153	153	302	302	209	215	135	138	111	124	168	170	
26	91	95	231	240	166	174	108	121	239	243	153	153	302	302	211	213	130	131	111	115	164	170	
26	95	107	231	240	166	184	108	135	237	239	153	157	302	318	203	203	131	153	111	122	168	170	
26	91	97	231	237	182	184	108	110	240	242	153	153	302	319	205	209	130	130	122	132	168	168	
26	91	99	231	280	166	188	102	110	245	246	153	153	302	304	203	211	131	171	111	119	167	176	
26	91	91	240	280	174	188	117	125	247	249	153	153	302	318	209	211	131	135	111	122	168	168	
26	91	95	240	280	163	174	108	110	245	246	153	153	302	306	205	209	130	133	111	120	164	170	
26	89	93	240	280	182	195	102	110	245	247	153	153	302	302	205	209	130	133	111	126	167	170	
26	91	95	240	280	182	188	102	102	245	247	153	153	302	302	205	211	130	133	120	124	170	172	
26	91	95	231	240	182	184	117	127	241	251	153	153	302	304	209	215	157	194	105	124	164	168	
26	91	93	240	280	184	188	102	110	246	247	153	153	302	318	205	211	130	157	111	111	168	170	
26	91	95	210	280	166	174	117	149	237	239	153	161	302	302	203	203	130	192	115	124	168	170	
26	91	93	280	280	172	215	110	139	239	239	153	157	300	302	211	215	135	138	111	119	170	176	
26	91	97	231	231	174	201	110	123	237	249	153	153	302	303	211	219	130	171	113	113	168	170	
26	91	93	228	231	184	199	108	117	235	240	153	153	302	318	211	215	127	130	111	111	166	170	
26	0	0	0	0	0	0	0	0	0	0	0	0	302	318	209	209	154	179	111	111	168	176	
26	97	97	237	280	184	184	108	115	237	245	153	157	302	302	207	211	127	130	111	132	166	170	
26	91	97	240	280	163	186	113	151	243	250	153	153	302	304	205	215	135	159	120	128	166	168	
26	91	95	240	250	176	178	102	151	239	246	157	161	302	337	203	203	130	159	124	126	167	168	
26	93	93	231	280	188	209	117	127	247	249	153	161	302	304	201	211	130	159	120	130	166	166	
26	97	107	240	280	178	209	108	117	239	249	153	153	302	304	207	211	130	130	111	130	162	168	
26	91	91	231	231	161	176	102	135	243	249	153	157	302	302	203	215	130	131	111	113	168	168	
26	89	99	280	280	186	197	108	110	237	244	153	157	302	306	207	211	130	130	122	124	170	176	
26	89	91	231	240	188	188	119	125	239	245	153	157	302	302	209	211	131	135	111	128	170	172	
26	93	93	225	231	186	195	110	133	239	239	153	153	302	307	199	203	135	161	111	119	166	168	
26	91	95	228	240	166	192	102	108	245	248	153	153	301	301	203	211	130	131	106	111	170	172	
26	95	95	231	280	155	182	108	117	235	245	153	157	302	302	211	215	131	135	111	111	166	170	
26	87	97	280	280	182	192	102	117	243	243	153	153	304	318	209	211	130	150	111	111	170	176	
26	87	97	280	280	182	192	102	117	243	243	153	153	304	318	209	211	130	150	111	111	170	176	
27	95	111	231	231	178	190	110	121	235	251	153	153	302	304	199	209	130	130	111	119	168	170	
27	97	97	231	237	155	182	102	121	239	251	153	153	302	304	199	211	130	130	119	122	168	172	B
27	93	101	237	240	176	182	108	117	239	247	145	153	302	304	199	217	130	154	111	128	166	167	
27	89	91	231	237	176	205	108	135	245	252	153	153	302	304	203	217	133	179	111	119	170	186	B
27	91	97	231	237	155	199	102	110	239	241	153	157	302	302	209	211	130	157	111	122	168	172	
27	93	95	231	280	182	182	113	131	239	240	153	153	302	302	209	215	130	161	111	113	168	172	B
27	97	99	231	240	174	182	117	139	246	253	153	153	302	302	209	209	130	130	117	126	163	168	
27	91	93	228	231	166	184	102	108	239	250	153	153	302	302	209	211	130	161	111	130	168	168	B
27	89	93	240	280	190	190	106	108	239	239	153	157	302	302	209	211	163	167	111	115	168	170	
27	91	95	210	240	174	176	108	133	239	247	153	157	302	319	203	209	130	133	119	124	164	168	
27	99	101	228	231	182	184	106	121	239	249	153	153	302	302	209	211	130	130	111	111	166	168	
27	95	95	237	280	155	178	125	125	239	241	153	153	302	319	209	211	130	154	111	124	164	168	
27	91	107	231	231	163	186	110	133	241	253	153	157	302	302	209	215	130	133	117	119	158	163	
27	91	91	231	231	166	174	108	113	235	237	153	153	302	302	194	215	130	133	111	113	168	170	
27	91	95	231	271	180	195	102	110	247	249	157	157	302	302	203	207	130	157	124	130	167	168	
27	95	95	231	280	161	176	119	121	241	245	153	161	302	302	203	209	130	131	113	130	170	170	
27	95	95	280	280	182	192	106	133	239	252	153	157	302	302	209	216	129	130	111	124	168	168	
27	93	97	231	237	186	190	117	121	243	243	145	153	302	315	194	211	130	130	130	132	168	170	
27	89	89	228	280	166	195	121	129	247	249	153	157	302	306	209	211	130	133	113	126	164	170	
27	95	97	240	280	190	201	110	117	241	243	149	153	302	304	203	223	130	159	111	130	164	168	
27	91	95	231	234	172	176	129	147	239	247	153	153	302	304	209	209	130	157	111	126	167	168	
27	91	95	210	240	184	190	121	149	239	241	153	157	302	319	209	211	130	130	111	119	164	168	
27	93	95	231	234	178	186	108	147	239	245	153	153	302	304	209	209	130	130	111	132	166	168	
27	91	97	231	240	184	213	115	137	245	248	153	157	302	302	203	215	130	133	113	124	170	194	
27	91	91	231	240	155	174	110	139	237	241	153	157	302	302	199	203	133	167	113	117	158	170	
27	89	91	228	231	166	176	113	121	239	239	153	153	302	302	215	216	129	130	124	130	166	167	
27	89	91	231	280	186	195	102	121	245	247	153	157	302	302	209	209	130	159	111	117	164	168	
27	91	91	231	280	176	182	135	145	237	246	153	153	302	311	211	215	130	130	120	126	167	168	
27	91	95	231	274	199	209	110	145	237	239	153	153	311	313	209	211	130	130	113	120	168	180	
27	93	95	228	231	182	211	121	129	239	239	153	153	302	311	199	216	130	161	124	126	167	168	
27	93	97	228	231	186	201	108	110	225	239	153	153	302	302	203	215	130	135	119	124	170	170	
27	91	97	231	231	184	195	102	147	245	256	153	153	302	304	207	209	130	130	111	122	168	170	
27	91	107	231	280	166	209	102	110	239	247	157	161	302	302	211	211	130	163	117	122	167	167	
27	93	97	231	231	174	186	108	117	239	239	153	157	304	308	209	211	130	130	117	122	170	170	
27	93	97	231	234	170	195	121	147	239	249	153	157	302	304	209	211	130	131	119	122	170	170	
27	93	97	231	280	174	186	108	147	239	256	153	157	302	302	209	211	131	133	122	130	170	176	
27	95	97	237	280	184	213	102	117	239	247	153	161	302	304	209	215	130	131	111	113	170	170	
27	93	107	234	280	186	209	102	117	243	247	153	157	302	315	211	211	130	130	119	132	167	170	
27	91	97	231	240	163	199	102	102	239	246	153	157	0	0	0	0	0	0	0	0	0	0	
27	95	107	231	240	176	180	102	117	239	248	157	157	302	302	209	209	130	165	117	124	166	170	
27	91	107	231	231	172	180	102	108	239	243	153	153	302	302	209	211	130	152	111	119	164	170	
27	91	97	231	280	166	184	102	108	237	250	153	157	302	306	211	211	129	131	111	113	170	170	
27	93	95	240	240	176	182	102	117	239	245	153	157	302	306	209	215	130	130	105	122	167	170	
27	91	97	280	280	174	199	102	108	243	246	153	157	302	302	209	211	130	131	105	111	164	170	
27	91	103	240	280	178	184	102	110	237	237	153	153	302	302	203	211	130	131	111	126	168	170	
27	91	93	231	231	176	197	115	139	239	245	153	157	302	304	199	203	130	167	119	122	164	168	
27	97	99	231	231	174	192	108	110	240	245	153	153	304	306	209	211	130	133	117	132	164	164	

27	93	97	228	231	172	213	102	133	240	245	153	153	302	302	203	209	130	130	117	124	168	168	
28	91	97	228	231	170	178	117	119	245	246	153	161	302	302	205	211	130	130	111	128	168	168	B
28	91	91	231	240	166	190	108	119	243	245	153	153	313	313	211	211	130	130	128	134	164	166	
28	91	95	231	280	176	180	117	121	237	239	153	153	302	302	209	211	130	154	119	120	168	170	B
28	89	97	231	280	180	192	121	121	239	243	153	161	302	302	209	213	130	130	120	124	168	170	
28	93	93	225	231	155	176	108	131	241	249	153	153	302	306	203	211	127	169	111	113	166	168	B
28	91	99	231	280	180	192	102	119	239	245	153	153	302	302	203	211	130	154	119	128	170	172	
28	91	93	240	280	184	205	117	139	239	245	153	153	302	302	209	219	131	159	122	124	168	170	B
28	95	97	234	234	192	192	102	135	231	253	153	157	302	302	207	209	130	161	115	119	168	168	
28	91	99	231	231	163	176	108	121	239	241	153	153	288	302	203	213	130	165	120	124	168	170	
28	97	103	231	231	192	199	125	137	239	239	153	153	302	302	209	211	130	146	113	113	170	176	
28	93	95	231	231	192	211	102	102	231	252	153	157	304	304	209	216	130	130	117	130	168	180	
28	95	99	231	231	163	186	102	108	231	243	153	153	302	302	209	217	130	130	111	126	166	172	
29	93	97	231	274	168	184	110	110	247	252	157	161	302	302	205	209	130	175	111	113	170	194	B
29	97	99	231	231	155	184	110	117	245	249	153	157	302	302	209	209	131	175	111	113	166	170	
29	93	95	231	240	180	190	108	110	241	242	153	157	302	302	211	211	129	148	113	128	158	168	B
29	95	103	231	231	166	238	108	110	239	247	157	157	302	302	203	205	127	130	113	124	166	168	
29	87	91	231	240	166	192	108	110	241	249	153	157	302	302	207	215	130	130	113	124	166	194	B
29	95	95	231	234	192	238	110	117	239	249	157	157	302	302	205	213	131	146	111	113	166	168	
29	91	103	231	240	166	199	106	110	246	247	153	157	302	302	207	213	130	146	126	126	166	168	B
29	95	103	231	231	192	238	102	110	239	247	153	153	302	302	205	225	127	133	117	126	168	168	
29	93	97	240	280	180	180	108	119	241	245	157	157	302	302	203	215	130	130	111	113	166	194	
29	95	99	231	231	163	199	110	110	247	249	157	157	302	302	213	213	146	175	111	113	166	168	
29	93	95	231	231	180	199	123	125	241	247	153	153	302	302	205	215	130	146	122	126	166	168	
29	91	95	231	231	192	238	110	113	247	250	153	157	302	302	205	211	130	146	111	113	164	168	
29	87	97	231	234	155	180	108	117	239	247	153	153	302	302	203	211	130	130	111	126	166	168	
29	91	93	240	280	195	211	102	110	247	250	157	157	286	286	199	229	129	130	111	119	166	170	
29	91	93	240	280	211	213	121	133	235	252	153	153	286	302	209	211	127	177	111	115	167	170	
30	91	95	228	231	192	199	110	135	239	239	153	153	302	302	211	211	127	131	111	111	164	168	A
30	87	95	228	234	184	201	110	110	237	253	153	157	302	302	199	209	127	131	111	113	164	172	
30	87	91	228	240	184	205	110	110	247	253	153	153	302	302	209	211	130	146	102	111	164	170	A
30	93	101	231	231	192	192	113	129	239	245	153	153	302	302	203	211	130	131	115	126	164	172	
30	93	97	231	231	155	176	102	131	241	247	149	153	302	304	209	209	130	130	120	128	172	172	A
30	95	97	231	240	155	192	102	135	241	241	153	157	302	302	199	213	130	130	120	128	170	172	
30	91	95	225	280	155	180	127	131	243	244	153	161	302	306	211	227	130	131	120	122	168	168	A
30	91	107	231	280	184	192	102	117	239	242	153	153	302	319	215	215	171	175	113	113	166	168	
30	87	91	231	280	184	184	102	117	252	253	153	153	302	302	203	209	130	157	111	119	170	172	
30	87	87	228	231	161	172	117	117	242	243	157	157	303	303	209	211	130	130	115	130	168	172	
30	97	97	231	280	186	192	102	121	239	265	153	153	303	319	199	211	129	175	111	119	164	170	
30	87	91	222	237	203	205	102	117	239	245	153	153	302	302	199	215	131	146	124	126	170	174	
30	97	107	231	280	184	186	102	145	239	239	153	153	302	319	211	215	130	175	113	119	164	166	
30	93	107	231	231	159	184	102	129	245	247	153	157	302	306	199	203	135	148	111	126	168	170	
30	89	91	231	237	166	238	102	108	239	252	153	157	286	302	199	211	129	130	115	126	170	172	
30	91	99	231	231	168	209	117	117	246	253	153	161	302	302	215	217	130	152	115	119	170	184	
30	87	87	240	240	166	188	117	121	229	245	153	157	303	303	207	229	130	161	115	120	170	172	
30	91	107	234	237	174	213	102	108	239	239	153	157	286	303	199	217	130	130	117	122	168	170	
30	97	107	237	240	166	174	102	121	239	239	153	157	286	303	199	207	129	131	113	117	168	170	
30	93	93	280	280	178	184	108	117	249	249	153	157	303	303	211	217	127	130	113	130	166	170	
30	91	99	231	240	176	182	108	129	235	247	153	161	303	305	203	203	130	135	105	113	166	170	
30	87	89	231	237	184	192	110	135	229	253	157	161	303	303	209	211	127	130	111	128	166	168	
30	99	99	231	240	186	192	102	129	239	247	153	157	303	303	199	203	130	187	105	115	170	172	
30	95	107	228	231	155	174	110	115	245	252	157	157	303	303	215	215	130	148	119	122	170	170	
30	91	97	231	280	182	197	110	117	239	252	153	153	303	304	203	215	127	130	113	126	167	170	
30	93	97	225	231	184	192	102	121	239	243	153	157	303	303	205	209	131	131	111	113	164	170	
30	97	97	228	231	166	205	102	108	239	242	157	161	303	303	209	209	130	130	111	120	170	170	
30	87	97	231	240	166	203	108	121	239	248	153	157	303	303	213	215	130	165	115	122	168	170	
30	91	97	231	280	173	197	117	123	242	249	157	161	303	303	203	216	130	135	117	119	168	170	
30	93	103	231	240	186	195	117	121	252	253	153	153	303	303	199	199	130	148	111	126	168	170	
30	91	93	231	231	180	192	102	121	242	245	153	153	303	303	209	209	148	171	113	124	168	194	
30	91	93	231	231	192	192	110	121	233	245	153	153	306	319	203	209	130	148	111	122	166	194	
30	93	97	231	280	176	213	102	117	243	245	153	153	303	304	203	203	135	177	111	122	166	168	
30	91	99	231	240	178	186	102	121	239	245	153	153	303	303	213	219	130	154	113	120	170	170	
30	93	97	231	280	184	213	102	145	239	248	153	153	302	303	211	213	130	135	111	113	170	172	
30	89	97	231	231	182	184	102	135	239	245	153	157	303	303	199	213	127	130	126	130	168	174	
30	89	93	231	231	190	213	110	127	240	245	153	157	303	303	199	215	130	130	124	130	174	176	
30	91	93	231	234	155	213	117	121	243	245	153	157	303	303	215	215	129	163	117	126	164	166	
30	91	93	228	237	166	192	102	145	239	252	153	153	303	319	207	223	157	167	113	117	170	170	
30	89	103	231	231	192	192	110	121	231	241	153	153	303	303	203	227	130	130	122	124	164	170	
30	91	95	228	231	174	195	102	117	239	244	157	161	286	303	199	209	130	133	117	122	168	172	
30	97	107	231	234	166	192	102	117	247	247	153	157	303	303	209	209	130	154	119	126	170	170	
30	95	103	231	231	166	166	102	135	235	245	153	157	303	303	205	211	127	157	115	124	164	170	
30	87	95	231	237	161	184	121	135	241	253	153	157	303	303	209	211	127	130	115	128	172	172	
30	87	95	231	231	161	184	110	117	239	253	153	157	303	303	209	211	127	130	113	128	172	172	
30	91	93	231	240	172	188	113	129	242	256	157	157	303	303	207	211	130	167	117	126	170	172	
30	87	93	231	280	155	192	106	117	239	239	153	157	303	303	207	215	130	130	117	126	164	168	
30	89	97	237	240	174	188	108	129	235	243	153	153	302	303	209	215	130	130	111	111	168	168	
30	91	93	231	231	182	192	117	121	239	249	157	157	302	304	211	215	130	187	126	126	164	168	
31	93	93	225	231	174	184	117	129	241	241	153	157	302	319	203	209	127	130	119	122	170	170	A
31	93	95	231	231	166	184	108	117	241	244	153	153	302	319	211	221	130	181	115	126	168	172	
31	91	99	231	240	180	182	108	127	245	246	153	153	302	302	211	215	130	173	111	122	166	170	A
31	91	93	231	240	166	184	108	110	241	244	153	157	302	319	211	221	130	130	111	119	168	170	
31	93	95	231	231	184	224	102	129	241	245	153	157	302	319	211	221	130	130	119	120	168	168	A
31	93	95	231	234	182	184	102	108	241	241	153	157	302	319	203	209	130	130	119	126	164	168	
31	93	99	225	280	176	184	108	129	244	246	153	153	302	319	203	211	130	130	126	126	170	170	A
31	95	97	225	231	182	184	117	129	239	241	153	153	302	319	221	223	130	163	119	128	168	168	
31	91	91	231	240	155	174	117	131	246	249	153	153	302	302	213	213	130	130	111	111	170	170	A
31	91	91	231	237	182	195	117	123	239	248	153	161	302	304	203	209	130	130	113	126	168	168	
31	91	93	231	231	174	174	108	119	245	246	153	153	302	337	209	213	130	173	111	113	166	170	A
31	91	95	231	234	168	213	110	117	239	239	157	157	299	302	203	215	130	130	113	119	168	172	
31	95	97	231	234	182	224	102	102	239	246	153	157	302	319	211	211	127	130	113	120	168	168	A
31	91	99	231	231	166	180	110	135	237	241	153	157	302	302	211	213	127	130	111	113	170	170	
31	95	97	231	237	155	182	108	123	241	241	157	157	319	319	209	215	131	135	111	126	164	168	A
31	91	93	225	231	184	192	102	108	241	244	153	157	319	319	209	221	127	131	126	128	166	166	
31	95	129	231	231	186	186	129	135	241	243	153	153	302	319	221	223	127	127	115	119	168	168	
31	91	109	231	240	174	213	102	125	237	249	153	153	300	302	209	213	130	130	119	126	164	170	
31	93	129	228	231	178	227	110	135	243	247	153	153	302	302	209	211	127	130	111	126	167	168	B
31	91	93	231	231	184	184	129	133	241	244	153	153	319	319	199	221	127	130	113	119	168	168	
31	95	97	225	280	184	199	110	129	243	244	153	153	304	319	203	211	127	130	111	119	167	170	A
31	91	99	234	240	182	201	110	133	245	246	153	153	302	319	199	215	127	130	126	128	166	167	
31	95	99	231	231	166	186	108	135	241	245	153	157	300	319	205	215	131	131	115	128	170	170	B
31	91	93	231	231	155	184	129	131	241	241	153	153	319	319	213	221	127	127	119	119	168	176	
31	91	99	231	240	195	201	110	121	241	249	153	157	302	302	197	215	130	130	111	126	164	167	A
31	89	91	240	280	180	188	110	121	248	249	153	157	302	302	209	211	130	130	111	115	166	166	
31	91	95	231	231	176	192	110	110	243	243	153	153	319	319	199	215	130	135	113	120	168	170	A
31	95	107	231	240	174	184	125	129	239	245	153	153	302	319	199	213	130	130	119	120	166	167	
31	91	95	231	231	176	192	110	110	243	243	153	153	319	319	199	215	130	135	113	120	168	170	A

31	91	91	231	240	180	190	108	110	243	246	157	157	302	302	199	211	130	130	111	128	167	170	
31	91	95	231	231	180	184	108	129	243	248	153	157	302	302	199	215	135	173	120	122	166	167	A
31	89	97	231	231	192	195	110	121	239	239	153	153	300	302	199	221	127	135	111	132	168	170	
31	95	107	240	240	182	207	110	123	237	241	153	157	302	302	203	215	130	131	113	126	164	166	A
31	89	107	231	240	155	184	108	110	239	253	153	157	302	302	199	213	130	130	111	111	168	170	
31	89	91	231	231	186	192	102	110	239	249	153	153	286	319	199	223	130	130	111	113	166	170	A
31	95	95	231	231	184	213	117	129	243	245	153	153	302	302	199	209	130	135	111	120	166	167	
31	95	107	240	240	182	205	110	123	237	241	153	157	302	302	203	215	130	131	113	126	164	166	A
31	91	97	228	237	186	192	121	129	239	243	153	153	302	308	209	211	127	167	119	124	170	172	A
31	95	95	231	280	172	186	117	117	243	249	153	157	302	302	203	211	130	163	113	122	167	174	B
32	95	95	231	231	188	203	108	108	247	252	153	157	302	302	199	211	130	131	120	126	164	166	A
32	91	97	231	240	184	188	119	121	245	253	153	153	302	302	205	211	127	133	124	132	162	170	
32	95	99	231	231	186	199	102	108	239	247	153	153	302	302	209	209	130	130	120	126	164	170	A
32	93	95	280	280	161	199	100	108	247	247	153	157	302	302	199	215	130	130	120	124	170	170	
32	95	97	231	280	180	199	102	121	247	247	157	157	302	302	199	209	130	130	111	111	164	168	A
32	89	91	231	280	172	182	121	121	239	249	153	153	302	302	207	215	154	161	115	132	168	170	
32	93	95	280	280	161	199	100	108	247	247	153	157	302	302	199	215	130	130	120	124	170	170	A
32	93	97	231	280	168	192	108	110	243	243	153	153	302	302	203	213	130	161	120	126	164	170	
32	87	91	231	280	188	266	102	102	239	245	153	157	302	302	205	209	127	130	120	130	168	194	
32	93	95	231	240	186	213	102	102	241	241	153	153	302	306	209	211	127	161	111	124	167	170	
32	91	91	231	240	166	182	108	131	241	252	153	157	302	304	203	209	127	130	119	126	168	172	
32	95	95	231	280	186	188	108	123	239	252	153	153	302	304	199	211	127	130	124	126	194	194	
32	91	93	231	280	184	199	102	123	247	247	157	157	300	304	203	219	130	135	111	128	170	172	
32	91	97	280	280	166	184	102	110	243	253	153	157	302	304	209	221	135	150	126	128	170	172	
32	91	103	231	237	190	213	102	121	247	252	153	153	302	302	203	203	127	167	113	115	168	170	
32	91	95	231	240	188	195	102	117	245	253	157	157	302	304	203	219	130	131	122	128	168	170	
32	87	97	234	280	182	184	102	119	248	253	153	157	304	304	213	219	131	161	122	124	170	172	
32	89	93	231	280	192	205	108	110	244	249	153	153	302	304	211	213	130	133	124	126	167	168	
32	87	97	231	240	188	242	108	117	239	265	153	157	304	319	209	215	130	130	113	126	166	167	
32	87	93	231	240	155	168	108	110	247	249	153	153	302	319	205	215	127	131	113	122	166	167	
32	93	93	231	231	195	205	108	127	249	249	153	153	302	304	211	213	130	165	115	130	168	172	
32	89	91	234	280	195	195	102	117	239	245	157	157	302	304	203	211	130	131	111	124	170	170	
32	95	107	231	240	178	192	108	121	239	249	153	153	300	302	207	211	127	173	113	132	166	168	
32	89	89	231	234	184	205	102	102	243	243	153	153	302	302	211	211	130	167	113	119	168	170	
33	91	93	231	231	195	195	102	108	248	252	157	161	302	302	203	209	130	130	120	128	172	172	A
33	91	99	228	231	182	184	102	127	243	249	153	157	302	302	211	213	130	165	120	130	170	170	
33	91	93	228	231	166	207	110	117	239	245	153	157	302	302	203	211	130	131	111	119	168	168	A
33	93	93	231	240	163	205	102	137	240	243	153	161	300	302	211	213	127	163	128	144	168	168	
33	91	107	231	231	192	195	123	127	241	245	157	157	302	302	201	209	138	157	105	111	167	168	A
33	89	95	231	231	163	205	117	127	237	249	153	161	302	306	211	221	130	131	113	122	166	170	
33	89	91	231	231	155	195	127	131	241	248	153	161	301	306	209	221	130	130	111	111	166	176	A
33	91	93	231	234	192	192	102	117	237	240	153	153	302	306	199	229	130	138	113	128	168	170	
33	91	93	231	234	192	192	102	117	237	240	153	153	302	306	199	229	130	138	113	128	168	170	
33	91	95	231	234	155	166	119	127	241	251	157	161	302	302	201	209	127	138	119	124	168	168	
33	93	95	237	280	182	190	102	121	239	245	153	153	302	302	199	211	131	181	111	113	167	170	
33	91	97	234	234	176	178	102	131	243	243	153	153	302	304	219	223	130	135	111	111	164	170	
33	95	101	231	280	161	190	102	127	245	245	145	157	302	304	199	229	131	148	111	113	168	172	
33	93	93	228	240	166	190	127	139	239	241	153	153	302	302	199	211	127	131	111	113	168	170	
33	95	95	231	280	161	190	117	127	239	249	145	153	302	302	213	215	130	131	111	113	167	170	
33	91	91	231	280	174	192	110	131	239	247	153	161	302	304	209	209	130	148	111	111	166	168	
33	93	93	237	280	182	182	119	121	241	249	153	153	302	302	211	229	135	181	111	113	166	167	
33	91	93	231	240	182	192	108	110	241	249	153	157	302	302	209	211	131	135	111	113	168	170	
33	93	97	231	237	166	174	106	129	243	245	153	153	302	302	209	211	131	131	111	124	158	168	
33	91	93	231	231	176	182	113	125	239	245	153	153	302	304	207	229	131	131	113	130	164	168	
33	91	97	231	240	192	192	108	129	235	235	153	153	302	302	215	229	130	152	105	113	164	168	
33	95	95	231	240	161	178	117	117	239	241	153	153	302	304	205	207	130	130	126	128	166	167	
33	93	97	231	231	182	186	102	113	245	249	153	153	302	304	215	223	130	130	124	126	164	170	
33	93	97	231	231	163	192	108	137	229	235	153	157	302	302	211	229	130	130	117	119	164	168	
34	91	95	231	231	192	205	131	133	235	239	153	153	302	306	209	209	130	154	119	124	168	170	
34	91	99	228	280	209	219	110	151	239	253	153	153	302	302	211	213	131	131	119	145	168	194	
34	93	97	231	231	195	197	102	113	241	247	153	161	302	302	203	215	130	165	113	122	168	170	
34	87	93	231	280	170	178	110	137	239	242	153	153	302	302	209	215	130	161	111	122	168	172	B
34	91	93	231	231	155	180	102	110	241	245	153	161	302	302	215	215	130	131	113	126	168	174	
34	91	97	231	280	155	166	102	108	235	249	153	153	301	327	209	209	130	130	111	113	168	168	
34	91	107	231	231	188	195	110	127	241	245	153	153	302	302	215	215	127	146	111	117	168	170	
34	0	0	0	0	0	0	0	0	0	0	0	0	302	308	203	229	130	165	113	126	168	170	
34	91	103	240	280	155	190	110	121	239	245	153	157	301	304	211	215	130	130	111	122	164	168	
34	89	97	231	231	155	188	102	121	241	249	153	153	302	327	209	215	130	167	105	113	168	172	
34	89	91	234	234	155	184	102	121	241	249	153	157	302	304	209	219	130	169	113	126	168	170	
34	95	97	216	219	182	197	110	119	239	247	157	161	302	304	203	207	131	131	111	111	164	168	
34	91	139	231	280	155	195	102	110	239	247	153	153	301	302	203	209	130	130	113	122	168	170	
34	91	91	231	280	155	186	102	110	241	249	153	153	301	302	199	205	130	130	115	119	168	168	B
34	91	91	231	231	155	155	110	129	249	250	153	153	302	302	199	213	130	130	119	126	164	168	
34	91	95	237	280	166	192	121	129	235	245	153	157	302	327	215	215	130	130	111	128	168	168	
34	91	95	231	280	166	166	102	121	239	243	153	161	302	302	199	209	130	130	111	120	168	170	
34	91	95	231	280	166	166	106	121	235	239	153	153	302	327	209	215	130	130	111	113	168	170	
34	91	91	231	280	166	166	102	121	235	250	153	153	302	327	199	215	130	130	113	120	168	168	
34	91	93	231	231	182	182	110	121	239	245	153	153	302	327	203	215	130	130	113	124	168	168	
34	91	95	225	280	205	205	108	121	239	249	153	157	302	327	211	215	130	130	111	119	167	170	
34	91	99	231	280	197	197	108	121	235	252	153	153	308	327	215	215	129	130	113	113	170	170	
34	91	97	231	231	166	203	110	121	235	249	153	157	302	302	209	209	130	130	111	113	164	170	
34	87	95	231	231	166	166	102	121	229	235	153	153	302	327	205	215	130	131	113	130	168	170	B
34	91	95	231	231	190	190	108	110	243	253	153	153	299	302	197	209	130	175	119	119	168	194	
34	91	95	231	231	190	203	102	108	237	245	153	153	302	302	199	211	130	161	115	122	168	170	
34	87	95	231	231	178	190	108	110	249	253	153	157	315	315	209	209	130	131	124	126	166	168	
34	87	91	225	231	186	188	113	121	237	241	153	157	302	302	199	211	130	177	105	111	170	194	
34	95	97	231	237	173	197	102	110	235	241	157	161	302	304	211	215	130	165	113	117	168	168	
34	93	93	231	231	178	195	102	113	245	247	153	157	301	301	203	203	129	130	122	124	170	170	
34	91	99	225	231	159	166	102	113	241	249	153	157	302	315	199	229	130	175	111	113	168	176	
34	0	0	0	0	0	0	0	0	0	0	0	0	301	303	213	215	130	152	105	119	168	172	B
35	95	95	228	231	176	213	121	135	239	241	153	157	304	311	211	215	129	130	111	113	166	168	
35	95	95	228	231	176	213	121	135	239	241	153	157	304	311	211	215	129	130	111	113	166	168	
35	95	97	231	231	174	213	102	102	240	245	153	153	304	304	199	203	130	130	111	111	164	170	
35	99	99	231	237	155	222	102	110	245	249	153	153	302	306	211	229	130	135	128	128	166	180	B
35	93	95	228	231	176	195	108	110	239	241	153	153	302	302	211	211	129	138	105	111	168	172	
35	91	97	231	240	172	199	102	121	239	252	153	153	302	304	209	213	131	135	111	126	170	170	
35	89	103	231	231	172	172	121	143	242	245	153	153	302	302	209	213	130	159	111	132	170	170	
35	87	95	231	280	188	192	102	117	229	245	153	157	302	306	203	209	130	130	113	115	168	170	
35	95	97	225	231	155	219	110	131	240	247	153	161	290	302	203	203	130	130	120	120	168	170	
35	91	93	280	280	176	195	121	129	239	245	153	161	302	302	203	203	154	175	113	124	168	168	
35	95	103	228	237	176	213	102	102	239	245	153	161	302	302	209	217	129	131	111	126	166	168	
35	93	95	234	280	172	176	108	117	239	239	157	157	302	302	199	211	130	130	105	115	158	170	
35	95	97	237	280	155	182	102	113	245	246	153	153	302	302	215	229	130	175	113	120	168	168	
35	91	95	237	280	182	199	102	106	239	239	153	161	302	302	209	211	154	154	115	120	180	194	B
35	95	95	234	237	155	195	108	121	235	239	153	153	301	302	209	209	130	154	122	128	170	174	
35	87	97	231	280	176	192	110	125	237	239	153	161	302	314	215	215	129	152	115	128	168	172	

35	87	97	231	240	176	201	110	129	235	241	153	161	302	302	211	213	129	130	105	111	166	168	
35	91	91	225	280	197	219	108	131	247	249	161	161	302	302	211	213	130	130	120	120	168	170	
35	95	95	231	231	176	180	113	123	239	239	157	161	302	302	209	225	131	159	117	120	170	174	
35	87	95	228	231	176	176	102	110	241	245	153	153	302	302	209	211	129	181	111	122	166	168	
35	87	99	228	231	176	178	102	110	243	245	153	153	302	302	209	209	129	154	111	120	166	170	
35	93	95	228	231	190	195	108	108	249	255	153	157	302	302	203	209	130	131	115	134	168	180	
35	93	101	237	237	166	184	115	131	243	249	153	161	302	302	199	211	131	131	111	115	166	168	
35	91	91	231	231	180	180	106	108	241	250	153	153	302	303	209	209	130	167	113	120	194	194	B
35	91	95	228	244	173	190	108	110	243	255	157	157	302	302	203	215	130	130	113	134	168	174	
35	91	95	228	244	173	190	108	110	243	255	157	157	302	302	203	215	130	130	113	134	168	174	
35	91	97	228	231	170	186	102	108	245	249	153	153	302	302	199	211	131	169	117	120	168	174	
35	91	97	231	237	176	188	102	102	239	255	157	157	302	302	203	213	130	130	111	113	164	168	
35	93	97	237	240	184	190	115	129	243	250	149	161	302	308	199	211	129	131	111	115	166	170	
35	95	97	231	280	213	219	106	108	245	245	153	153	302	306	209	211	130	131	111	115	170	174	
35	87	97	237	280	184	192	102	108	239	255	153	161	302	306	205	223	127	133	120	122	168	170	
35	97	97	240	280	184	192	119	121	243	249	153	153	302	302	209	223	130	131	113	126	170	170	
35	91	95	231	240	186	186	102	108	241	245	153	153	302	302	209	209	131	131	119	128	164	180	
35	95	97	280	280	155	186	102	110	239	245	153	161	301	302	205	209	135	169	115	128	168	174	B
35	91	95	240	280	155	186	102	110	239	245	153	161	301	302	205	211	165	169	115	124	168	168	
35	91	103	231	280	176	195	110	129	239	249	153	153	302	302	203	203	129	175	113	119	168	170	
35	99	101	228	280	155	166	102	121	239	243	153	161	302	304	199	203	130	131	115	124	164	168	
35	87	91	240	280	155	180	102	110	245	245	153	161	301	302	203	205	129	165	124	144	168	170	
35	97	103	280	280	176	176	110	121	239	249	153	153	302	302	201	203	130	150	120	120	168	168	
35	97	103	228	231	172	180	121	143	239	245	153	153	302	302	209	211	130	135	111	115	168	170	
35	103	103	280	280	176	195	110	110	247	249	153	153	302	302	203	203	150	175	113	120	168	168	
35	103	103	280	280	176	195	110	110	247	249	153	153	302	302	203	203	150	175	113	120	168	168	
35	89	99	231	231	155	199	102	129	245	247	153	153	306	306	209	215	130	150	113	113	166	168	
35	95	97	237	280	178	197	102	110	235	239	157	157	302	302	209	215	131	135	115	126	170	194	
35	99	103	231	234	190	195	108	108	245	245	153	157	302	302	203	211	134	135	115	130	164	170	
35	95	99	231	231	219	222	102	125	239	249	157	157	301	301	209	217	131	165	111	128	166	170	
35	95	109	231	280	155	203	102	131	239	241	153	157	302	302	199	217	130	131	113	115	168	170	
35	89	93	240	280	195	203	102	102	235	239	153	157	302	306	209	211	130	167	115	130	164	170	
36	91	97	228	228	176	178	137	137	235	243	153	153	302	304	203	203	129	131	119	120	168	170	
36	91	93	231	237	172	180	108	119	239	252	153	157	302	302	211	213	130	131	113	117	162	186	
36	89	91	231	237	199	203	106	121	241	249	153	153	302	302	203	203	131	175	115	119	168	186	
36	91	93	231	231	180	219	106	125	233	239	153	157	302	304	203	213	130	131	113	140	168	186	B
36	83	95	225	231	159	188	102	121	239	243	153	161	302	304	203	209	130	131	115	130	170	170	
36	91	95	231	240	155	163	110	117	248	255	153	161	302	302	209	215	131	131	111	120	168	170	
36	93	95	231	240	188	188	108	117	245	245	153	153	301	306	219	229	130	167	126	134	164	170	
36	93	95	231	280	190	201	113	125	241	247	153	153	290	302	199	211	130	131	111	119	168	170	
36	97	109	228	231	188	195	102	113	247	251	153	157	302	302	203	203	129	130	120	128	168	170	
36	99	107	228	231	176	213	106	121	237	243	153	153	302	302	205	211	130	131	111	113	164	168	
36	91	97	237	280	182	205	119	119	239	247	153	153	288	306	213	215	127	130	105	120	164	168	
36	91	95	240	280	172	188	108	117	245	251	153	153	302	306	209	215	130	130	111	120	168	170	
36	103	107	231	231	195	203	100	113	237	241	153	157	302	302	203	203	129	130	115	122	168	174	
36	91	95	231	231	155	180	108	125	239	239	153	161	302	302	197	217	130	130	115	119	164	168	B
36	91	93	228	231	182	190	106	121	247	249	153	153	302	304	199	207	131	167	111	134	166	170	
36	91	101	237	280	190	207	102	119	241	245	153	157	302	302	209	209	130	130	111	128	164	168	
36	0	0	0	0	0	0	0	0	0	0	0	0	304	306	203	211	130	130	113	119	164	164	
36	95	97	231	237	184	188	102	106	245	251	153	153	304	306	209	209	130	130	111	115	168	174	
36	87	95	231	231	155	190	121	121	239	249	153	157	290	302	203	203	130	175	115	119	166	168	
36	95	95	231	231	184	190	110	131	239	251	153	157	304	304	209	209	130	130	111	115	168	194	
36	91	95	231	231	172	182	110	139	239	245	153	153	302	302	203	209	127	133	111	132	164	166	
36	91	95	231	280	180	188	102	106	241	243	153	153	302	306	211	211	130	131	105	113	168	170	
36	91	95	231	280	184	213	102	119	241	241	153	153	302	302	199	211	154	179	113	115	168	168	
36	93	119	234	237	197	201	110	125	235	251	153	153	0	0	0	0	0	0	0	0	0	0	B
36	93	97	231	231	199	205	102	113	239	241	153	153	302	302	203	207	130	167	111	128	166	168	
36	95	97	231	237	188	205	119	125	239	241	153	153	302	302	203	203	167	185	111	111	166	168	
36	91	95	231	240	155	166	137	145	235	235	153	157	302	302	205	209	130	130	115	117	168	170	
36	93	95	225	234	199	201	102	125	235	239	153	157	302	302	207	217	130	131	111	111	166	168	
36	99	109	228	231	197	213	110	117	245	247	153	161	306	306	209	209	130	130	113	126	168	170	
36	97	109	231	237	176	199	108	125	239	245	149	153	302	302	203	211	129	130	113	126	170	172	
36	93	103	228	237	188	190	117	123	237	251	153	157	302	302	203	203	130	131	111	111	168	170	
36	91	95	231	237	155	213	102	108	241	247	157	157	302	302	209	217	127	131	115	117	168	170	
36	95	97	280	280	186	202	106	108	239	239	153	157	302	302	209	211	131	131	117	128	162	168	
36	89	91	228	280	155	199	106	123	239	245	153	161	302	302	217	219	127	130	113	113	168	170	B
36	91	93	280	280	190	213	100	131	241	249	153	153	302	315	205	209	127	135	120	128	168	170	
36	93	95	237	237	188	195	100	121	241	249	153	157	301	303	203	213	130	165	111	120	168	168	
36	91	93	231	231	188	197	102	110	239	251	153	157	299	302	205	215	165	165	115	119	164	166	
36	89	95	237	237	174	190	121	121	239	241	153	153	302	303	203	203	130	131	111	126	170	170	
36	97	97	228	237	180	213	106	106	239	245	153	157	301	301	209	209	127	127	113	128	168	170	
36	93	109	231	280	184	192	106	117	239	243	153	157	302	302	203	211	131	131	115	120	168	170	
36	0	0	0	0	0	0	0	0	0	0	0	0	302	302	203	211	131	131	115	120	168	170	
36	93	93	231	237	180	219	106	117	241	245	153	157	302	302	209	209	130	163	113	126	164	166	
36	95	95	237	280	180	184	106	113	237	255	153	157	301	341	203	213	130	131	111	113	164	168	
36	93	97	225	231	163	172	123	129	249	251	153	153	291	311	209	217	131	138	111	111	164	168	
36	91	97	231	231	186	219	102	110	243	250	153	161	302	302	203	213	130	161	119	122	168	172	
36	95	97	231	280	182	182	108	119	247	248	153	153	302	306	199	209	161	165	111	120	168	168	
36	91	93	231	237	182	188	102	110	237	243	153	157	302	302	203	213	131	161	113	120	168	172	
36	91	97	231	240	182	192	113	137	245	249	153	153	302	302	199	203	130	148	105	111	168	170	
37	89	91	231	280	174	213	102	108	237	247	153	157	301	303	199	213	130	130	111	115	166	170	
37	89	91	231	237	174	180	102	106	239	251	153	157	301	301	199	209	130	144	111	119	168	170	
37	95	97	225	231	180	186	102	121	239	241	145	153	301	301	211	215	130	138	102	119	168	170	
37	91	93	231	231	161	180	108	137	247	247	153	153	301	301	213	219	127	130	113	130	164	168	B
37	87	97	234	280	155	182	106	106	245	254	153	153	301	301	209	213	130	165	111	115	168	170	
37	91	93	280	280	180	192	110	135	231	243	153	157	301	301	203	213	130	130	115	119	166	170	
37	91	103	219	231	166	184	102	108	241	243	153	153	301	305	203	209	130	157	113	119	170	170	
37	91	91	231	231	182	195	102	108	243	249	153	157	301	301	211	229	130	130	113	113	166	168	
37	91	91	231	231	182	182	108	121	239	243	157	161	301	301	209	213	135	140	113	126	166	168	
37	87	91	225	237	195	211	102	110	231	241	157	161	301	303	199	215	130	179	111	113	168	168	
37	87	91	231	231	188	195	102	117	241	254	153	153	301	301	197	229	130	130	113	126	168	168	
37	93	109	231	240	180	190	127	137	241	255	153	153	301	303	203	209	130	131	111	124	158	164	
37	91	91	228	280	184	192	102	102	241	249	153	153	301	303	203	229	130	130	120	126	164	176	
37	95	101	231	244	157	205	106	123	235	243	153	153	301	301	209	213	130	131	111	122	168	170	B
37	97	101	228	231	184	211	119	129	235	249	157	157	300	301	199	199	130	130	111	115	172	186	
37	89	91	231	280	166	192	102	137	239	249	153	153	303	310	199	211	130	130	113	126	168	178	
37	89	91	231	280	166	192	102	137	239	249	153	153	303	310	199	211	130	130	113	126	168	178	
37	91	93	231	280	168	203	108	121	239	241	157	161	301	301	203	209	130	131	117	122	162	164	
37	89	91	231	237	166	176	110	121	239	254	157	157	301	301	197	229	130	135	113	128	164	170	
37	91	91	231	240	163	188	102	129	239	245	153	153	301	301	203	221	129	131	109	111	172	190	
37	95	99	231	240	172	213	108	119	245	250	153	161	301	301	203	229	127	130	115	124	162	190	
37	91	97	231	280	155	213	119	127	237	261	153	153	298	301	203	209	131	131	111	124	166	168	
37	91	101	280	280	172	192	102	108	239	243	153	161	301	310	203	211	130	130	111	119	164	164	
37	91	95	231	240	182	190	117	147	243	249	153	161	301	301	199	209	130	131	111	119	162	168	B
37	91	91	231	280	176	188	102	108	245	248	153	157	301	301	203	203	131	131	115	120	166	170	
37	95	101	231	280	195	213	123	123	243	244	153	153	301	301	203	213	130	144	115	126	168	168	

37	93	101	231	244	176	195	123	123	235	245	153	153	301	301	209	209	131	179	111	122	168	168	
37	87	93	237	240	197	213	131	139	241	243	153	161	301	301	203	211	131	169	111	122	170	170	
37	91	93	231	237	172	203	108	141	247	250	153	157	288	288	211	215	130	130	111	113	168	180	
37	91	97	231	231	176	180	108	121	237	241	153	161	301	301	209	211	130	130	111	126	164	168	
37	93	93	234	237	186	215	115	133	229	243	149	161	301	301	203	209	131	159	111	111	168	170	
37	91	93	234	280	180	213	102	133	235	247	153	153	301	303	209	213	130	130	111	113	164	166	
37	91	95	231	280	155	176	117	139	235	241	157	161	301	303	203	209	130	131	115	122	170	178	
37	95	99	231	231	184	186	108	129	243	243	153	153	301	301	203	215	130	130	113	120	166	168	B
37	91	91	231	231	176	186	102	129	241	243	153	161	301	301	203	203	130	130	111	120	168	170	
37	91	99	207	231	176	203	102	113	247	248	153	161	301	301	203	209	130	130	113	115	170	170	
37	91	93	231	231	182	205	113	139	235	239	153	157	301	301	203	209	130	130	111	119	170	174	
37	93	99	231	237	176	180	102	102	248	249	153	161	301	301	201	209	130	138	111	122	168	170	
37	93	95	228	280	192	201	113	113	245	251	153	153	301	301	203	216	130	130	113	120	168	172	
37	91	95	231	280	155	190	108	119	237	248	153	161	301	301	203	203	131	144	113	126	164	164	
37	95	99	231	280	172	191	108	108	239	248	153	153	301	301	203	223	129	154	105	113	168	168	
37	91	95	231	280	180	186	129	137	241	249	153	153	301	301	209	211	130	157	113	124	168	176	
37	93	93	231	237	178	182	102	139	231	239	153	153	301	301	209	211	130	130	111	130	164	170	
37	93	103	237	280	155	180	108	123	244	249	153	157	301	314	201	209	130	131	115	122	164	164	
37	91	101	240	280	172	213	108	133	239	247	153	153	301	328	209	213	127	130	111	115	170	178	
37	93	93	231	280	155	195	102	108	241	247	153	161	301	301	199	217	131	133	113	126	164	170	
37	91	93	237	237	180	205	106	108	241	241	157	157	301	301	203	211	130	135	126	132	168	170	
37	91	97	280	280	182	182	106	108	239	251	153	153	301	301	209	209	130	181	115	117	166	168	
38	95	97	231	240	166	180	108	110	237	254	153	153	302	306	213	229	129	130	111	119	167	168	
38	91	97	240	280	166	211	106	110	239	245	153	157	302	302	209	213	130	130	111	138	168	168	
38	91	99	240	280	176	188	102	121	247	251	153	157	302	302	205	211	127	130	111	120	166	168	
38	95	101	231	231	178	188	108	145	237	245	153	153	301	302	207	219	130	175	111	132	166	170	B
38	91	97	231	250	182	192	102	121	249	254	153	153	302	302	217	219	130	130	111	122	168	168	
38	91	95	231	244	166	176	102	121	247	254	153	157	302	304	219	219	130	148	115	122	168	168	
38	93	97	231	231	166	180	102	121	245	247	153	153	302	304	209	219	127	130	111	111	167	168	
38	91	91	231	280	178	188	110	119	245	257	153	157	301	301	207	219	161	175	126	126	164	170	
38	95	101	231	231	178	188	108	145	237	245	153	153	301	302	207	219	130	175	111	132	166	170	
38	91	95	231	231	180	182	102	108	237	247	153	153	302	306	211	229	129	130	111	119	167	168	
38	91	95	231	240	166	182	102	137	243	247	153	153	302	302	199	213	127	131	115	120	168	170	
38	95	95	231	231	180	186	102	108	237	245	157	157	302	302	229	229	127	129	119	119	167	168	
38	93	101	231	231	180	190	106	110	247	249	153	157	302	302	219	219	130	131	115	117	168	170	
38	91	93	231	231	188	188	108	110	237	254	153	153	302	306	219	229	127	130	111	119	167	168	B
38	95	97	244	280	172	186	102	106	241	247	153	157	302	302	213	213	127	163	119	119	168	180	
38	97	103	231	240	176	182	110	110	245	246	153	157	304	304	219	219	130	130	111	122	168	172	
38	99	117	231	231	176	190	106	113	241	247	153	157	302	302	203	219	130	169	111	115	170	170	
38	87	95	237	237	188	199	108	121	237	247	157	157	303	303	209	219	127	130	115	144	170	194	
38	93	95	231	237	176	211	108	119	239	241	153	157	302	302	203	219	127	130	119	126	168	170	
38	93	97	231	231	186	215	102	113	247	252	153	157	302	304	215	219	130	175	119	126	170	170	
38	99	99	237	240	188	188	102	108	241	247	153	157	302	302	213	219	127	169	111	115	168	170	
38	91	91	237	240	176	176	102	121	247	249	153	157	302	302	205	211	130	152	111	111	166	168	
38	91	109	240	280	184	184	102	129	243	254	153	153	302	302	199	205	127	131	111	111	168	168	
38	99	99	240	280	188	195	102	102	243	254	153	161	302	302	205	221	130	152	111	119	168	174	B
38	91	109	280	280	176	195	102	102	243	251	157	161	304	304	199	219	130	131	111	117	166	168	
38	91	99	225	231	184	186	125	129	241	243	153	157	305	305	203	221	130	135	119	126	168	172	
38	91	109	225	240	184	188	102	102	243	254	153	161	302	305	205	221	130	152	111	111	168	174	
38	99	99	280	280	176	195	102	121	243	249	153	157	302	304	199	219	130	130	117	119	168	174	
38	91	93	234	237	176	184	102	121	239	251	157	157	302	302	211	211	130	130	111	126	166	168	
38	99	99	237	280	176	184	102	129	243	251	157	161	302	302	219	221	130	130	111	119	168	174	
38	91	99	225	280	176	195	102	121	243	251	157	161	302	304	199	219	130	131	111	117	166	168	
38	95	99	231	280	176	199	108	117	245	249	157	157	302	302	215	217	131	131	113	122	172	174	
38	95	99	231	280	172	176	115	121	245	247	153	153	303	303	215	219	130	130	113	117	164	174	
38	93	109	231	280	186	195	123	123	241	247	153	157	303	303	203	211	127	159	111	111	170	170	B
38	91	95	231	250	184	192	102	123	243	249	153	153	303	303	211	213	130	131	111	115	170	172	
38	99	109	225	237	176	195	121	129	247	249	157	161	303	303	199	219	130	130	111	115	168	168	
38	91	95	225	250	184	192	102	123	243	249	153	153	303	303	203	217	130	131	111	115	168	172	
38	93	97	237	280	178	186	121	125	249	250	153	157	303	305	203	203	130	130	119	124	168	172	
38	91	95	231	234	176	192	102	119	241	249	153	153	303	303	203	213	130	152	115	134	168	180	
38	91	95	231	231	166	176	119	121	239	241	153	161	303	303	209	213	127	130	113	134	164	180	
38	95	99	231	240	172	186	102	129	239	245	153	153	0	0	0	0	0	0	0	0	0	0	
38	91	97	231	280	184	191	102	106	241	243	153	153	0	0	0	0	0	0	0	0	0	0	
38	95	99	231	231	155	182	102	137	249	249	153	153	301	301	211	213	130	131	122	126	168	168	
38	93	97	231	231	155	180	110	110	243	249	153	161	296	303	203	211	130	133	113	113	170	176	
38	87	97	280	280	157	199	108	110	239	245	153	157	301	301	211	217	130	130	113	117	166	166	
38	87	95	280	280	195	199	108	108	239	251	153	157	301	301	211	211	130	130	111	117	166	170	
38	95	95	280	280	199	248	108	110	243	251	157	161	301	301	211	217	130	130	117	117	167	168	
38	93	95	231	280	155	195	102	137	243	243	153	153	301	301	203	211	127	131	117	126	166	172	
39	0	0	0	0	0	0	0	0	0	0	0	0	302	302	209	209	129	131	122	126	168	170	
39	89	91	228	240	182	192	113	125	248	249	153	153	301	306	203	209	130	133	117	122	170	172	
39	91	93	231	231	168	209	125	151	229	249	153	157	301	302	203	213	130	131	124	126	170	170	
39	87	87	231	240	166	176	110	119	237	237	145	153	302	302	203	209	133	133	117	119	164	170	B
39	89	91	231	231	192	215	119	131	241	249	153	157	301	301	205	215	130	133	111	122	168	168	
39	97	99	225	280	186	192	102	110	241	252	153	157	302	311	203	209	130	140	111	115	168	170	
39	89	91	231	237	215	224	102	121	241	248	153	157	302	302	194	209	130	175	113	119	166	168	
39	93	99	231	231	155	182	102	131	239	246	153	157	302	302	209	213	130	138	126	145	168	168	
39	95	97	225	237	186	209	108	121	237	241	153	153	302	302	209	215	130	157	105	130	168	168	
39	91	95	231	240	184	215	102	131	247	252	153	153	302	302	205	211	130	183	111	130	164	166	
39	99	107	228	231	190	250	102	110	245	252	157	157	302	302	209	213	131	138	115	117	164	170	
39	93	93	231	240	166	197	113	117	237	245	153	157	302	302	203	211	131	167	113	124	168	172	
39	93	93	231	280	186	197	110	117	243	245	153	161	302	302	203	213	146	165	111	136	168	172	
39	89	95	231	231	155	205	110	131	241	249	153	153	302	302	203	213	131	165	124	126	168	170	B
39	91	93	231	231	157	168	119	119	245	247	153	157	302	302	199	199	130	130	111	128	168	168	
39	93	99	231	240	182	192	102	106	241	246	153	153	302	302	209	211	130	130	111	126	170	170	
39	95	111	237	240	155	190	102	121	237	242	153	157	289	289	201	229	129	130	115	119	166	168	
39	91	99	231	240	155	192	102	110	237	246	153	157	302	302	209	211	130	130	113	126	168	170	
39	93	95	234	240	184	192	110	113	245	252	153	157	302	302	211	211	130	131	115	124	168	170	
39	89	97	222	231	186	190	108	127	239	248	153	153	302	302	203	209	131	157	113	124	170	176	
39	91	97	231	280	184	213	102	110	229	237	153	153	302	302	211	215	130	130	124	124	168	178	
39	91	97	234	240	180	184	121	125	235	241	157	161	301	302	209	217	130	131	126	126	168	170	
39	89	93	231	240	161	190	102	121	241	245	157	157	297	302	209	209	130	133	111	120	164	170	
39	93	93	240	280	168	195	125	139	248	249	153	157	301	302	201	205	131	152	122	122	170	182	B
39	95	101	228	231	185	199	119	121	239	244	153	157	302	302	209	215	130	133	124	126	172	186	
39	91	93	225	280	174	188	102	108	237	243	153	161	302	302	199	203	129	131	115	126	164	166	
39	91	97	231	231	203	224	102	121	241	244	153	153	301	302	209	215	130	131	111	124	166	170	
39	93	95	231	280	168	182	110	119	239	243	153	157	301	306	199	211	131	177	111	113	168	170	
39	93	95	231	231	172	203	102	110	241	242	153	153	302	304	219	219	129	130	111	117	166	168	
39	91	93	231	231	168	203	110	119	239	247	153	157	302	302	209	219	130	152	111	126	166	168	
39	93	97	231	280	192	211	113	133	242	251	153	153	301	302	199	201	130	130	113	126	170	170	
39	93	95	231	237	192	199	110	110	239	241	153	157	302	302	199	213	154	154	113	115	170	170	
39	91	95	231	279	155	178	108	115	252	255	153	157	302	302	209	211	130	131	111	124	168	172	
39	93	97	231	280	192	211	108	137	242	249	153	157	302	302	201	213	130	130	113	117	168	170	B
39	91	91	279	280	155	180	110	113	237	248	153	153	302	302	209	209	130	130	113	113	168	176	
39	93	95	231	280	161	178	108	110	239	255	153	157	302	302	209	211	130	150	105	120	168	168	

39	91	91	280	280	168	188	108	123	241	245	153	157	300	302	209	211	131	150	113	124	166	194	
39	95	97	231	231	178	192	102	108	241	255	153	157	302	302	211	213	131	150	119	124	168	170	
39	95	109	228	231	176	178	108	117	240	245	153	153	302	302	209	211	131	152	113	124	168	172	
39	87	109	231	280	155	190	102	121	243	247	153	157	302	304	211	213	130	157	115	119	156	168	
39	91	91	231	280	180	209	117	121	241	247	153	157	301	301	209	211	131	131	111	122	164	170	
39	89	93	231	279	190	199	110	121	239	241	153	153	302	302	209	211	130	167	111	113	168	170	
39	91	91	237	280	180	192	102	123	225	239	153	153	301	302	209	215	140	148	111	119	168	168	
39	93	95	231	231	155	174	106	108	241	245	153	153	302	319	209	217	129	131	113	126	168	170	
39	95	99	240	280	195	199	108	121	239	243	153	157	302	302	211	215	130	130	126	130	168	170	
39	89	91	231	237	178	211	110	127	237	246	157	161	302	304	199	211	127	171	111	119	166	170	
39	91	95	231	237	184	203	102	117	237	241	157	161	302	302	203	215	129	130	111	113	164	168	
39	107	109	231	237	182	196	123	129	241	249	153	161	292	301	209	209	130	138	111	113	170	170	
40	91	95	231	250	166	168	110	110	245	245	153	153	299	301	209	213	130	130	111	115	168	170	
40	95	101	225	231	166	172	102	121	237	252	153	153	301	301	215	215	130	148	111	122	168	174	
40	91	95	231	250	168	168	121	135	239	245	157	157	301	305	209	215	130	167	115	122	168	168	
40	91	95	231	231	172	192	119	137	237	249	157	157	301	301	203	203	130	157	111	122	168	170	B
40	91	93	219	231	201	222	102	139	239	252	153	157	301	301	203	211	130	131	115	119	170	174	
40	93	97	231	280	172	192	102	110	243	249	157	157	301	301	199	211	130	135	119	120	164	172	
40	93	93	231	231	178	178	102	110	247	252	157	157	301	301	215	215	130	130	111	122	164	170	
40	95	101	231	234	180	184	102	113	229	256	153	157	301	301	203	203	130	131	113	120	170	170	
40	93	95	219	237	155	155	102	119	239	241	157	157	301	303	211	215	130	130	111	115	170	170	
40	93	95	231	237	180	182	108	108	241	245	157	157	301	301	203	211	150	154	115	122	168	168	
40	93	99	219	231	199	240	102	121	241	243	157	157	301	314	213	215	130	131	111	111	168	170	
40	91	95	237	280	178	213	106	121	239	245	157	157	301	301	203	203	131	154	115	122	168	170	
40	93	97	237	280	178	197	110	125	237	239	153	153	301	301	207	213	130	131	120	128	168	170	
40	89	93	231	237	205	205	102	106	249	252	153	161	301	303	213	213	130	130	119	124	168	170	B
40	93	95	231	280	186	190	123	131	239	241	153	161	301	317	199	203	130	130	111	120	168	170	
40	97	99	231	237	186	197	113	129	241	241	153	153	301	301	203	213	131	159	122	132	166	168	
40	93	99	231	280	155	189	119	121	241	242	145	153	301	303	199	229	130	159	115	126	158	168	
40	93	99	231	231	178	192	102	108	247	254	149	157	301	303	209	213	127	154	111	122	170	170	
40	91	91	231	237	195	215	106	123	245	247	153	157	301	303	199	209	130	157	111	111	170	174	
40	89	91	219	231	172	182	102	102	243	255	153	153	301	301	213	215	130	169	111	113	168	170	
40	91	93	219	231	178	222	102	108	237	245	153	157	301	301	211	213	130	157	111	115	170	174	
40	91	93	231	280	172	189	119	121	239	248	157	157	301	301	199	199	130	148	122	126	168	170	
40	89	91	231	240	166	182	115	131	243	245	157	157	301	301	209	209	157	165	111	111	168	168	
40	87	91	231	280	180	180	119	121	239	257	153	153	301	301	213	217	130	154	128	132	168	170	B
40	87	91	231	231	168	207	102	115	245	248	153	157	303	303	205	215	131	148	111	113	164	170	
40	95	95	231	244	155	174	102	110	245	248	161	161	301	303	209	209	130	130	115	115	166	168	
40	91	95	231	231	174	205	108	108	239	239	153	153	301	301	203	203	130	131	115	124	168	170	
40	91	99	231	231	180	228	108	121	247	254	149	157	301	303	209	209	130	130	122	126	168	172	
40	97	101	231	280	157	197	108	110	245	252	161	161	303	303	215	215	130	169	115	115	164	164	
40	87	91	231	280	180	186	119	121	239	257	153	153	301	301	213	217	130	154	128	132	168	170	
40	93	93	231	280	172	201	102	102	245	249	153	157	301	309	213	215	130	131	126	128	164	168	
40	87	91	231	231	168	207	102	115	245	248	153	157	303	303	205	215	131	148	111	113	164	170	
40	91	91	231	237	172	180	110	121	241	245	153	161	301	303	209	213	131	131	115	128	168	170	
40	91	93	231	280	174	192	108	113	241	245	153	153	301	301	215	215	130	131	111	128	168	170	B
40	101	101	228	231	182	190	110	135	237	251	153	157	301	310	203	211	130	130	117	120	166	174	
40	89	91	234	280	176	207	108	135	241	245	157	157	299	301	203	217	131	177	113	117	168	174	
40	97	105	237	280	184	197	102	102	242	243	157	161	301	301	209	213	131	131	111	130	168	172	
40	91	101	231	231	172	184	102	110	252	253	153	157	300	301	211	211	127	133	115	115	168	168	
40	91	101	231	280	178	178	108	121	239	253	153	161	301	303	203	211	131	165	102	115	168	168	
40	97	101	231	231	166	197	102	117	239	245	153	153	302	303	199	211	129	131	120	124	168	172	
40	101	101	231	237	184	195	108	110	241	248	153	153	302	302	203	209	130	131	111	126	164	170	
40	91	91	231	231	168	178	102	106	241	247	153	153	301	303	209	211	130	130	115	132	166	168	
40	91	93	219	280	180	182	108	121	237	245	153	161	301	301	203	209	131	161	115	115	164	168	
40	87	91	231	237	199	211	106	121	239	244	157	161	301	301	213	215	130	133	111	113	158	170	
40	87	91	234	280	186	186	108	110	241	247	157	157	305	305	209	213	131	167	113	115	164	164	
40	97	97	231	231	172	180	108	115	239	239	153	161	301	301	199	209	130	131	124	124	168	172	
40	89	95	237	280	157	178	108	119	248	249	153	153	302	302	199	203	130	159	111	115	166	170	
41	91	93	237	280	176	188	110	110	239	249	149	157	302	302	209	209	130	130	111	126	166	166	
41	93	99	237	280	188	201	110	113	241	241	153	157	302	314	209	213	130	130	111	130	166	170	
41	87	87	231	237	176	201	102	113	237	247	157	157	302	302	209	215	127	183	113	115	164	170	
41	91	93	231	240	155	199	110	119	237	243	153	157	302	302	203	211	130	130	120	126	164	168	B
41	91	93	231	240	155	178	102	117	243	249	153	153	302	302	199	211	130	131	119	120	158	168	
41	91	91	231	240	201	217	110	123	239	245	153	157	302	314	199	207	130	159	113	119	168	184	
41	95	95	231	280	178	186	110	117	241	249	161	161	314	314	211	215	130	167	122	122	158	164	
41	91	93	225	231	199	201	106	119	239	249	153	153	301	304	203	213	135	152	119	126	168	170	
41	87	93	207	207	155	163	100	113	233	239	149	153	301	303	199	215	130	144	122	126	156	176	
41	87	87	240	280	163	203	117	117	237	245	153	153	302	302	209	211	131	146	122	128	170	170	
41	91	99	231	231	155	163	117	139	246	256	157	157	302	302	209	215	133	135	122	128	168	170	
41	87	95	231	231	178	199	102	121	247	256	157	157	302	302	209	209	130	130	111	124	170	170	
41	87	91	231	240	163	203	113	117	245	256	149	157	302	313	209	215	130	135	122	126	156	170	
41	91	93	231	280	163	178	102	117	245	249	153	161	302	302	215	215	130	157	113	113	170	174	B
41	91	91	219	240	155	190	102	102	247	255	157	157	302	302	209	215	130	154	111	122	170	176	
41	91	91	231	280	166	188	108	133	237	247	157	157	301	301	201	201	129	130	113	115	168	168	
41	91	99	280	280	180	192	110	110	245	249	157	157	301	301	209	213	130	154	113	130	164	168	
41	91	91	237	280	192	201	110	110	241	249	157	157	301	301	211	211	131	167	115	119	170	172	
41	95	99	231	237	175	199	110	127	245	249	153	157	303	311	203	209	130	138	113	122	164	170	
41	91	95	228	231	182	182	117	121	239	245	153	153	301	303	209	209	130	130	119	126	168	170	
41	95	107	231	240	166	205	108	108	235	239	153	157	301	301	203	211	130	130	115	122	170	170	
41	91	95	237	237	199	211	102	135	242	249	157	161	301	311	199	209	127	130	113	113	164	168	
41	87	91	231	237	166	186	108	110	239	239	153	157	301	301	217	217	130	130	126	132	166	170	
41	91	91	237	237	182	186	102	110	241	245	153	157	301	301	209	209	130	181	113	126	168	170	B
41	91	103	225	280	184	186	106	110	241	247	153	157	301	301	203	203	130	133	111	120	166	166	
41	93	95	219	237	155	219	125	127	239	247	153	153	301	324	211	213	130	154	117	132	164	170	
41	91	93	231	280	166	182	106	108	239	248	153	157	302	302	203	209	130	131	102	124	164	168	
41	91	109	237	280	186	188	102	121	235	245	153	153	302	302	203	219	130	154	113	124	170	190	
41	93	93	228	231	176	197	108	110	239	247	153	153	301	301	203	217	130	131	102	130	164	170	
41	91	99	231	234	186	195	108	113	245	249	153	153	301	301	199	199	130	150	111	115	170	172	
41	91	97	237	280	174	190	110	123	239	239	153	153	301	301	203	217	130	130	111	115	166	170	
41	91	91	240	280	155	186	102	125	239	239	153	153	301	301	213	217	133	165	111	111	166	168	
41	91	91	240	280	184	188	102	119	247	251	153	153	301	310	199	217	133	159	113	119	168	168	
41	93	95	237	280	195	201	108	113	247	249	153	153	301	301	203	225	130	130	113	115	168	190	B
41	91	91	231	240	184	203	117	125	245	257	153	157	301	304	209	217	130	138	124	126	164	168	
41	87	103	225	280	186	203	102	131	243	253	153	153	280	288	209	215	148	152	111	122	166	168	
41	87	95	225	225	168	195	106	131	233	249	153	153	303	303	203	209	130	130	113	126	166	168	
41	91	93	231	231	186	234	100	108	229	245	153	153	301	301	203	213	130	131	115	126	168	170	
41	91	93	231	240	172	172	113	129	247	252	153	153	301	301	209	225	130	130	122	132	166	170	
41	93	93	237	240	185	192	110	110	239	242	153	161	301	301	199	199	130	130	113	120	172	174	
41	93	109	225	280	161	178	117	129	229	239	153	153	301	301	203	203	131	165	113	132	168	170	
41	91	99	228	231	155	174	110	117	241	243	153	153	305	305	211	213	130	167	113	119	170	170	
41	91	97	231	280	155	190	100	129	243	253	153	157	301	304	203	209	130	200	126	128	168	170	
41	93	93	231	231	190	207	102	129	239	249	153	153	301	301	209	213	130	131	113	120	168	170	
41	89	93	231	280	180	207	110	110	239	243	153	153	301	301	213	217	144	167	111	115	170	190	
41	91	91	231	237	155	201	110	119	241	249	153	157	301	303	205	211	130	131	113	119	168	170	
41	91	107	231	231	186	207	110	121	243	245	153	161	301	303	213	213	130	130	105	111	164	164	

41	91	97	231	237	178	182	102	121	249	257	153	157	300	301	199	211	130	130	119	120	170	170	
42	93	109	231	231	176	180	108	121	248	250	153	153	302	302	203	215	130	161	113	113	170	172	A
42	91	95	231	231	155	180	102	102	245	254	153	153	302	305	199	207	127	135	109	113	166	176	
42	93	97	231	280	176	176	108	121	249	249	153	153	302	302	207	215	130	130	105	126	168	170	
42	91	95	231	231	180	180	106	121	239	248	153	153	302	304	199	207	130	148	136	144	172	172	
42	87	95	237	280	178	192	117	121	245	248	153	157	302	302	211	215	130	159	119	128	166	170	
42	95	95	231	280	180	180	102	108	243	248	153	157	302	305	199	215	130	130	136	144	172	172	
42	89	91	231	231	176	176	108	113	241	241	153	157	302	302	209	215	130	130	120	122	170	194	
42	91	91	231	231	176	201	108	113	242	247	153	157	299	302	207	209	130	130	119	122	166	170	
42	91	93	231	280	195	199	108	113	242	247	153	157	302	302	207	213	131	157	113	126	166	168	
42	91	101	231	280	195	199	102	113	237	253	153	157	286	302	209	219	129	129	119	120	167	167	
42	93	95	231	231	188	199	102	102	237	239	153	153	302	302	209	211	154	159	115	115	168	168	A
42	91	95	240	280	180	188	106	106	237	241	157	161	302	302	199	211	130	130	111	120	168	168	
42	91	93	231	237	188	199	119	135	239	239	153	153	302	302	211	213	130	154	120	122	168	168	
42	91	91	231	234	155	201	113	121	239	253	157	157	302	308	194	219	130	130	113	122	168	170	
42	95	101	231	280	155	188	106	121	245	249	153	161	302	304	209	211	130	187	113	126	170	170	
42	91	95	231	231	174	176	108	119	239	239	153	157	302	302	215	219	129	159	111	126	166	170	
42	95	103	231	231	155	176	108	108	241	249	153	153	302	302	205	211	130	159	111	117	167	167	
42	91	91	231	237	182	213	110	119	239	248	145	153	302	302	211	211	130	135	115	119	168	170	
42	91	93	228	234	176	188	143	151	243	243	149	153	302	302	199	199	127	130	111	113	168	172	
42	93	95	228	240	184	201	102	108	245	257	153	157	302	302	199	203	127	130	120	124	158	168	
42	91	93	231	237	166	182	108	117	237	243	153	157	290	304	211	211	130	135	113	144	167	168	A
42	91	91	231	280	155	192	113	137	239	253	153	153	302	302	203	211	127	135	115	122	168	170	
42	89	95	228	240	182	201	110	121	245	257	153	161	302	302	203	211	130	131	111	128	164	167	
42	89	89	231	280	155	184	108	110	237	245	153	161	290	302	211	211	130	135	111	128	168	170	
42	91	91	231	231	155	201	110	121	239	239	153	157	302	302	203	203	127	131	111	124	168	168	
42	93	93	228	237	201	213	108	108	241	245	153	153	302	302	199	203	127	163	111	132	168	168	
42	89	93	228	240	178	180	117	121	239	243	157	157	302	302	203	209	127	131	111	111	168	170	
42	91	93	231	231	184	192	102	108	239	245	153	153	302	302	203	203	130	130	117	120	164	170	
42	93	95	231	240	195	199	119	119	239	245	153	157	302	302	211	215	131	159	115	128	164	172	
42	93	101	228	231	195	213	102	131	239	242	153	153	302	302	209	209	129	131	113	122	164	168	
42	93	97	237	280	192	197	106	106	239	239	153	157	302	306	209	209	130	130	122	126	170	170	A
42	87	93	231	231	176	180	102	131	245	251	153	153	302	302	199	207	127	129	111	136	168	172	
42	91	95	225	231	180	207	106	121	245	248	153	157	302	302	213	215	130	131	115	136	168	172	
42	93	97	234	240	180	180	121	131	239	243	153	153	302	302	209	215	130	130	113	120	168	174	
42	91	93	231	240	166	182	117	133	243	247	153	153	302	302	201	215	130	138	115	128	166	168	
42	97	99	231	240	155	182	133	135	241	249	153	153	302	302	209	211	130	131	105	115	170	174	
42	91	91	225	237	163	182	131	133	239	243	153	157	302	302	209	213	138	154	113	113	170	174	
42	87	93	231	231	180	186	102	117	245	248	153	157	298	305	207	211	127	130	113	119	168	176	
42	95	95	231	231	172	186	102	117	237	243	153	157	302	306	203	207	130	130	113	113	170	194	
42	93	95	231	231	176	195	110	123	235	243	153	153	302	302	203	209	130	161	111	119	162	170	
42	95	95	240	240	176	176	102	113	229	245	153	157	302	302	199	203	130	131	105	111	170	172	
42	91	91	225	240	182	199	121	135	237	243	153	153	302	302	213	215	130	138	115	124	168	170	
42	87	91	231	231	176	207	123	131	229	257	153	157	301	301	199	209	130	130	105	111	168	170	
42	87	97	231	231	184	213	108	119	243	251	153	157	302	302	199	215	130	173	113	126	164	168	
42	87	99	231	237	178	184	110	123	248	252	153	153	302	302	203	217	130	154	113	122	170	170	
42	91	95	231	234	176	209	102	113	239	250	153	157	290	302	199	209	129	169	111	120	166	168	
42	97	97	231	234	186	188	108	108	239	244	153	157	306	315	199	201	130	159	111	115	166	170	
42	87	91	231	280	209	213	102	137	239	245	153	153	302	302	203	209	130	130	117	122	172	172	
42	91	91	228	240	166	166	108	110	241	247	153	157	302	302	199	205	130	130	111	117	168	172	
43	93	95	280	280	166	184	108	129	240	245	149	154	302	302	205	211	130	131	115	132	168	168	
43	89	99	231	280	155	166	110	110	249	255	153	157	302	302	209	213	130	130	111	111	164	168	
43	93	97	240	280	176	186	102	108	247	250	153	153	302	302	213	217	130	130	105	111	166	170	
43	89	91	231	280	176	176	121	145	241	250	153	153	302	304	203	217	130	149	105	111	170	174	
43	91	97	231	280	166	190	121	121	241	241	153	153	302	304	209	209	130	130	105	124	166	170	
43	89	91	237	240	166	199	110	110	241	241	157	157	302	304	207	207	127	130	111	132	168	172	
43	91	95	231	240	155	184	102	113	247	247	153	153	302	302	221	221	130	130	111	130	170	170	
43	87	97	231	280	176	182	119	121	247	249	153	153	302	302	217	217	130	130	126	128	170	174	
43	89	89	231	237	188	188	102	110	241	241	153	153	319	319	203	215	130	131	111	128	168	170	
43	95	95	231	280	172	197	108	135	241	247	153	153	302	319	215	215	130	130	111	132	170	174	A
43	87	97	231	231	166	190	110	110	245	247	153	153	302	302	203	213	130	130	113	126	170	172	
43	87	87	280	280	176	178	108	121	249	251	153	153	302	315	203	211	130	148	115	126	168	168	
43	93	97	280	280	176	192	106	108	241	253	153	153	304	304	203	209	130	130	113	117	164	172	
43	89	91	231	231	176	188	108	151	241	245	153	157	302	304	203	209	130	130	111	119	168	172	
43	91	97	280	280	176	176	121	123	241	247	153	157	302	302	209	211	130	130	119	130	170	172	
43	87	95	228	280	180	201	106	151	245	253	153	157	302	302	199	215	130	130	111	115	168	176	
43	91	97	280	280	182	186	108	123	245	253	153	157	304	304	203	211	130	157	111	119	166	172	
43	91	97	237	280	166	176	121	123	242	245	153	153	302	304	203	213	130	130	111	113	164	172	
43	91	97	231	280	186	188	108	110	245	245	157	157	302	302	203	203	130	130	111	119	172	172	
43	89	91	231	231	178	188	110	131	241	245	157	161	302	304	203	203	133	175	126	130	164	168	A
43	89	97	225	228	176	203	106	151	249	253	153	161	302	302	211	213	130	130	113	119	168	176	
43	89	97	231	231	176	215	121	137	241	245	153	161	304	304	209	215	130	131	120	122	168	172	
43	93	95	231	280	192	203	102	125	245	249	157	157	302	302	209	215	130	181	115	119	168	170	
43	89	99	231	280	155	174	113	137	249	253	153	153	302	302	211	213	130	130	113	122	168	168	
43	91	93	231	280	178	188	108	110	247	253	153	157	302	304	199	211	130	175	111	113	167	170	
43	91	103	231	237	176	182	102	110	243	249	153	157	302	305	205	211	131	133	111	111	168	168	
43	91	99	231	280	182	209	110	121	243	249	153	153	302	304	211	215	131	133	111	120	167	192	
43	89	89	225	231	205	213	102	110	243	245	153	153	302	302	203	209	131	175	113	128	167	170	
43	97	97	231	237	178	224	110	113	241	241	153	161	302	302	209	215	130	135	111	119	168	174	
43	87	89	231	280	178	213	110	123	245	249	153	161	302	302	203	203	130	130	111	113	167	167	A
43	87	87	231	231	168	195	106	106	245	249	153	161	302	302	213	215	130	165	105	126	170	174	
43	91	97	240	280	155	186	108	110	241	241	153	161	302	304	209	209	130	130	111	111	164	172	
43	87	91	231	231	168	178	110	151	239	249	153	153	302	302	203	211	130	148	119	128	164	174	
43	91	95	228	237	166	178	121	121	239	251	153	153	302	302	203	215	130	149	111	126	166	168	
43	87	97	237	237	192	192	106	106	245	253	153	153	302	302	205	221	130	130	111	126	166	168	
43	95	95	237	240	178	188	108	110	237	245	153	161	302	319	199	203	130	154	111	113	170	176	
43	91	91	231	280	190	195	113	123	241	245	153	153	302	306	211	215	154	171	111	122	168	170	
43	91	97	231	237	178	188	102	108	241	249	153	161	302	302	203	209	130	133	111	113	168	170	
43	91	97	231	280	188	213	108	108	241	253	153	161	302	302	203	207	130	131	113	122	168	170	
43	95	97	225	231	176	178	102	125	241	241	153	153	302	302	199	203	131	131	113	122	164	166	A
43	91	95	231	231	188	195	113	113	241	245	153	153	302	302	203	213	131	154	111	128	168	170	
43	95	99	231	231	178	188	102	102	241	241	153	153	302	302	203	203	131	133	128	130	168	168	
43	89	95	231	280	176	188	102	113	241	247	153	153	302	302	209	217	130	130	105	128	168	174	
43	95	103	231	240	178	178	113	121	241	243	153	153	302	302	203	209	130	131	111	128	166	166	
43	89	95	231	231	178	188	102	113	241	241	153	153	302	302	203	209	130	131	113	128	166	168	
43	89	95	231	234	166	186	108	110	241	247	157	157	302	302	209	209	130	135	111	117	164	168	
43	89	95	231	231	178	188	102	113	241	241	153	153	302	302	203	209	130	131	113	128	166	168	
44	93	97	240	280	176	204	102	115	245	246	153	153	302	302	209	211	127	130	111	113	168	176	B
44	95	97	231	237	180	195	102	121	245	249	157	157	302	302	211	213	130	131	111	126	164	170	B
44	95	99	231	280	180	195	110	121	245	249	153	153	302	302	209	229	130	130	113	117	166	170	B
44	89	91	231	231	174	180	102	115	244	253	153	153	302	302	203	213	127	130	113	113	170	170	B
44	91	91	237	280	155	155	108	110	243	249	153	153	302	302	211	211	129	138	111	120	164	176	B
44	89	95	231	240	195	207	106	110	239	243	153	157	302	302	211	211	131	131	115	119	170	170	A
44	91	91	237	280	155	155	108	110	243	249	153	153	302	302	211	211	129	138	111	120	164	176	B
44	91	103	231	240	174	207	110	115	239	246	153	157	302	302	203	211	127	131	113	128	170	170	A
44	89	91	240	240	155	174	110	121	239	246	153	153	302	302	211	211	131	131	113	115	170	170	A

44	89	97	231	234	195	207	110	139	237	239	153	153	302	302	211	215	131	131	115	117	170	174	A
44	91	93	231	237	174	176	108	121	237	246	153	153	302	302	211	215	131	133	113	113	168	170	B
44	89	91	240	240	174	207	110	115	251	255	153	157	302	302	203	211	127	131	113	128	170	170	A
44	95	101	222	231	178	190	102	102	241	249	153	157	302	302	0	0	131	146	113	117	168	172	B
44	87	99	231	280	192	205	133	135	247	249	157	161	302	302	209	209	130	146	113	130	167	170	
44	95	101	240	280	197	205	102	121	248	253	153	157	302	304	215	215	127	152	111	128	167	167	
44	91	91	231	231	186	203	108	129	239	247	153	157	302	302	209	209	130	133	120	128	166	168	
44	91	95	225	280	176	190	125	135	229	239	157	161	302	302	211	215	130	131	105	115	168	170	
44	99	99	231	280	176	205	102	106	243	249	153	157	302	302	209	209	146	175	120	128	164	168	
44	91	91	225	231	176	186	108	139	243	249	153	161	302	302	205	209	131	163	115	122	166	176	
44	91	99	237	280	155	176	119	121	233	249	145	153	302	302	199	211	130	131	115	117	164	170	
44	91	95	231	280	155	176	108	131	249	249	153	153	302	302	209	209	131	161	115	134	166	168	
44	91	95	231	280	180	184	113	121	243	245	153	153	302	302	203	213	138	165	113	117	168	168	
44	91	99	231	280	173	190	121	125	237	241	161	161	302	302	209	223	130	159	111	115	166	168	
44	91	97	231	231	176	176	106	139	237	241	153	161	302	302	209	209	131	131	119	130	168	168	
44	91	91	234	280	155	195	125	125	237	244	157	161	302	328	203	215	159	175	105	111	168	168	
44	91	95	225	231	174	190	102	125	237	249	153	161	302	302	209	213	130	131	105	113	166	168	
44	91	109	231	231	176	176	102	139	239	249	153	161	302	302	209	209	130	131	128	128	168	170	
44	89	99	231	280	176	199	106	131	239	241	153	153	302	302	217	229	130	130	113	119	168	180	
44	97	99	231	231	176	195	108	110	241	249	153	161	302	302	203	211	130	130	113	122	166	168	
44	91	95	237	280	176	186	108	123	241	249	153	153	302	302	211	223	130	130	115	122	166	168	
44	95	99	231	237	190	207	102	110	241	249	161	161	302	302	203	211	131	131	115	124	166	168	
44	93	95	237	280	176	190	102	119	241	249	153	161	302	302	211	215	130	131	122	126	168	170	
44	99	119	231	280	176	178	102	119	241	249	153	153	302	302	199	211	130	167	120	122	168	170	
44	95	99	231	231	184	207	102	121	229	249	153	161	302	302	209	211	130	163	115	122	166	166	
44	95	97	225	231	176	184	110	110	229	257	161	161	302	302	209	211	130	131	124	132	166	170	
44	95	97	231	231	155	180	110	135	243	245	153	153	301	304	215	219	165	171	120	136	164	168	B
44	93	95	231	280	176	180	110	121	237	247	153	157	302	302	209	209	131	154	124	126	164	170	B
44	93	95	231	280	180	186	110	151	247	247	149	161	302	302	197	209	129	154	111	113	164	172	
44	89	97	231	234	153	199	110	113	239	245	153	153	302	302	211	213	130	138	115	126	170	172	
44	95	97	231	280	180	197	113	125	237	239	153	161	302	302	209	213	130	130	115	120	168	170	
44	91	97	231	280	186	197	110	113	239	247	153	153	302	319	199	213	130	169	115	119	170	170	
44	93	95	231	280	163	197	106	119	233	241	153	157	302	302	205	213	130	130	126	150	168	170	
44	97	101	231	280	176	182	123	139	239	239	153	153	302	302	209	221	130	154	111	124	168	170	
44	93	117	231	231	163	163	110	117	243	249	153	157	302	302	211	215	130	131	113	122	164	168	
44	87	91	231	280	176	188	110	139	239	249	153	153	302	302	211	213	130	130	120	124	164	166	
44	91	93	231	231	155	176	113	147	237	251	153	161	302	302	203	209	133	133	113	115	166	176	
44	91	97	225	231	166	180	108	123	249	251	153	161	302	304	203	209	130	131	111	120	166	166	
44	87	91	231	231	182	195	115	127	243	249	153	153	304	306	215	215	130	130	111	111	166	166	B

0: missing data
