# Supplementary material for: Possible northern persistence of Siebold’s beech, Fagus crenata, at its northernmost distribution limit on an island in Japan Sea: Okushiri Island, Hokkaido
Source: Front Plant Sci. 2022 Dec 15;13:990927. doi: 10.3389/fpls.2022.990927 (PMC9797532; doi:10.3389/fpls.2022.990927)
Supplement: Supplementary file 4 [file DataSheet_4.pdf]

Supplementary Table 4. Null allele frequencies estimated by CERVUS .

Site ID	FS1-03	FS4-46	mfc2	sfc36	sfc378	sfc7	mfc12	sfc1063	sfc1105	sfc1143	sfc18
1	0.013	-0.037	-0.026	-0.009	0.031	0.035	-0.015	0.050	0.032	0.015	0.002
2	-0.037	0.042	0.012	0.032	0.001	-0.015	0.003	0.012	-0.041	-0.008	-0.038
3	-0.013	0.078	-0.019	-0.024	0.001	-0.112	0.024	-0.018	0.043	0.004	-0.025
4	-0.007	0.017	-0.017	-0.059	-0.015	-0.118	0.130	0.036	-0.066	0.014	-0.084
5	-0.047	0.011	0.013	-0.051	-0.013	-0.097	0.069	-0.001	-0.022	0.021	0.007
6	-0.024	-0.035	-0.023	0.010	-0.007	0.175	0.085	0.011	0.016	-0.027	-0.081
7	0.003	0.041	0.014	-0.017	-0.044	-0.030	0.147	0.070	-0.085	-0.031	-0.001
8	0.000	0.037	0.008	0.028	0.039	0.061	0.278 *	-0.069	0.049	-0.005	-0.093
9	0.015	0.074	0.003	-0.004	0.054	0.093	0.068	0.003	-0.010	-0.007	0.025
10	-0.041	0.029	-0.014	-0.005	-0.054	0.013	-0.031	-0.045	-0.023	0.045	-0.054
11	-0.081	-0.022	-0.010	0.008	-0.040	0.024	-0.029	-0.010	-0.030	0.005	-0.024
12	-0.064	-0.005	-0.045	-0.073	-0.007	-0.037	0.027	0.092	0.006	0.094	-0.026
13	-0.028	-0.017	0.015	-0.032	-0.016	-0.052	0.084	0.050	0.006	-0.028	0.025
14	-0.010	0.043	0.015	0.002	-0.045	-0.063	-0.058	-0.036	0.011	-0.039	0.016
15	-0.027	0.046	0.016	-0.038	-0.026	-0.001	-0.011	0.021	0.016	-0.017	-0.042
16	0.012	0.190	-0.050	-0.021	-0.038	0.440	-0.041	0.016	-0.161	0.038	0.037
17	0.031	-0.105	0.010	0.003	-0.063	0.068	0.120	-0.006	-0.008	0.025	-0.037
18	0.008	0.019	0.015	0.047	-0.005	0.129	0.191	0.006	-0.001	-0.028	-0.079
19	-0.047	-0.046	0.008	-0.020	0.022	0.010	0.186	-0.023	-0.050	0.017	0.002
20	-0.075	-0.053	-0.035	0.051	0.035	-0.110	0.148	-0.014	-0.038	0.039	-0.007
21	0.123	0.120	-0.023	0.062	0.082	0.205	0.070	-0.040	-0.052	-0.106	0.034
22	0.012	-0.073	-0.024	-0.057	-0.040	-0.147	0.113	0.006	-0.012	-0.035	0.044
23	-0.042	0.001	0.023	-0.022	0.022	-0.060	0.262	-0.043	-0.032	-0.037	0.027
24	-0.012	0.013	-0.007	0.033	-0.032	0.034	0.043	-0.019	-0.016	0.016	-0.025
25	0.047	-0.035	-0.021	0.007	0.027	0.068	0.115	0.003	-0.025	0.017	0.027
26	0.037	-0.016	-0.008	-0.022	-0.001	0.013	0.007	-0.036	-0.026	-0.017	-0.016
27	-0.010	-0.032	-0.012	-0.035	-0.016	-0.067	-0.044	-0.035	-0.078	-0.058	0.011
28	-0.013	0.010	-0.017	0.019	-0.042	-0.084	0.422	-0.074	0.024	-0.012	-0.060
29	-0.061	0.014	-0.018	-0.072	-0.096	0.138	0.449	0.040	-0.002	-0.103	-0.121
30	-0.013	-0.029	0.013	-0.037	0.024	-0.010	0.275	0.057	-0.028	-0.010	-0.004
31	-0.025	0.019	-0.009	-0.018	0.043	-0.002	0.067	-0.044	0.051	-0.026	0.090
32	0.027	-0.073	-0.016	0.011	0.133	0.143	0.011	-0.002	-0.015	-0.040	-0.025
33	0.004	-0.027	0.068	-0.039	0.005	0.052	-0.052	-0.045	0.054	-0.029	-0.006
34	-0.077	0.057	0.132	-0.100	-0.066	-0.031	0.007	0.072	-0.027	-0.031	-0.087
35	0.026	0.022	0.014	0.008	-0.017	0.109	0.106	0.045	0.042	-0.001	-0.019
36	-0.003	0.096	-0.001	-0.011	-0.040	-0.004	0.427	0.027	-0.034	0.028	-0.013
37	0.003	-0.035	-0.027	-0.039	-0.040	-0.097	0.147	-0.017	0.011	-0.024	-0.022
38	0.007	-0.079	0.052	-0.002	-0.013	0.199	0.113	0.096	-0.012	0.019	-0.043
39	0.045	-0.009	-0.008	0.033	-0.015	0.138	0.358	0.078	-0.012	-0.015	-0.014
40	-0.055	0.017	-0.009	0.010	-0.006	-0.119	0.183	0.105	0.036	-0.013	-0.110
41	-0.020	-0.030	-0.010	0.013	-0.019	0.049	0.082	-0.026	0.048	-0.056	0.019
42	0.057	0.035	0.039	0.034	0.019	-0.095	0.128	0.019	0.019	0.001	0.035
43	-0.002	0.018	0.007	0.053	0.037	0.101	0.112	0.039	0.027	-0.052	0.003
44	-0.027	-0.057	0.005	-0.020	-0.035	0.034	0.206	0.063	0.055	-0.013	0.036

* A significant deviation from Hardy-Weinber equilibrium ($p < 0.05$)
