# Supplementary material for: Possible northern persistence of Siebold’s beech, Fagus crenata, at its northernmost distribution limit on an island in Japan Sea: Okushiri Island, Hokkaido
Source: Front Plant Sci. 2022 Dec 15;13:990927. doi: 10.3389/fpls.2022.990927 (PMC9797532; doi:10.3389/fpls.2022.990927)
Supplement: Supplementary file 5 [file DataSheet_5.pdf]

Supplementary Table 5. Demographic parameters of Scenario 5 obtained by DIYABC.

Parameter	mean	median	mode	quantile 2.5%	quantile 5%	quantile 95%	quantile 97.5%
N1	7640	7820	8250	4600	5160	9490	9640
N2	5620	5680	5730	2150	2730	8270	8640
N3	8910	9180	9940	6450	6990	9940	9970
t1	2380	1780	55.6	65.8	126	6690	7500
t2	4530	4380	4670	998	1340	8230	8730
t3	4040	3870	2830	431	728	7930	8540
t4	7550	7890	9580	3420	4120	9810	9910
ra	0.582	0.615	0.986	0.0585	0.104	0.966	0.983
rb	0.576	0.615	0.977	0.0402	0.0792	0.967	0.982
rc	0.506	0.505	0.893	0.0272	0.0534	0.951	0.975
Mean mutation rate_SSR	8.35E-04	8.68E-04	1.00E-03	4.98E-04	5.63E-04	9.92E-04	9.99E-04
Mean *P**	2.32E-01	2.44E-01	3.00E-01	1.17E-01	1.29E-01	2.98E-01	3.00E-01
Mean mutation rate_SNI	1.94E-06	8.69E-07	1.45E-08	1.65E-08	2.38E-08	7.39E-06	8.50E-06

*the parameter of the geometric distribution to generate multiple stepwise mutations
