# Supplementary figures and images for: Possible northern persistence of Siebold’s beech, Fagus crenata, at its northernmost distribution limit on an island in Japan Sea: Okushiri Island, Hokkaido
Source: Front Plant Sci. 2022 Dec 15;13:990927. doi: 10.3389/fpls.2022.990927 (PMC9797532; doi:10.3389/fpls.2022.990927)

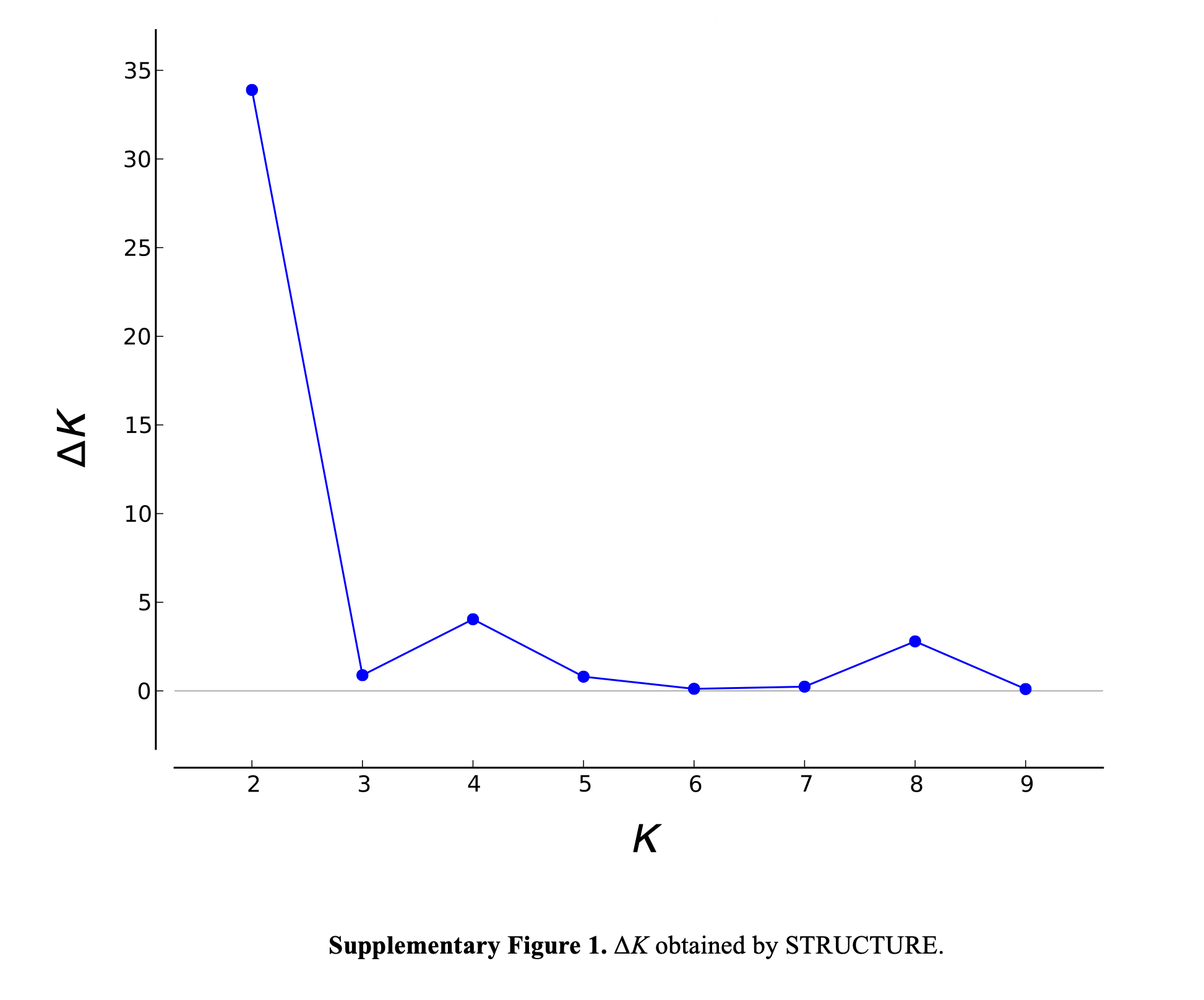

Supplement: Supplementary file 6 [file Image_1.tiff]

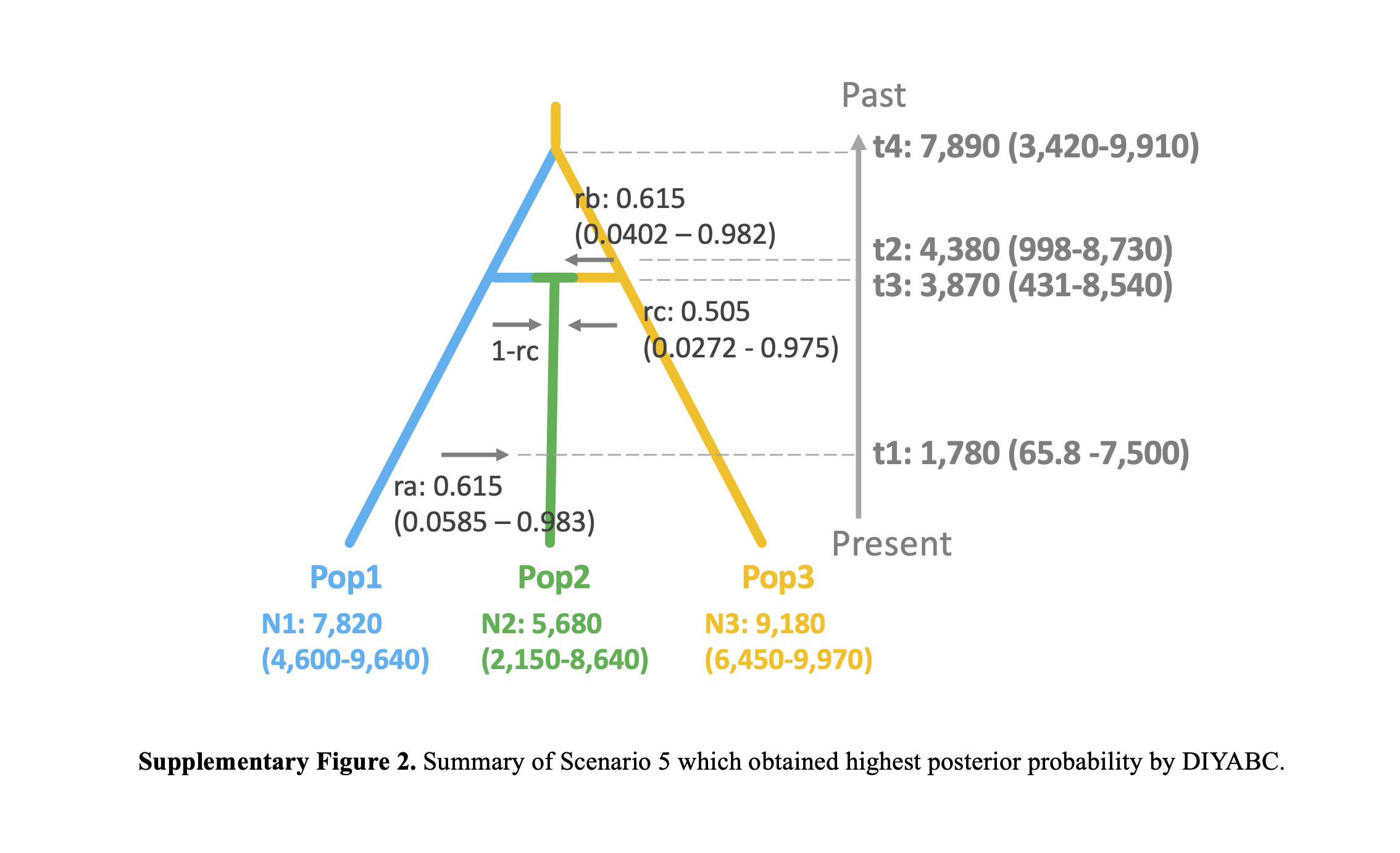

Supplement: Supplementary file 7 [file Image_2.tiff]

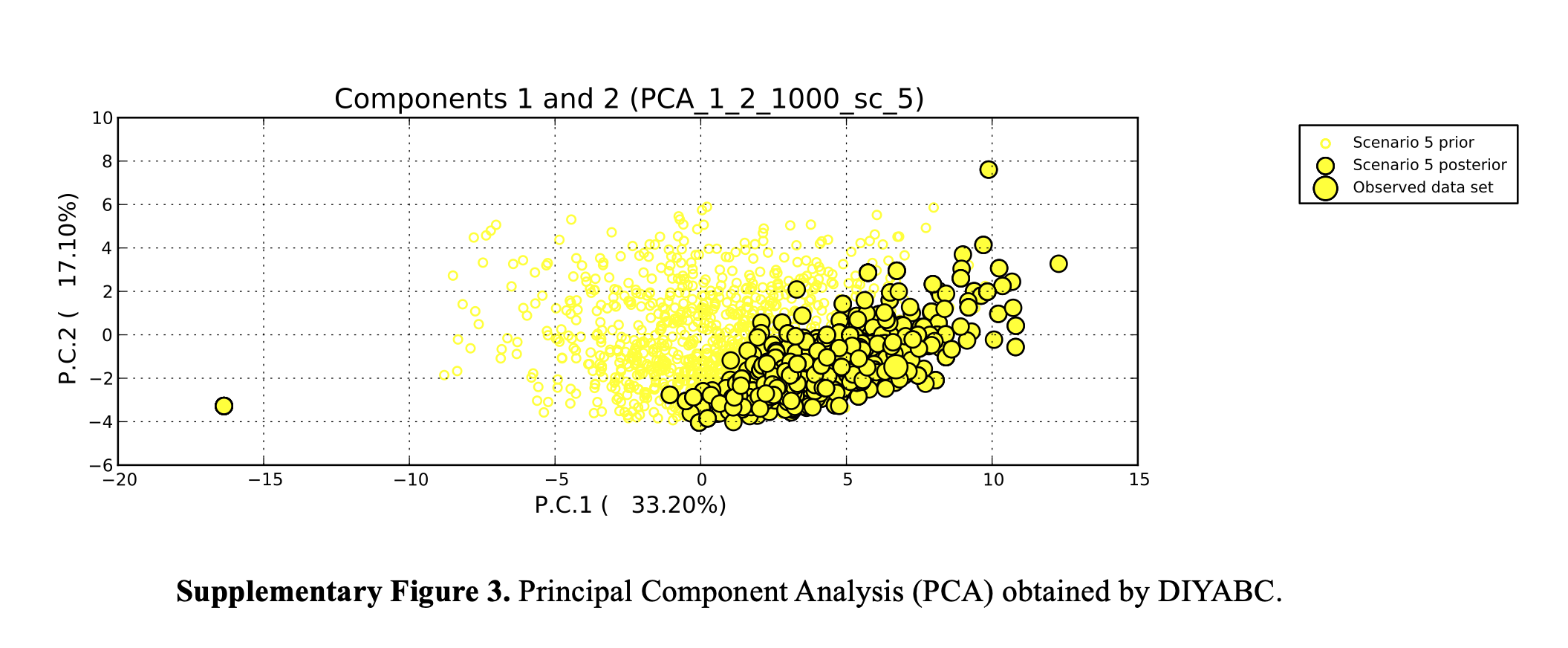

Supplement: Supplementary file 8 [file Image_3.tiff]
